# A first-in-class dual-chelator theranostic agent designed for use with imaging-therapy radiometal pairs of different elements†

**DOI:** 10.1039/d4sc02851a

**Published:** 2024-06-03

**Authors:** James L. Wood, Saikat Ghosh, Zachary H. Houston, Nicholas L. Fletcher, James Humphries, Karine Mardon, Dewan T. Akhter, William Tieu, Alesia Ivashkevich, Michael P. Wheatcroft, Kristofer J. Thurecht, Rachel Codd

**Affiliations:** a The University of Sydney, School of Medical Sciences New South Wales 2006 Australia rachel.codd@sydney.edu.au; b Centre for Advanced Imaging (CAI), Australian Institute for Bioengineering and Nanotechnology (AIBN) and ARC Training Centre for Innovation in Biomedical Imaging Technology, The University of Queensland Brisbane Queensland 4072 Australia; c Molecular Imaging and Therapy Research Unit (MITRU), South Australian Health and Medical Research Institute (SAHMRI) Adelaide Australia; d Telix Pharmaceuticals Limited North Melbourne Victoria 3051 Australia

## Abstract

A covalent adduct of DFOB and DOTA separated by a l-lysine residue (DFOB-l-Lys-*N*^6^-DOTA) exhibited remarkable regioselective metal binding, with {^1^H}-^13^C NMR spectral shifts supporting Zr(iv) coordinating to the DFOB unit, and Lu(iii) coordinating to the DOTA unit. This first-in-class, dual-chelator theranostic design could enable the use of imaging-therapy radiometal pairs of different elements, such as ^89^Zr for positron emission tomography (PET) imaging and ^177^Lu for low-energy β^−^-particle radiation therapy. DFOB-l-Lys-*N*^6^-DOTA was elaborated with an amine-terminated polyethylene glycol extender unit (PEG4) to give DFOB-*N*^2^-(PEG4)-l-Lys-*N*^6^-DOTA (compound D2) to enable installation of a phenyl-isothiocyanate group (Ph-NCS) for subsequent monoclonal antibody (mAb) conjugation (mAb = HuJ591). D2-mAb was radiolabeled with ^89^Zr or ^177^Lu to produce [^89^Zr]Zr-D2-mAb or [^177^Lu]Lu-D2-mAb, respectively, and *in vivo* PET/CT imaging and *in vivo*/*ex vivo* biodistribution properties measured with the matched controls [^89^Zr]Zr-DFOB-mAb or [^177^Lu]Lu-DOTA-mAb in a murine LNCaP prostate tumour xenograft model. The ^89^Zr-immuno-PET imaging function of [^89^Zr]Zr-D2-mAb and [^89^Zr]Zr-DFOB-mAb showed no significant difference in tumour accumulation at 48 or 120 h post injection. [^89^Zr]Zr-D2-mAb and [^177^Lu]Lu-D2-mAb showed similar *ex vivo* biodistribution properties at 120 h post-injection. Tumour uptake of [^177^Lu]Lu-D2-mAb shown by SPECT/CT imaging at 48 h and 120 h post-injection supported the therapeutic function of D2, which was corroborated by similar therapeutic efficacy between [^177^Lu]Lu-D2-mAb and [^177^Lu]Lu-DOTA-mAb, both showing a sustained reduction in tumour volume (>80% over 65 d) compared to vehicle. The work identifies D2 as a trifunctional chelator that could expand capabilities in mixed-element radiometal theranostics to improve dosimetry and the clinical outcomes of molecularly targeted radiation.

## Abstract 2

Covalent DFOB-DOTA dual chelator (‘D2’) binds ^89^Zr (DFOB) and ^177^Lu (DOTA) regio-selectively enabling ^89^Zr–^177^Lu pairing as a novel cancer-targeting theranostic.

## Graphical abstract

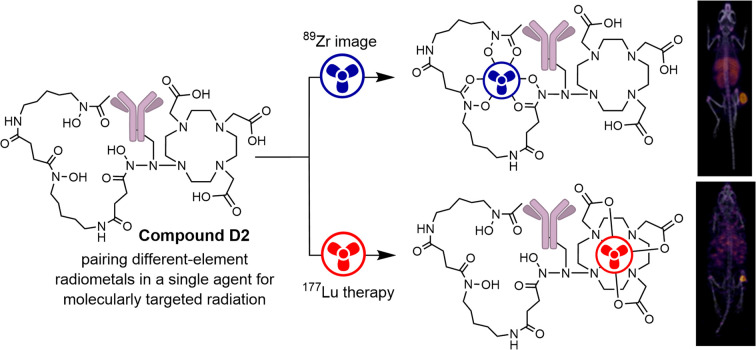

## Introduction

The use of radiometals for targeted imaging and therapy in nuclear medicine is expanding,^1–7^ as evident by recent U.S. Food and Drug Administration approvals for ^177^Lu *endo*-radiotherapy agents Lutathera® for neuroendocrine tumours (NET)‡‡Abbreviations: mCRPC, metastatic castration-resistant prostate cancer; DFOB, desferrioxamine B; DOTA, 1,4,7,10-tetraazacyclododecane-1,4,7,10-tetraacetic acid; HBED-CC, 3,3′-(((ethane-1,2-diylbis((carboxymethyl)azanediyl))bis(methylene)) bis(4-hydroxy-3,1-phenylene))dipropionic acid; NET, neuroendocrine tumours; PSMA, prostate-specific membrane antigen. and Pluvicto™ for metastatic castration-resistant prostate cancer (mCRPC). A major clinical advantage of these agents is afforded by the ability to individualise the dose of β^−^-particle radiation therapy by first measuring the tumour burden using an imaging agent containing the same targeting vector as the therapeutic agent (*e.g.*, octreotate, which targets somatostatin receptors in NET; or vipivotide tetraxetan (PSMA-617), which targets prostate-specific membrane antigen (PSMA) in mCRPC). However, pairing metal ions of different elements with different coordination chemistries in a theranostic approach requires the use of different chelators (*e.g.*, HBED-CC in ^68^Ga-gozetotide paired with DOTA in ^177^Lu-based Pluvicto™), which can result in different biodistribution properties between the imaging and therapy pair. Using different isotopes of the same element (*e.g.*, ^64^Cu for imaging, ^67^Cu for therapy) is one solution to this problem, since the requirement for a unique chelator (*e.g.*, sarcophagine)^8,9^ guarantees the properties of the imaging and therapy complexes are identical. Same-element imaging-therapy radiometal pairs, however, are few which limits opportunities to use mixed-element radiometal pairs with useful decay properties. Theranostic systems compatible with radiometals of different elements are being explored using discrete chelators with capacity for cross-metal binding.^10–14^

**Chart 1:**
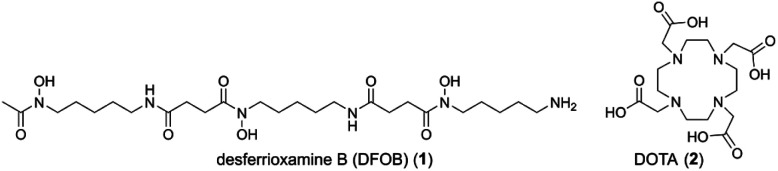
Structure of desferrioxamine B (DFOB, 1) and DOTA (2).

We have taken a different approach by grafting together two chelators, desferrioxamine B (DFOB, 1) and 1,4,7,10-tetraazacyclododecane-1,4,7,10-tetraacetic acid (DOTA, 2) (Chart 1), which have been established experimentally and/or clinically as useful chelators for ^89^Zr(iv) (positron-emission tomography (PET) imaging) or ^177^Lu(iii) (β-radiation therapy), respectively.

The risk of this approach is that most chelators have broad metal selectivity,^1–4^ forming complexes with a range of metal ions with variable affinities and stabilities, which could complicate robust verification and function of the radiolabelled product. The polyaminocarboxylic acid chelator 2 is a case in point, which can coordinate many radiometal ions, including but not limited to ^68^Ga, ^177^Lu, ^44^Sc, ^212^Pb, ^225^Ac and ^211^At.^1–4,15,16^ The hydroxamic acid chelator 1 can complex ^89^Zr, ^68^Ga, ^90^Nb, and others.^1–4,17–22^ Considering the metal selectivity of 1 and 2 as discrete compounds (Table 1) indicated the possibility that a covalent DFOB-DOTA adduct might have an unusual and useful regioselective metal binding function towards sets of imaging-therapy radiometal pairs of different elements, such as ^89^Zr (*t*_½_ = 3.3 d, *E*(*β*_max_^+^) = 901 keV (22.8%), EC = 77%, *E*_γ_ = 909 keV (100%))^18,23^ and ^177^Lu (*t*_½_ = 6.6 d, *E*(*β*_max_^−^) = 497 keV (78.6%), *E*_γ_ = 208 keV (11.1%), 113 keV (6.6%)).^24^

**Table 1:** Log *K* values of 1 : 1 complexes formed between DFOB (1) or DOTA (2) and selected metal ions with corresponding theranostic radioisotopes in use/development

Chelator	Log *K*				
	Zr(iv)	Lu(iii)	Ga(iii)	La(iii)	Pb(ii)
DFOB (1)	40.0^a^^38^	NR^b^	27.6^c^^39^	NR^b^	10.0^d^^40^
DOTA (2)	NR^b^	25.4^e^^41^	26.1^f^^42^	22.9^e^^41^	24.3^g^^43^

a 25 °C, I = 1 M NaClO_4_.

b NR = not reported.

c 25 °C, I = 1 M KCl.

d 25 °C, I = 0.1 M KCl.

e 25 °C, I = 0.1 M NaCl.

f 25 °C, I = 0.1 (CH_3_)_4_N^+^Cl^−^.

g 25 °C, I = 0.1 M NaClO_4_.

**Scheme 1:**
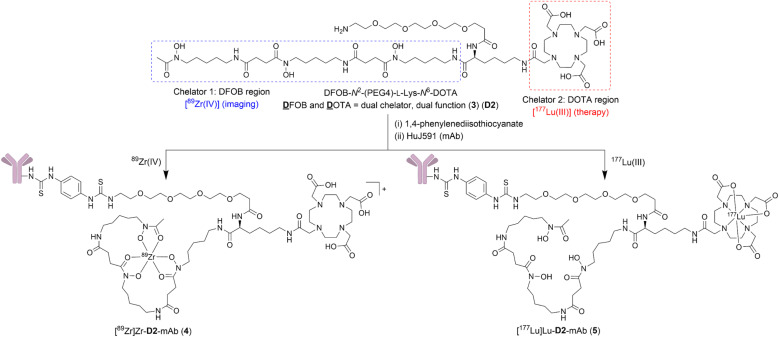
Structure of compound D2 (3), [^89^Zr]Zr-D2-mAb (4), and [^177^Lu]Lu-D2-mAb (5).

This work describes the preparation and characterisation of the dual chelator DFOB-*N*^2^-(PEG4)-l-Lys-*N*^6^-DOTA (3) (named ‘D2’ = D̲FOB and D̲OTA, Scheme 1). Compound 3 (D2) has been designed for regioselective binding of ^nat/89^Zr(iv) in the 1 region and ^nat/177^Lu(iii) in the 2 region, and includes an amine-terminated polyethylene glycol (PEG4) unit to enable further chemistry, including biomolecule conjugation. Radiolabelling D2-mAb (mAb = monoclonal antibody = HuJ591) with ^89^Zr(iv) or ^177^Lu(iii) gave [^89^Zr]Zr-D2-mAb (4) or [^177^Lu]Lu-D2-mAb (5) complexes, respectively (Scheme 1). The *in vitro* and *in vivo* properties of [^89^Zr]Zr-D2-mAb and [^177^Lu]Lu-D2-mAb were evaluated together with the matched single chelator control compounds [^89^Zr]Zr-1-mAb and [^177^Lu]Lu-2-mAb in LNCaP cell-based assays and a murine LNCaP prostate cancer xenograft model. [^89^Zr]Zr-D2-mAb and [^89^Zr]Zr-1-mAb showed similar PET/CT imaging function and tumour uptake (about 15% ID g^−1^) at 48 h and 120 h post injection, with SPECT/CT imaging showing tumour uptake of [^177^Lu]Lu-D2-mAb to support therapeutic function, which was corroborated by therapeutic efficacy data. The work identifies D2 as a trifunctional dual-chelator theranostic compound with potential for use with ^89^Zr for imaging or ^177^Lu for therapy, and other mixed-element radiometal pairs, to expand capabilities in nuclear medicine.

## Results and discussion

### Metal selectivity of discrete chelators DFOB and DOTA

#### DFOB (1)

Hydroxamic acids have a rich coordination chemistry.^25–27^ This includes the characterisation of many complexes between ^nat^Zr(iv) or ^89^Zr(iv) and 1 or higher denticity analogues designed to saturate the octadentate Zr(iv) coordination sphere to maximise the stability of ^89^Zr PET imaging agents.^19,28–37^ Despite the broad metal selectivity of hydroxamic acids, there are no reports of structurally characterised complexes between hydroxamic acids and Lu(iii).

**Fig. 1:**
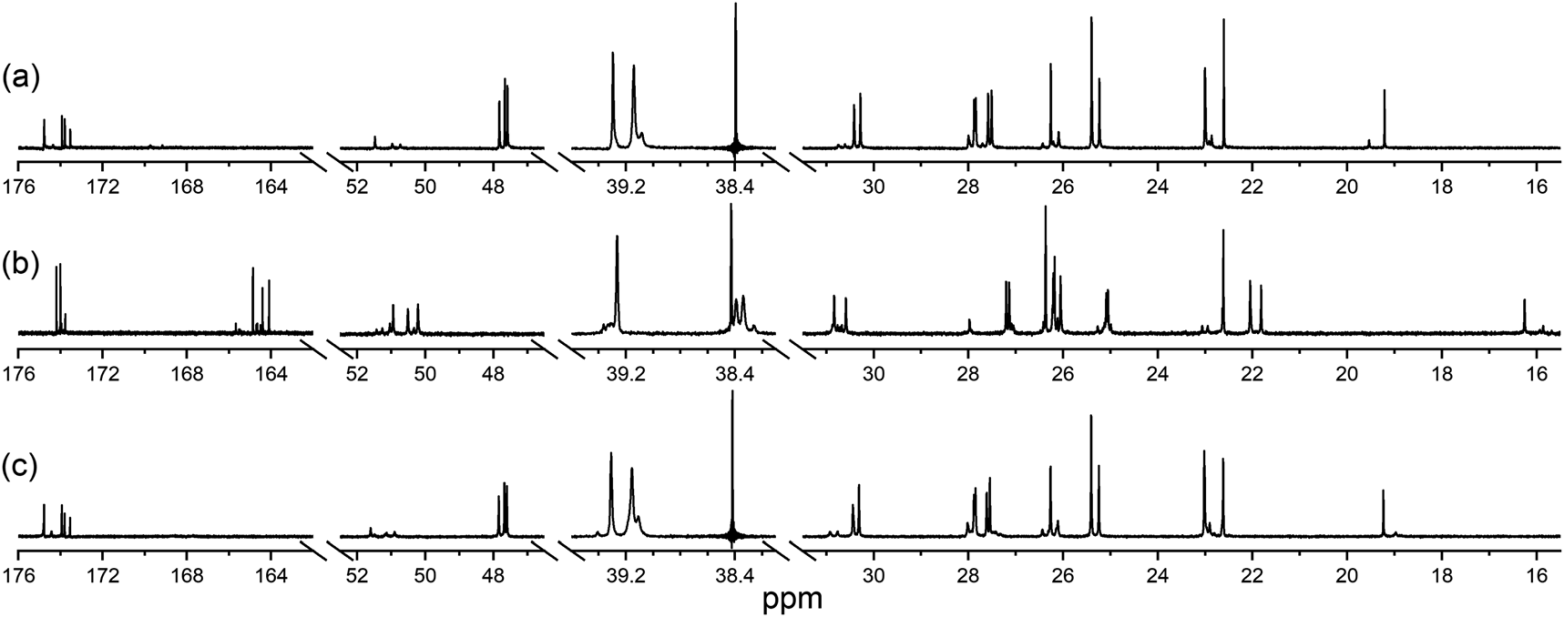
{^1^H}–^13^C NMR spectra from solutions (D_2_O) of (a) 1, (b) Zr(iv) : 1 (1.8 : 1), or (c) Lu(iii) : 1 (1.3 : 1). The signal at 38.4 ppm in (a) and (c) is due to a decoupler zero frequency spike.

The ability of 1 to bind Zr(iv) and not Lu(iii) was confirmed from {^1^H}–^13^C NMR spectroscopic measurements (Fig. 1) from D_2_O solutions of 1 alone, or in the presence of a stoichiometric excess of Zr(iv) or Lu(iii). The diamagnetism of Zr(iv) and Lu(iii) made this approach feasible with minimal metal-dependent line broadening.

Consistent with previous work on complexes between 1 and Al(iii) or Ga(iii),^39^ or Zr(iv),^44^ the cluster of signals between 173-175 ppm attributable to the C=O (amide) and C=O (hydroxamic acid) carbon atoms in the spectrum from 1 (Fig. 1a) were resolved in the presence of Zr(iv) (Fig. 1b) into two sets, with the C=O (hydroxamate) signals involved in metal coordination appearing upfield at 164.1–164.9 ppm. The two C=O signals in the Zr(iv)-1 complex at about 174 ppm that did not shift appreciably compared to 1 could be assigned as the two C=O (amide) carbon atoms, since these would experience less change in chemical environment. The signals at 47.6–47.8 ppm attributable to the methylene groups adjacent to the hydroxamic acid N–OH in free 1 moved downfield to 50.2–50.9 ppm in the presence of Zr(iv). The signal attributable to the terminal CH_3_ group in 1 shifted upfield from 19.2 to 16.3 ppm in the presence of Zr(iv), supporting Zr(iv)-1 complexation. In addition to these readily assigned signals, there were other spectral shifts in the Zr(iv)-1 system that demonstrated complex formation, which would likely include the presence of isomers.^39^

The spectra from free 1 or in solution with Lu(iii) (Fig. 1c) were close to coincident, indicating the absence of Lu(iii)-1 complexation. Metal complexation experiments were conducted at room temperature or at mildly elevated temperatures (≤37 °C) to mimic radiolabelling conditions and provide results relevant to procedures used for mAbs and other thermally labile biological vectors.

#### DOTA (2)

The complexation of Lu(iii) or ^177^Lu with 2 is well established, with 2 present in both Lutathera® and Pluvicto™. While a Zr(iv)-DOTA complex and Zr(iv) complexes with related polyaminocarboxylic acid chelators have been characterised^45,46^ the elevated temperature (≥65 °C) necessary for complexation with 2 suggests that the formation of Zr(iv)-DOTA species is less favourable at temperatures used for mAb radiolabelling (≤37 °C). The {^1^H}–^13^C NMR spectra from solutions of 2 alone or in the presence of Zr(iv) or Lu(iii) acquired at room temperature or −10 °C gave broad signals, as typically observed for 2 due to the presence of multiple interchanging conformational isomers.^47–49^ The compound DOTA-*N*^2^-acetyl-l-lysine methyl ester (8a) was prepared as a lower-symmetry 2 analogue and surrogate of the 2 region in D2. Spectra from solutions of Lu(iii) and 8a showed a set of broad signals between 55.5-66 ppm consistent with Lu(iii) coordination *via* the *endo*- and *exo*-methylene groups of the 2 motif. These signals were not evident in the Zr(iv) or free ligand system (Fig. S17†).

### Synthesis of DFOB-l-Lys-*N*^6^-DOTA (3a) and regioselective coordination of Zr(iv) and Lu(iii)

**Scheme 2:**
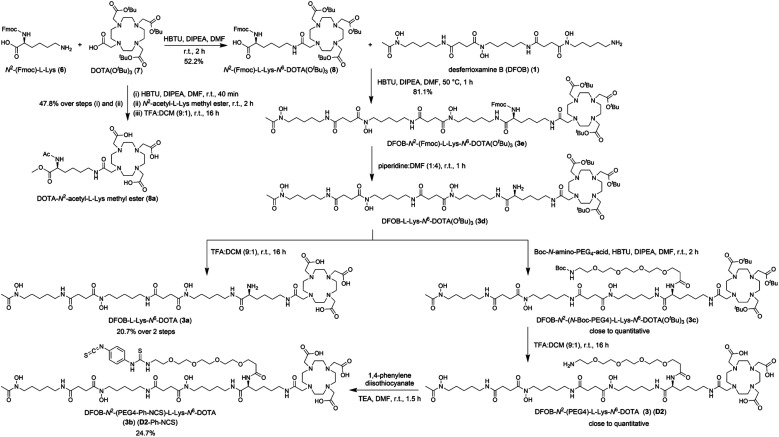
Synthesis of DFOB-*N*^2^-(PEG4)-l-Lys-*N*^6^-DOTA (3) (D2), DFOB-l-Lys-*N*^6^-DOTA (3a), and DFOB-*N*^2^-(PEG4-Ph-NCS)-l-Lys-*N*^6^-DOTA (3b) (D2-Ph-NCS).

The metal selectivity results from the {^1^H}–^13^C NMR spectroscopic data together with established Zr(iv)-1 and Lu(iii)-2 chemistry (Table 1) supported that under mild temperature conditions, a DFOB–DOTA covalent adduct would preferentially bind ^nat^Zr(iv) or ^89^Zr(iv) at the DFOB region and not at the DOTA region, and bind ^nat^Lu(iii) or ^177^Lu(iii) at the DOTA region and not at the DFOB region. A construct useful for targeted radiometal delivery requires a functional group to graft peptides and/or mAbs, which led to the design of DFOB-l-Lys-*N*^6^-DOTA (Scheme 2, 3a) as the preliminary target compound.

**Fig. 2:**
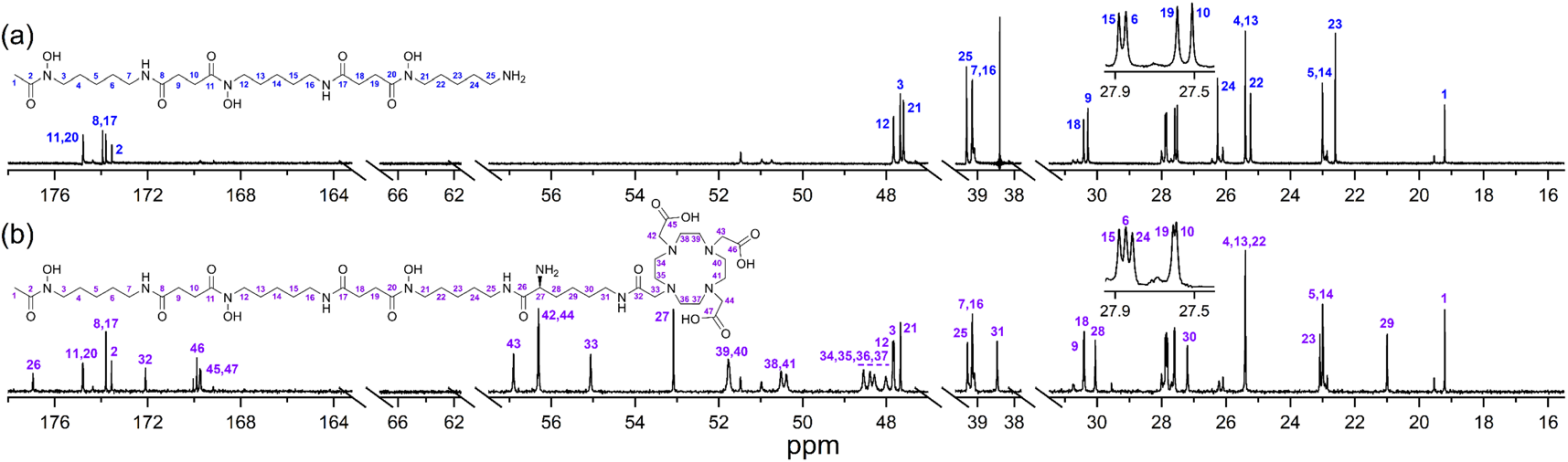
{^1^H}–^13^C NMR spectra from solutions (D_2_O) of (a) 1 or (b) 3a.

A protected precursor of 3a, DFOB-*N*^2^-(Fmoc)-l-Lys-*N*^6^-DOTA(O^*t*^Bu)_3_ (3e), was prepared from an HBTU-mediated amide coupling reaction between 1 and *N*^2^-(Fmoc)-l-Lys-*N*^6^-DOTA(O^*t*^Bu)_3_ (8), which itself was prepared from *N*^2^-(Fmoc)-l-Lys (6) and DOTA(O^*t*^Bu)_3_ (7). Global deprotection of 3e generated 3a in an overall yield of 21% (Scheme 2). The {^1^H}–^13^C NMR spectrum of 3a (Fig. 2b), assigned using data from the composite fragments 1 (Fig. 2a)^39^ and 8a, showed signals between 47.9–52 ppm and 54.8–57.1 ppm ascribed to the *endo*- and *exo*-methylene carbon atoms of the DOTA region, respectively. The carbonyl carbon atoms in the DOTA region of 3a were resolved at 169.6–169.8 ppm (C45, C47) and 169.9 ppm (C46). Compared to 2 or 8a, the nature and conformation of the extended structure present in 3a modulated the molecular tumbling to enable resolution of the signals ascribable to the DOTA region at room temperature.

**Fig. 3:**
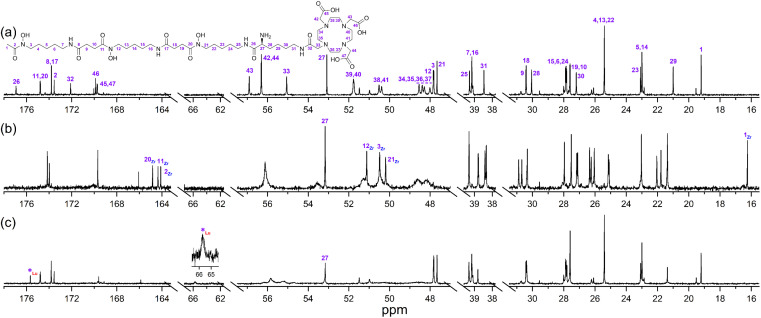
{^1^H}–^13^C NMR spectra from solutions (D_2_O) of (a) 3a, (b) Zr(iv) : 3a (0.8 : 1), or (c) Lu(iii) : 3a (0.8 : 1).

The {^1^H}–^13^C NMR spectra from 3a alone (Fig. 3a) or in the presence of Zr(iv) or Lu(iii) supported the regioselective coordination of Zr(iv) to the 1 region and Lu(iii) to the 2 region. The spectral changes in the Zr(iv)-3a system (Fig. 3b) were similar to those observed in the Zr(iv)-1 system (Fig. 1b), with an upfield shift in the three signals ascribed to the carbonyl carbon atoms of the Zr(iv) coordinating hydroxamic acid groups (164.1–164.8 ppm) and the terminal methyl group (16.3 ppm), and a downfield shift (50.1–51.3 ppm) in the three signals attributable to the methylene carbon atoms adjacent to the N–OH groups. These latter sharp signals were overlaid with the signals for the *endo*-methylene carbon atoms in the 2 region, and although broadened, their appearance supported the non-coordinating role of the 2 region in solutions of Zr(iv) and 3a.

Signals ascribed to the 1 region in the spectrum from a solution of 3a and Lu(iii) (Fig. 3c) mapped closely to the same ascribed signals in 3a alone, supporting the 1 region was non-coordinating towards Lu(iii). In the presence of Lu(iii), the signals for the *endo*- and *exo*-methylene carbon atoms of the 2 region in 3a were significantly broadened, with low intensity signals appearing at 63.3 ppm and 65.7 ppm (inset) and an upfield shift of a signal ascribed to one of the carbonyl carbon atoms in the 2 region in 3a. Together, these results supported that at room temperature, Zr(iv) preferentially coordinated the 1 region and not the 2 region in 3a, and Lu(iii) preferentially coordinated the 2 region and not the 1 region in 3a.

### Stoichiometry of complexes between Zr(iv), Lu(iii) or Ga(iii) and 3a

**Fig. 4:**
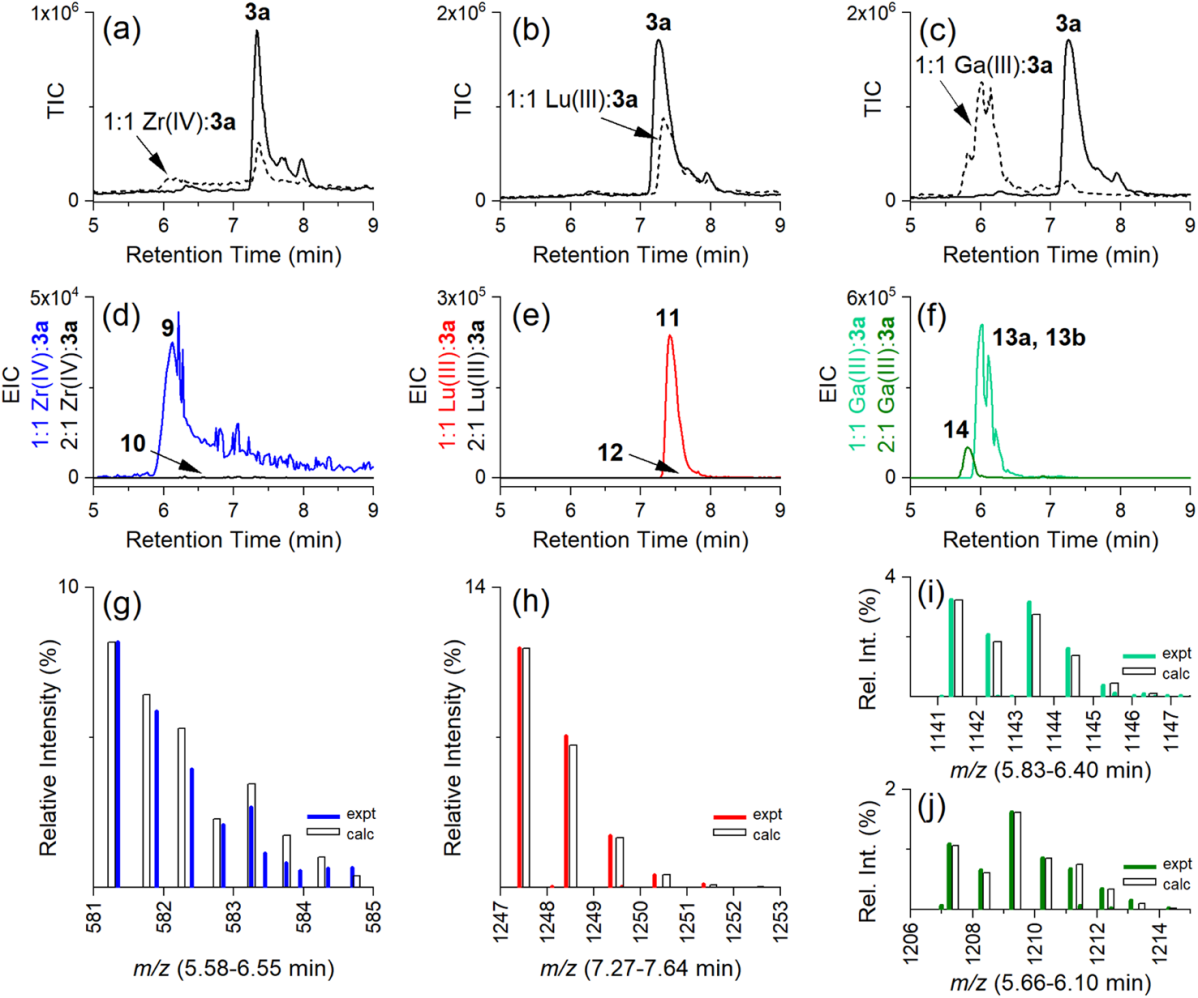
LC-MS traces (total ion current (TIC)) from solutions of 3a in the absence (a–c, solid line) or presence (broken line) of (a) Zr(iv), (b) Lu(iii), or (c) Ga(iii). EIC traces set to report protonated double-charged 1 : 1 or 2 : 1 metal : 3a complexes for (d) Zr(iv) (9) (blue) or (10) (black), (e) Lu(iii) (11) (red) or (12) (black), or (f) Ga(iii) (13a, 13b) (green) or (14) (olive), with the isotope pattern experimental (coloured line) or calculated (white column) for (g) 1 : 1 Zr(iv) : 3a (9) ({[M^+^ + H]}^2+^ adduct), (h) 1 : 1 Lu(iii) : 3a (11) ([M + H]^+^ adduct), (i) 1 : 1 Ga(iii) : 3a (13a, 13b) ([M + H]^+^ adduct), or (j) 2 : 1 Ga(iii) : 3a (14) ([M + H]^+^ adduct).

**Chart 2:**
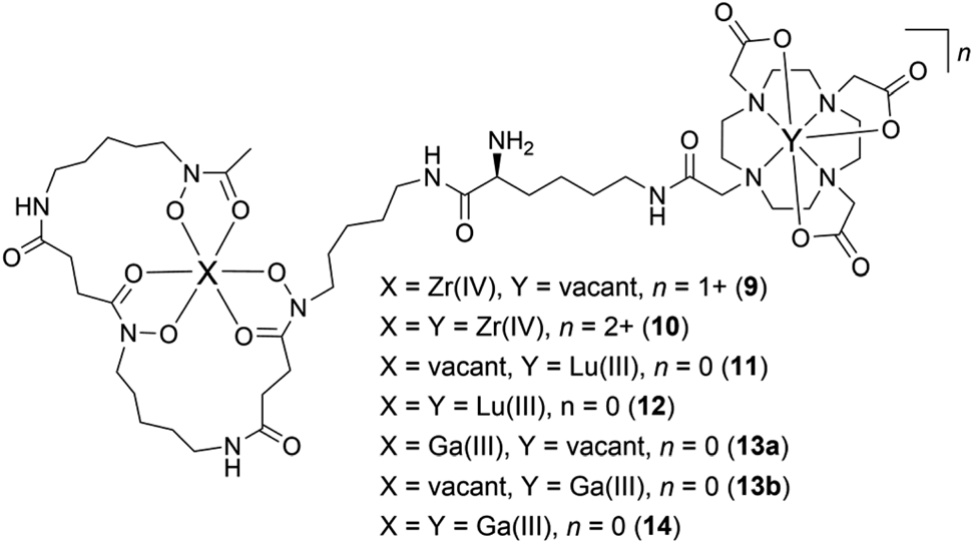
Complexes between Zr(iv), Lu(iii), or Ga(iii), and 3a, with a 1 : 1 (9, 11, 13a, 13b) or 2 : 1 (10, 12, 14) metal:3a stoichiometry.

Having established the regioselectivity of Zr(iv)/Lu(iii)-3a coordination from {^1^H}–^13^C NMR spectroscopy, complementary experiments to support selective binding were undertaken using liquid chromatography-mass spectrometry (LC-MS) to report metal:3a stoichiometry. In addition to Zr(iv) and Lu(iii), these experiments included Ga(iii), which has an affinity towards 1 and 2 (Table 1), and was useful in evaluating a potential case of cross-chelator coordination. Solutions of 3a were incubated with a molar equivalent (Fig. 4) of Zr(iv), Lu(iii) or Ga(iii) at 37 °C and analysed by LC-MS. The {^1^H}–^13^C NMR spectroscopic data predicted the formation of 1 : 1 Zr(iv):3a (Chart 2), [Zr(iv)-3a_(DFOB)_]^+^ (9), and not 2 : 1 Zr(iv):3a [(Zr(iv))_2_-3a_(DFOB)(DOTA)_]^2+^ (10). This expected trend in stoichiometry would be matched for Lu(iii), albeit with binding in the 2 region, with the formation of 1 : 1 Lu(iii) : 3a ([Lu(iii)-3a_(DOTA)_] (11)) and not 2 : 1 Lu(iv) : 3a ([(Lu(iii))_2_-3a_(DFOB)(DOTA)_] (12)). The affinity of Ga(iii) for 1 and 2 could result in the formation of two 1 : 1 Ga(iii):3a isomers indistinguishable by LC-MS ([Ga(iii)-3a_(DFOB)_] (13a) and [Ga(iii)-3a_(DOTA)_] (13b)), and the 2 : 1 Ga(iii) : 3a complex ([(Ga(iii))_2_-3a_(DFOB)(DOTA)_] (14)).

The intensity of the signal at 7.3 min in the LC-MS traces of the free ligand 3a (Fig. 4a–c, solid line) (total ion chromatogram (TIC)) was reduced in the presence of Zr(iv), Lu(iii), or almost completely in the presence of Ga(iii) (Fig. 4a–c, respectively, broken line), as consistent with the formation of complexes between Zr(iv), Lu(iii) or Ga(iii) and 3a.

Signals at *t*_R_ = 6.1 min or 7.4 min were detected from the extracted ion chromatogram (EIC) traces set to report 1 : 1 Zr(iv) : 3a (9) (Fig. 4d, blue) or Lu(iii) : 3a (11) (Fig. 4e, red), respectively (Table 2). No signals were observed for the 2 : 1 Zr(iv) : 3a (10) (Fig. 4d, black) or 2 : 1 Lu(iii) : 3a (12) (Fig. 4e, black) complexes. The experimental isotope patterns for 9 (Fig. 4g, blue) and 11 (Fig. 4h, red) were consistent with calculated patterns (white columns) in support of the assignment of these species.

**Table 2:** Metal : ligand complexes in 1 : 1 or 2 : 1 stoichiometries between Zr(iv), Lu(iii) or Ga(iii) and 3a (observed and theoretical)

	X^a^	Y^a^	*n*	Adduct (1+) *m*/*z*	Adduct (2+) *m*/*z*	Observed
9	Zr(iv)	Vacant	1+	1161.5^b^	581.3^c^	Y
10	Zr(iv)	Zr(iv)	2+	N/A	624.2^d^	N
11	Vacant	Lu(iii)	0	1247.6^e^	624.3^f^	Y
12	Lu(iii)	Lu(iii)	0	1419.5^e^	710.2^f^	N
13a	Ga(iii)	Vacant	0	1141.5^e^	571.3^f^	Y
13b	Vacant	Ga(iii)	0	1141.5^e^	571.3^f^	Y
14	Ga(iii)	Ga(iii)	0	1207.4^e^	604.2^f^	Y

a Refer Chart 2.

b [M]^+^.

c {([M]^+^ + H)}^2+^.

d [M]^2+^.

e [M + H]^+^.

f [M + 2H]^2+^.

The solution of Ga(iii) and 3a gave EIC signals at *t*_R_ = 6.0 min and 5.8 min correlating with complexes with 1 : 1 (13a, 13b) (Fig. 4f, green) and 2 : 1 (14) (Fig. 4f, olive) stoichiometries, respectively, showing that, unlike Zr(iv) and Lu(iii), Ga(iii) was able to simultaneously coordinate to the 1 and 2 regions of 3a. The assignment of the 1 : 1 isomer pair (13a, 13b) (Fig. 4i) and the 2 : 1 complex (14) (Fig. 4j) was supported by the match between the experimental and theoretical isotope patterns.

The selectivity of coordination of 3a as established with natural abundance ^nat^Zr(iv) and ^nat^Lu(iii) metal solutions supported progressing to radiolabelling studies and evaluating the potential of the D2 dual-chelator system to deliver molecularly targeted imaging/therapy radiation.

### Synthesis and characterisation of DFOB-*N*^2^-(PEG4)-l-Lys-*N*^6^-DOTA (3) (D2) and DFOB-*N*^2^-(PEG4-Ph-NCS)-l-Lys-*N*^6^-DOTA (3b) (D2-Ph-NCS)

A polyethylene glycol unit (PEG4) was appended to the internal l-Lys residue in 3a to increase water solubility and to increase the distance between the dual-chelator scaffold and the mAb to maintain mAb-antigen recognition properties. PEG units and simpler ether-containing constructs have been incorporated in other 1- and 2-type chelators to improve water solubility and other performance measures.^30,32,50–53^ Compound 3 (D2) (Scheme 2) was prepared from 3e by removing the Fmoc group to give 3d followed by an amide coupling reaction with *N*-Boc-amine-PEG4-acid to give 3c, and a final global deprotection step to give 3 (D2). A sample of 3 (D2) was reacted with 1,4-phenylenediisothiocyanate and the resultant 3b (D2-Ph-NCS) purified by HPLC and lyophilised. Other types of activating groups§§Examples of other activating groups are NHS-, maleimide-, or ethyl squaramide groups installed from reactions of D2 with bis-*N*-succinimidyl glutarate, *N*-succinimidyl-3-maleimidopropionate, or diethyl squarate, respectively. would be compatible with D2,^2,29,54,55^ with Ph-NCS selected here as one in common use, and useful for benchmarking performance against the discrete chelators DFOB-Ph-NCS (1-Ph-NCS) and DOTA-Bn-NCS (2-Bn-NCS). The overall yield of 3b (D2-Ph-NCS) starting from the reaction between 6 and 7 was approximately 10.4%. The most significant loss of yield occurred from the reaction between 3 (D2) and 1,4-phenylene diisothiocyanate, due in part to the poor solubility of the latter reagent in organic solvents.

### Antibody conjugation with D2-Ph-NCS (3b) and radiolabelling D2-mAb

The monoclonal antibody HuJ591 (mAb) was conjugated to D2-Ph-NCS (3b), 1-Ph-NCS or 2-Bn-NCS, and after buffer exchange, the resulting D2-mAb, 1-mAb or 2-mAb complexes were radiolabelled with ^89^Zr or ^177^Lu. The chelator : mAb ratio determined from MALDI data (Fig. S26†) was about 2 for 1-mAb, 3 for 2-mAb and 4 for D2-mAb (Table S1†). HuJ591 is a de-immunised variant of the antibody J591 that targets PSMA expressed on LNCaP cells, which is a cell line established from metastatic prostate cancer.^56–61^

**Fig. 5:**
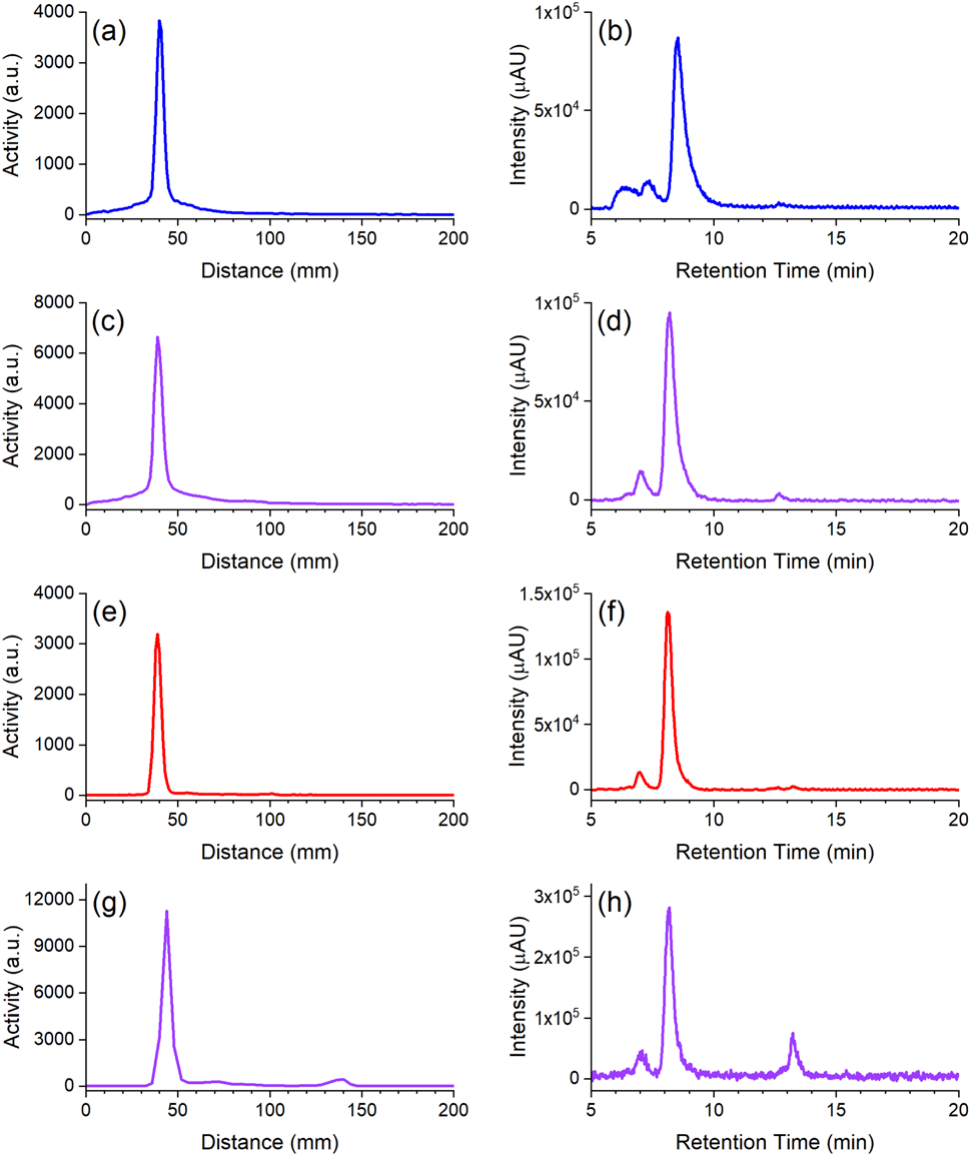
Radio-iTLC (left column) or radio-SEC-HPLC (right column) traces from 1-mAb (a and b) or D2-mAb (c and d) radiolabelled with ^89^Zr, and 2-mAb (e and f) or D2-mAb (g and h) radiolabelled with ^177^Lu. Radio-iTLC reaction mixtures were mixed with 1 : 1 5 mM EDTA (^177^Lu) or DTPA (^89^Zr) and developed in a 1 : 1 EtOH : H_2_O solvent system (Agilent iTLC-SG Glass microfiber chromatography paper impregnated with silica gel).

The radio-iTLC traces from D2-mAb radiolabelled with ^89^Zr or ^177^Lu gave well-resolved signals characteristic of [^89^Zr]Zr-D2-mAb (Fig. 5c) or [^177^Lu]Lu-D2-mAb (Fig. 5g) and correlated with the respective signals for [^89^Zr]Zr-1-mAb (Fig. 5a) or [^177^Lu]Lu-2-mAb (Fig. 5e) as matched controls. An earlier radiolabelling study using D2 conjugated to the mAb girentuximab showed similar results with D2-mAb and showed minimal (<1%) radiolabelling of 2 with ^89^Zr and no detectable radiolabelling of 1 with ^177^Lu at mild temperatures (Fig. S25†), in accord with the chelator-metal selectivity observed using ^nat^Zr(iv) and ^nat^Lu(iii).

### *In vitro* properties and radiochemical stability of [^89^Zr]Zr-D2-mAb (4) and [^177^Lu]Lu-D2-mAb (5)

The *in vitro* cell membrane binding and internalisation of [^89^Zr]Zr-D2-mAb, [^177^Lu]Lu-D2-mAb, and the single-chelator controls [^89^Zr]Zr-1-mAb and [^177^Lu]Lu-2-mAb, were assessed in LNCaP prostate cancer cells, in which the binding specificity of HuJ591 and cell-surface PSMA is well established.^58,59,62^

**Fig. 6:**
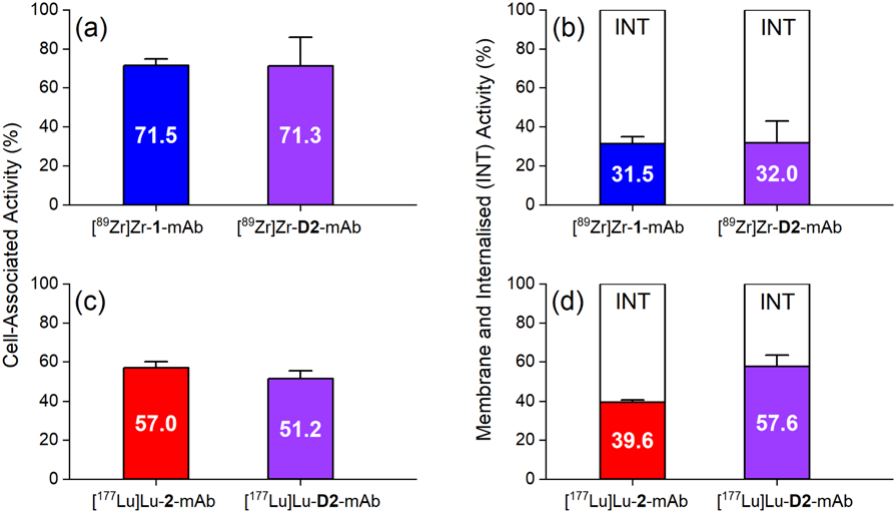
Average percentage of cell-associated activity (LNCaP cells) at 1 h of (a) [^89^Zr]Zr-1-mAb (blue), [^89^Zr]Zr-D2-mAb (purple), and (c) [^177^Lu]Lu-2-mAb (red), [^177^Lu]Lu-D2-mAb (purple); and membrane (coloured) or internalised (white) fractions of (b) [^89^Zr]Zr-1-mAb (blue), [^89^Zr]Zr-D2-mAb (purple), and (d) [^177^Lu]Lu-2-mAb (red), [^177^Lu]Lu-D2-mAb (purple).

*In vitro* cell-associated activity at 1 h was similar between [^89^Zr]Zr-D2-mAb and [^89^Zr]Zr-1-mAb (Fig. 6a), and between [^177^Lu]Lu-D2-mAb and [^177^Lu]Lu-2-mAb (Fig. 6c), showing the mAb was functional in D2-mAb. Levels of cell-membrane and internalised activity were similar between [^89^Zr]Zr-D2-mAb and [^89^Zr]Zr-1-mAb (Fig. 6b), with a slight reduction in internalisation of [^177^Lu]Lu-D2-mAb compared to [^177^Lu]Lu-2-mAb (Fig. 6d). The capacity of radiolabelled antibodies to accumulate and remain intracellularly (residualisation) can be useful in maximising DNA radiation damage in endo-radiotherapy.^63,64^ The radiochemical stability of [^89^Zr]Zr-D2-mAb and [^89^Zr]Zr-1-mAb was measured using radio-iTLC and radio-SEC-HPLC in human serum and in phosphate-buffered saline (Fig. S27–S30 and Table S3†), with sampling at day 0, 2, 4 and 7. [^89^Zr]Zr-D2-mAb maintained radiochemical purity in human serum at day 7 to 81% (radio-SEC-HPLC) or 88% (radio-iTLC) with the respective values for [^89^Zr]Zr-1-mAb at 61% or 76%.

### *In vivo* properties of [^89^Zr]Zr-D2-mAb (4) and [^177^Lu]Lu-D2-mAb (5)

**Fig. 7:**
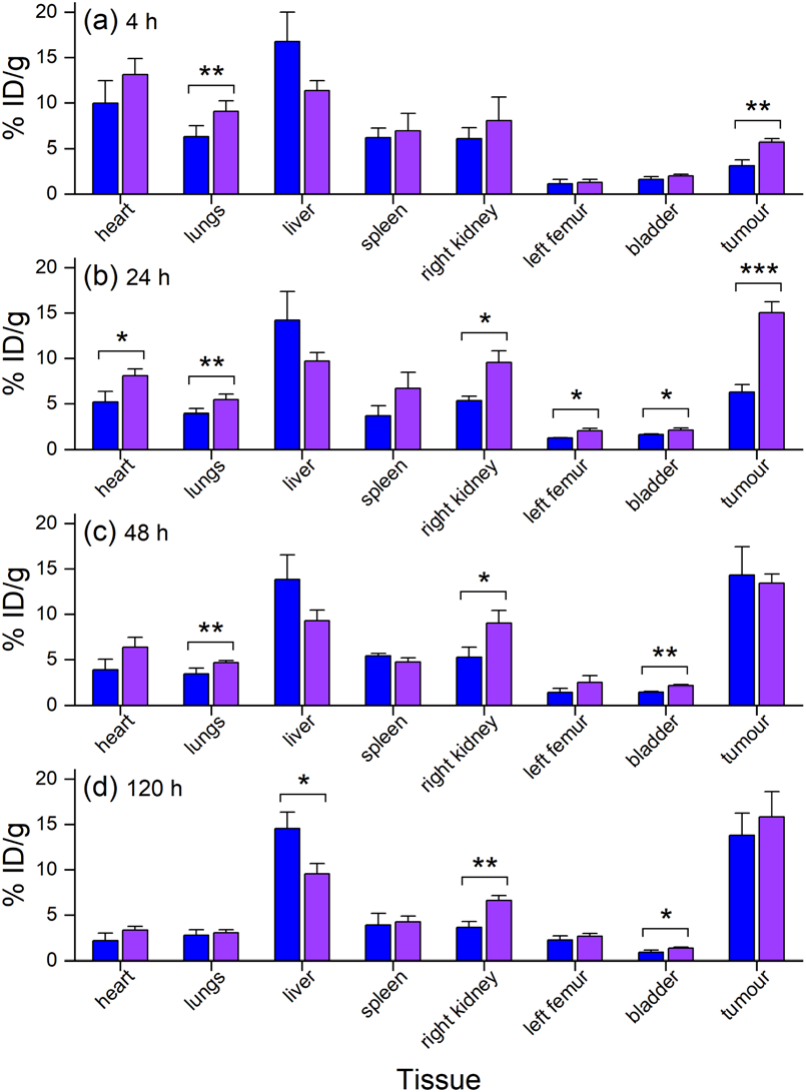
*In vivo* biodistribution (*n* = 3) of [^89^Zr]Zr-1-mAb (blue) and [^89^Zr]Zr-D2-mAb (purple) (*n* = 3) at (a) 4 h, (b) 24 h, (c) 48 h, or (d) 120 h post-injection as determined by ROI analysis of PET/CT images. *p ≤ 0.05, **p ≤ 0.01, ***p ≤ 0.001.

The biodistribution in tumour and other tissues of [^89^Zr]Zr-D2-mAb, [^177^Lu]Lu-D2-mAb, and the single-chelator controls [^89^Zr]Zr-1-mAb and [^177^Lu]Lu-2-mAb, were assessed *in vivo* in a murine LNCaP xenograft model by PET/CT imaging and SPECT/CT imaging or *ex vivo* by gamma counting.¶¶All studies involving animals were conducted in accordance with the guidelines of the Animal Ethics Committee of The University of Queensland, and the Australian Code for the Care and Use of Animals for Scientific Purposes (AEC approval number: 2022/AE000135). [^89^Zr]Zr-D2-mAb and [^89^Zr]Zr-1-mAb showed similar tumour/tissue biodistribution at 4 h post-injection (Fig. 7a), with [^89^Zr]Zr-D2-mAb showing significantly higher accumulation in the tumour. This trend continued at 24 h post-injection (Fig. 7b).

From 48 h to 120 h post-injection, the level of accumulation in the tumour showed no difference between [^89^Zr]Zr-D2-mAb and [^89^Zr]Zr-1-mAb (Fig. 7c and d). At 120 h post-injection, there was no statistical difference in bone uptake between [^89^Zr]Zr-D2-mAb and [^89^Zr]Zr-1-mAb, suggesting the stability of ^89^Zr binding was similar between the two conjugates (Fig. 7d).

**Fig. 8:**
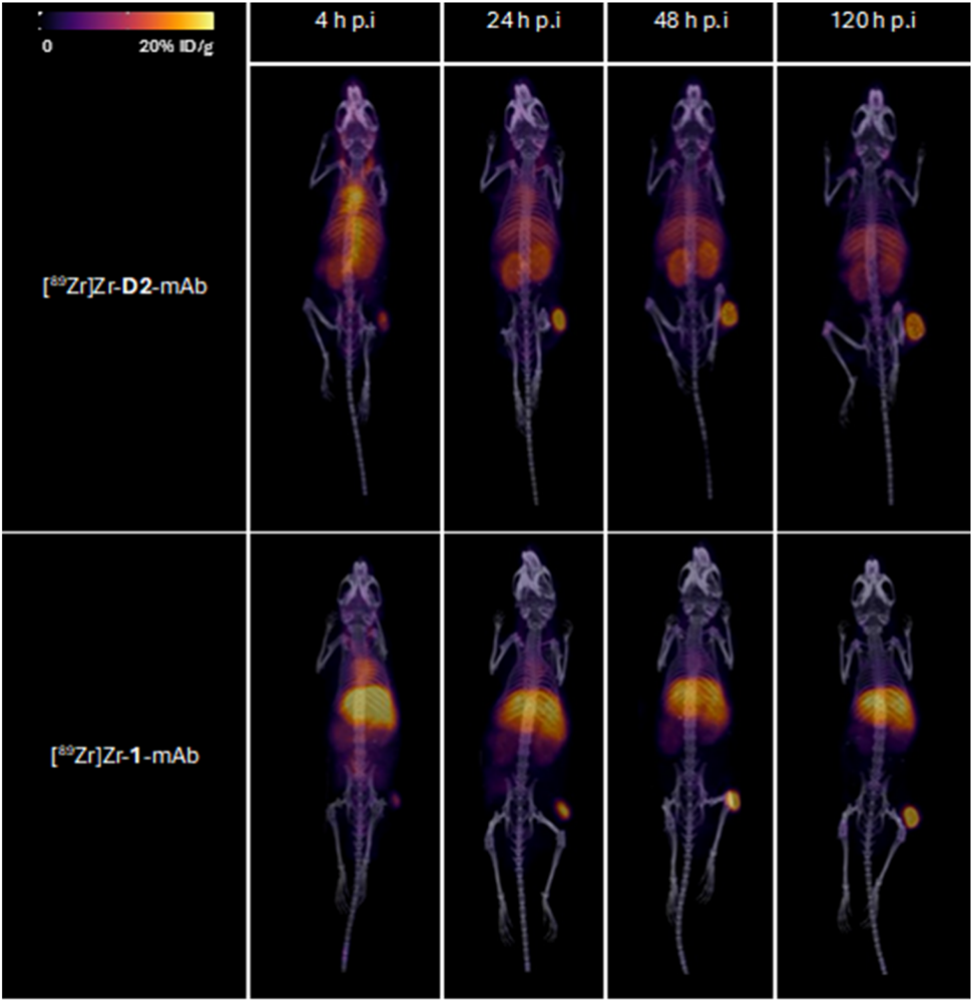
Representative 3D maximum intensity projections for all mice ([^89^Zr]Zr-D2-mAb upper; [^89^Zr]Zr-1-mAb, lower); in ^89^Zr PET/CT imaging cohort at 4, 24, 48 and 120 h post-injection. Signal normalised to same thresholding (% ID g^−1^).

**Fig. 9:**
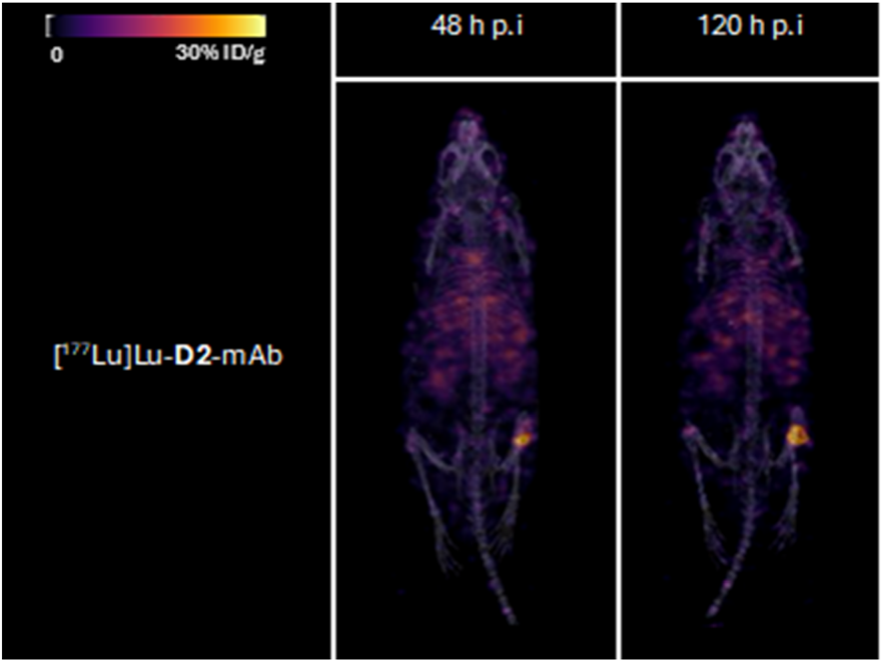
Representative 3D maximum intensity projections for all mice ([^177^Lu]Lu-D2-mAb) in ^177^Lu SPECT/CT imaging cohort at 48 and 120 h post-injection. Signal normalised to same thresholding (% ID g^−1^).

Tumour xenografted mice imaged by PET/CT using [^89^Zr]Zr-D2-mAb (upper) or [^89^Zr]Zr-1-mAb (lower) (Fig. 8) or by SPECT/CT using [^177^Lu]Lu-D2-mAb (Fig. 9) showed a discernible tumour at all timepoints, with signals isolated from background and able to be delineated without interference.

**Fig. 10:**
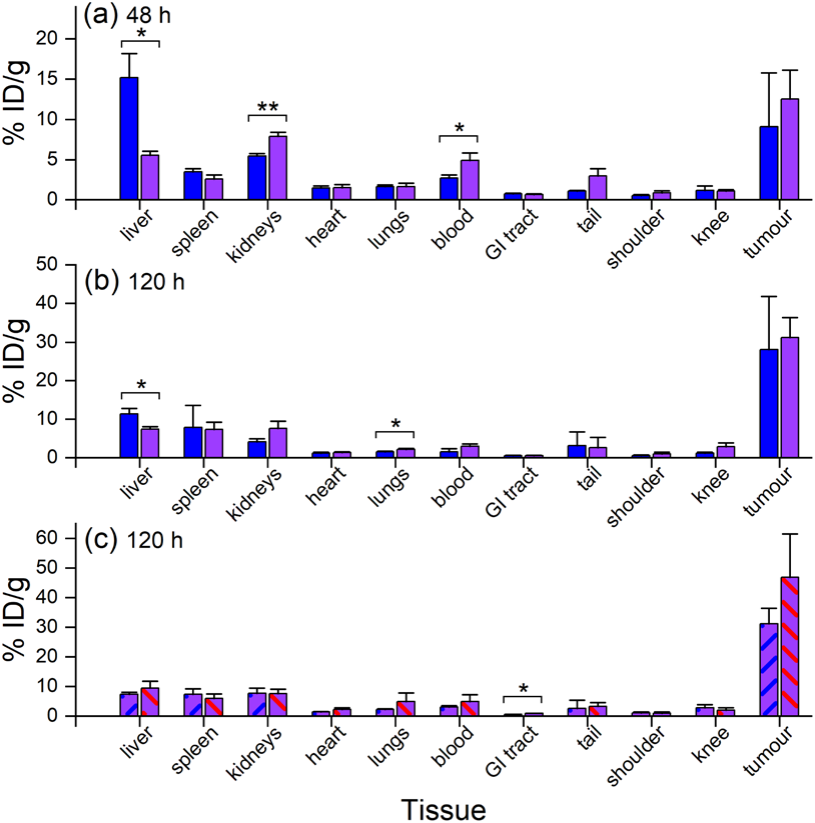
*Ex vivo* biodistribution from gamma counting (*n* = 3) of [^89^Zr]Zr-1-mAb (blue) and [^89^Zr]Zr-D2-mAb (purple) at (a) 48 h p. i. or (b) 120 h p. i., or (c) [^89^Zr]Zr-D2-mAb (purple, blue diagonal line) and [^177^Lu]Lu-D2-mAb (purple, red diagonal line) at 120 h p. i. *p ≤ 0.05, **p ≤ 0.01.

*Ex vivo* measurements of [^89^Zr]Zr-D2-mAb or [^89^Zr]Zr-1-mAb correlated similarly to *in vivo* biodistribution and the PET/CT images at 48 h (Fig. 7c, 8 and 10a) and 120 h (Fig. 7d, 8 and 10b) post-injection. There was a significant difference in *ex vivo* biodistribution in the kidney and liver tissues at 48 h post-injection (Fig. 10a), with these differences reducing at 120 h post-injection (Fig. 10b). Most other collected tissues showed no significant difference in biodistribution between [^89^Zr]Zr-D2-mAb or [^89^Zr]Zr-1-mAb, with trends similar to *in vivo* biodistribution at the same time points (Fig. 7c, d; 10a and b).

*Ex vivo* biodistribution of [^89^Zr]Zr-D2-mAb and [^177^Lu]Lu-D2-mAb showed a significant difference in accumulation only within the gastrointestinal tract tissue, with all other tissues showing similar biodistribution (Fig. 10c). Both [^89^Zr]Zr-D2-mAb and [^177^Lu]Lu-D2-mAb showed minimal uptake into bone tissue (shoulder and knee) at 120 h post-injection, which correlated to the minimal uptake observed in the *in vivo* biodistribution (Fig. 8 and 9). This suggests that the [^89^Zr]Zr-D2-mAb complex remained stable up to 120 h *in vivo*, with bone tissue signals a typical marker of complex instability arising from the osteophilic nature of ^89^Zr(iv).^35,65^ The similar biodistribution properties of [^89^Zr]Zr-D2-mAb and [^177^Lu]Lu-D2-mAb indicate D2 could have potential utility for improved imaging-therapy dosimetry. The biodistribution of [^89^Zr]Zr-D2-mAb and [^89^Zr]Zr-1-mAb, and [^177^Lu]Lu-D2-mAb and [^177^Lu]Lu-2-mAb, supported that D2-mAb formulated for either imaging or therapy could function similar to the matched single-chelator controls.^66^

#### Therapeutic efficacy of [^177^Lu]Lu-D2-mAb (5) and [^177^Lu]Lu-2-mAb

**Fig. 11:**
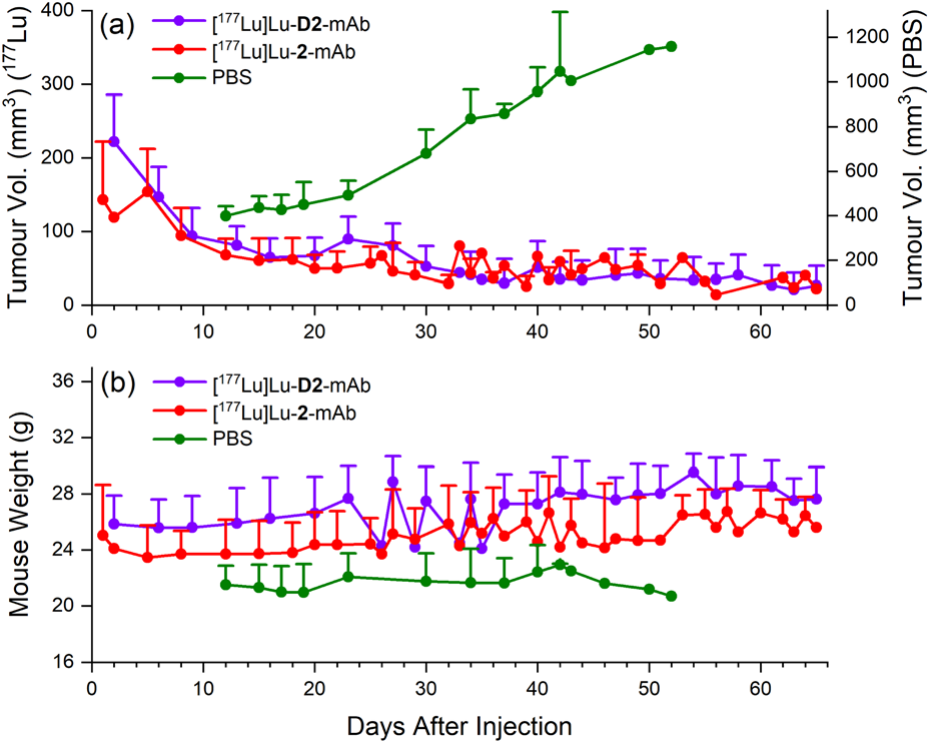
Therapeutic efficacy of [^177^Lu]Lu-D2-mAb (purple) and [^177^Lu]Lu-2-mAb (red) in a murine LNCaP xenograft model, monitored over a 65 day period, with vehicle only PBS as control (green). Therapeutic efficacy in each cohort was measured by changes in tumour volume (a) and weight (b).

Differences in tumour volume and weight in a murine LNCaP xenograft model were monitored over a 65 day period to assess therapeutic efficacy of [^177^Lu]Lu-D2-mAb in comparison to [^177^Lu]Lu-2-mAb, using phosphate-buffered saline (PBS) as a vehicle only control (Fig. 11).

Therapeutic efficacy of [^177^Lu]Lu-D2-mAb was found to closely match that of [^177^Lu]Lu-2-mAb, with both compounds showing >80% reduction in tumour volume (<40 mm^3^ after 65 days, Fig. 11a) and a slight increase in weight over the 65 day monitoring period (Fig. 11b). Both treatment cohorts exhibited a sustained reduction in tumour volume and sustained increase in weight over the observation period. The vehicle only cohort displayed an increase in tumour volume until ethical cut-offs were reached and mice were euthanised in accordance with the ethics approval guidelines (Fig. 11a). A trend of increasing weight loss was exhibited in the vehicle only cohort (Fig. 11b). These efficacy data showed the therapeutic potential of [^177^Lu]Lu-D2-mAb, with similar performance to [^177^Lu]Lu-2-mAb.

## Conclusions

A covalent adduct between desferrioxamine B (DFOB, 1) and 1,4,7,10-tetraazacyclododecane-1,4,7,10-tetraacetic acid (DOTA, 2) separated by an l-lysine unit (DFOB-l-Lys-*N*^6^-DOTA, 3a) and containing an amine-terminated PEG motif for further chemistry and biomolecule conjugation was prepared and named ‘D2’ (3). The dual chelator D2-mAb complex was selectively radiolabelled with ^89^Zr or ^177^Lu to form [^89^Zr]Zr-D2-mAb (4) or [^177^Lu]Lu-D2-mAb (5), respectively, with the regioselectivity of ^89^Zr(iv) binding to the 1 region or ^177^Lu(iii) to the 2 region, consistent with {^1^H}–^13^C NMR spectroscopic data, and complementary LC-MS measurements supporting the 1 : 1 metal : ligand stoichiometry. [^89^Zr]Zr-D2-mAb and [^89^Zr]Zr-1-mAb showed similar PET/CT imaging function and [^177^Lu]Lu-D2-mAb and [^177^Lu]Lu-2-mAb showed similar therapeutic efficacy in a murine LNCaP prostate cancer xenograft model, with tumour localisation of [^177^Lu]Lu-D2-mAb shown by SPECT/CT imaging.

The functional properties of [^89^Zr]Zr-D2-mAb and [^177^Lu]Lu-D2-mAb demonstrates the potential of D2 as a single ^89^Zr–^177^Lu theranostic agent which could streamline development pathways and improve patient dosimetry. D2 could also open opportunities to use other mixed-element radiometal pairs, including ^89^Zr (imaging) and ^225^Ac (therapy) or ^212^Pb (therapy), which forms ongoing work, together with studies to examine properties of D2 co-labelled with ^89^Zr and ^nat^Lu (imaging) or with ^177^Lu and ^nat^Zr (therapy), with this iso-structural and iso-charge imaging-therapy pair predicted to have identical biodistribution properties.

## Experimental

### Materials and methods

*O*-(1*H*-Benzotriazol-1-yl)-*N*,*N*,*N*′,*N*′-tetramethyluronium hexafluorophosphate (HBTU, 98%) was obtained from Alfa Aesar. 2,2-Dimethyl-4-oxo-3,8,11,14,17-pentaoxa-5-azaicosan-20-oic acid (*N*-Boc-PEG4-CO_2_H, 97%) was obtained from AmBeed. Ammonium acetate (97%) was obtained from APS Finechem. Sodium chloride (99.7%) was obtained from Chemsupply. HEPES (99.7%) was obtained from Formedium. DOTA-tri(^*t*^Bu) ester (≥95%), DFOB-Ph-NCS (≥94%) and DOTA-Bn-NCS (≥94%) were obtained from Macrocyclics. Dichloromethane (≥99.9%), *N*-ethyldiisopropylamine (≥98%), sodium hydrogen carbonate (99.7%), formic acid (98–100%) and magnesium sulfate, anhydrous (≥98%) were obtained from Merck. Acetonitrile (99.9%) was obtained from RCI Labscan. Hydrochloric acid (36%) and toluene (99.5%) were obtained from Ajax Finechem. Desferrioxamine mesylate (≥92.5%), *N*,*N*-dimethylformamide, anhydrous (99.8%), piperidine (99%), sodium hydroxide, gallium(iii) acetylacetonate (99.99%), lutetium(iii) chloride (99.99%), zirconium(iv) chloride (≥99.9%), sodium hydroxide (≥98%), methanol, anhydrous (99.8%), triethylamine (≥99%), *p*-phenylene diisothiocyanate (98%), diethyl ether, anhydrous (≥99.7%) and trifluoroacetic acid (99%) were obtained from Sigma-Aldrich. *N*_α_-[(9*H*-Fluoren-9-ylmethoxy)carbonyl]-l-lysine hydrochloride (>98%) was obtained from TCI. Milli-Q water was prepared using a Millipore Q-pod system and used as required. Healthy male Balb/c nude mice from 8 weeks old (weight ∼20 g) were obtained from Ozgene. Human serum used for stability studies was obtained from Sigma-Aldrich (product number H4522). Human LnCaP clone FGC cells were obtained from the American Type Culture Collection (ATCC, product number CRL-1740) and used for xenograft tumour growth and cell assays.

### Synthesis and characterisation

#### *N* ^2^-(Fmoc)-l-Lys-*N*^6^-DOTA(O^*t*^Bu)_3_ (8)

To a solution of DOTA(O^*t*^Bu)_3_ (7) (1.02 g, 1.78 mmol) in DMF (50 mL) was added DIPEA (2.07 mL, 11.9 mmol). The mixture was stirred at r. t for 10 min before the addition of HBTU (652.2 mg, 1.72 mmol). After a further 30 min period of stirring, *N*^2^-(Fmoc)-l-lysine HCl (6) (707.2 mg, 1.92 mmol) was added, and the mixture was stirred for 2 h at r. t. before removing the solvent *in vacuo*. The residue was purified *via* solid phase extraction and the collected fractions were combined and the solvent was removed *in vacuo* to yield 8 (862.2 mg, 0.93 mmol, 52.2%) as a yellow-white solid. LRMS (ESI) *m*/*z*: [M + H]^+^ calculated for C_49_H_75_N_6_O_11_^+^ 923.55, found 923.35; [M + 2H]^2+^ calculated for C_49_H_76_N_6_O_11_^2+^ 462.28, found 462.35.

#### DFOB-*N*^2^-(Fmoc)-l-Lys-*N*^6^-DOTA(O^*t*^Bu)_3_ (3e)

To a solution of 8 (485.0 mg, 0.53 mmol) in DMF (10 mL) was added DIPEA (158.0 μL, 0.91 mmol). The mixture was left to stir at r. t for 10 min before the addition of HBTU (202.2 mg, 0.53 mmol). This mixture was stirred at r. t for a further 30 min before the addition of desferrioxamine mesylate (1) (358.2 mg, 0.55 mmol), with the solution stirred at 50 °C for 1 h and the solvent removed *in vacuo*. The residue was dissolved in DCM (200 mL) and washed with saturated sodium bicarbonate (3 × 100 mL) and saturated brine (100 mL) before drying the organic layer with anhydrous magnesium sulphate. The organic layer was filtered, and the solvent was removed *in vacuo* to yield 3e as a yellow oil (623.5 mg, 0.43 mmol, 81.1%). LRMS (ESI) *m*/*z*: [M + H]^+^ calculated for C_74_H_121_N_12_O_18_^+^: 1465.89, found 1465.90; [M + 2H]^2+^ calculated: 733.45, found 733.70; [M + 3H]^3+^ calculated: 489.30, found 489.50.

#### DFOB-l-Lys-*N*^6^-DOTA(O^*t*^Bu)_3_ (3d)

Compound 3e (623.5 mg, 0.43 mmol) was dissolved in a solution of DMF : piperidine (4 mL : 1 mL) and the solution was stirred at r. t for 1 h, and the solvent removed *in vacuo*. Cold ether (approx. 10 mL) was added to the residue and after stirring for 15 min at 0 °C, the ether was decanted, and the residue dried *in vacuo* to yield 3d as a pale-yellow oil. LRMS (ESI) *m*/*z*: [M + H]^+^ calculated for C_59_H_111_N_12_O_16_^+^: 1243.83, found 1243.60; [M + 2H]^2+^ calculated: 622.42, found 622.50; [M + 3H]^3+^ calculated: 415.28, found 415.30. This intermediate was used in subsequent steps without further purification.

#### DFOB-l-Lys-*N*^6^-DOTA (3a)

The sample of 3d was dissolved in a solution of TFA : DCM (4.5 mL : 0.5 mL) and left to stir for 16 h at r. t before removing the solvent *in vacuo*. The residue was resuspended in toluene (10 mL) and the solvent was removed *in vacuo*. The residue was further purified *via* HPLC and lyophilised to yield 3a (95.4 mg, 88.8 μmol, 20.7%) as a white powder. ^13^C NMR (600 MHz, D_2_O) *δ* 176.93, 174.79, 174.77, 173.80 (2C), 173.55, 172.09, 169.89, 169.75, 169.72, 56.90, 56.31 (2C), 55.06, 53.08, 51.78 (2C), 50.52, 50.39, 48.54, 48.39, 48.29, 48.01, 47.85, 47.82, 47.66, 39.27, 39.15, 39.08, 38.46, 30.42, 30.40, 30.06, 27.88, 27.85, 27.81, 27.61, 27.59, 27.20, 25.40 (3C), 23.08, 22.99 (2C), 21.00, 19.21. HRMS (H-ESI II) *m*/*z*: [M + H]^+^ calculated for C_47_H_87_N_12_O_16_^+^: 1075.63575, found 1075.63498; [M + 2H]^2+^ calculated: 538.321514, found 538.320867; [M + 3H]^3+^ calculated: 359.216768, found 359.216712.

#### DFOB-*N*^2^-(*N*-Boc-PEG4)-l-Lys-*N*^6^-DOTA(O^*t*^Bu)_3_ (3c)

To a solution of 3d (532.9 mg, 0.43 mmol) in DMF was added DIPEA (124.5 μL, 0.71 mmol). The mixture was stirred at r. t for 10 min before the addition of HBTU (163.4 mg, 0.43 mmol). This mixture was stirred at r. t for a further 30 min before the addition of *N*-Boc-PEG4-CO_2_H (approx. 146 mg, 0.40 mmol). The mixture was stirred for 2 h at r. t. and the solvent was removed *in vacuo*. The residue was dissolved in DCM (200 mL) and washed with saturated sodium bicarbonate (3 × 100 mL) and saturated brine (100 mL) before drying the organic layer with anhydrous magnesium sulphate. The organic layer was filtered, and the solvent was removed *in vacuo* to yield 3c (574.9 mg, 0.40 mmol, 100%) as a yellow oil. LRMS (ESI) *m*/*z*: [M + 2H]^2+^ calculated for C_75_H_141_N_13_O_23_^2+^: 796.0, found 796.0; [M + 3H]^3+^ calculated: 497.65, found 497.80. *N*-Boc fragmentation was typically observed during LC-MS analysis.

#### DFOB-*N*^2^-(PEG4)-l-Lys-*N*^6^-DOTA (3) (D2)

The sample of 3c was dissolved in a mixture of TFA : DCM (9 : 1) which was stirred at r. t for 16 h and the solvent was removed *in vacuo*. The residue was resuspended in toluene (10 mL) and the solvent was removed *in vacuo*. The residue was purified *via* solid phase extraction (100% ACN for 4 min, 100% H_2_O for 2.4 min, load, 100% ACN for 2 min, 20–80% ACN : H_2_O in 5% steps at 3 min each step, 95% ACN : H_2_O for 3 min, 10 mL min^−1^), and the collected fractions were combined, and the solvent was removed *in vacuo*. A fraction of the semi-pure product was further purified *via* HPLC and lyophilised to yield D2 (3) as a white-brown solid (3.0 mg, 2.27 μmol). ^1^H NMR (600 MHz, D_2_O) *δ* 8.42 (s, 0H), 8.42 (s, 0H), 4.01 (t, *J* = 6.7 Hz, 1H), 3.82 (qd, *J* = 16.8, 7.8 Hz, 5H), 3.64 (d, *J* = 10.3 Hz, 5H), 3.61 (d, *J* = 6.8 Hz, 5H), 3.48–3.41 (m, 14H), 3.18 (q, *J* = 8.7, 6.8 Hz, 10H), 3.11 (s, 7H), 3.01 (d, *J* = 15.5 Hz, 3H), 2.91 (d, *J* = 15.3 Hz, 3H), 2.80 (t, *J* = 7.1 Hz, 5H), 2.50 (t, *J* = 7.1 Hz, 5H), 2.15–2.10 (m, 4H), 1.90 (q, *J* = 7.0 Hz, 3H), 1.66–1.41 (m, 21H), 1.31 (t, *J* = 7.8 Hz, 8H). ^13^C NMR (600 MHz, D_2_O) *δ* 172.39, 172.34 (3C), 172.10 (2C), 171.88, 171.84, 170.6 (2C), 170.3, 70.22 (3C), 70.18, 70.11, 70.05, 69.97, 67.28, 58.92 (2C), 56.29, 55.93 (3C), 53.16, 51.55 51.22, 50.3 (2C), 47.54 (2C), 47.24 (3C), 38.99, 38.90 (2C), 39.77, 38.71, 36.30, 30.49, 30.43, 29.28 (3C), 29.09 (3C), 29.02, 28.13, 28.09, 23.97 (2C), 23.90, 23.21, 20.82 (2C). LRMS (ESI) *m*/*z*: [M + 2H]^2+^ calculated for C_58_H_109_N_13_O_21_^2+^: 661.89, found 662.0; [M + 3H]^3+^ calculated: 441.60, found 441.80; [M + 4H]^4+^ calculated: 331.45, found 331.60.

#### DFOB-*N*^2^-(PEG4-Ph-NCS)-l-Lys-*N*^6^-DOTA (3b) (D2-Ph-NCS)

To a solution of semi-pure 3 (D2) (114.9 mg, 86.93 μmol) in DMF (4.2 mL) was added triethylamine (242.0 μL, 1.74 mmol). This solution was added to a solution of 1,4-phenylenediisothiocyanate (165.9 mg, 0.86 mmol) in DMF (12.5 mL). After centrifugation (800 rpm, 90 min), the reaction mixture was aliquoted into 6 equal fractions and diethyl ether (9 mL) was added to each fraction. The fractions were cooled at 4 °C for 2 h and after centrifugation (4000 rpm, 5 min), the diethyl ether was decanted, and each fraction was washed with diethyl ether (10 mL). Excess diethyl ether was decanted, and the pellets were dried with N_2_. To the dry pellets was added DMF (196.3 μL), MeOH (1.47 mL) and diethyl ether (9 mL) before refrigerating for 16 h. Upon completion, the ether was removed, and the pellet was washed with diethyl ether (10 mL) and dried with N_2_. The pellets were dissolved in a mixture of ACN : H_2_O (3 : 7) and combined before further purification using HPLC. Fractions containing the product were lyophilised to yield D2-Ph-NCS (3b) as a white solid (32.5 mg, 21.47 μmol, 24.7%). ^1^H NMR (700 MHz, D_2_O) *δ* 7.36 (q, *J* = 8.7 Hz, 4H), 4.19 (dd, *J* = 8.7, 5.7 Hz, 1H), 3.76 (t, *J* = 6.2 Hz, 4H), 3.74–3.56 (m, 31H), 3.23–3.12 (m, 11H), 2.79 (td, *J* = 7.1, 2.7 Hz, 5H), 2.58–2.50 (m, 4H), 2.49 (t, *J* = 7.1 Hz, 5H), 2.13 (s, 3H), 1.72–1.61 (m, 5H), 1.61 (dd, *J* = 9.6, 5.7 Hz, 5H), 1.51 (ddt, *J* = 14.3, 10.6, 7.2 Hz, 12H), 1.34 (s, 3H), 1.29 (tp, *J* = 14.9, 7.9, 6.6 Hz, 8H). Acquired using water suppression. ^13^C NMR (700 MHz, D_2_O) *δ* 179.82, 174.84, 174.04, 173.93, 173.85, 173.63, 134.81, 126.91, 126.37, 69.77, 69.73, 69.66, 66.73, 55.33, 54.24, 51.59, 47.97, 47.93, 47.78, 44.32, 39.26, 39.20, 35.77, 30.84, 30.56, 28.05, 28.01, 27.98, 27.91, 27.77, 27.73, 25.57, 25.53, 23.14, 22.99, 22.63, 19.66, 19.34. HRMS (H-ESI II) *m*/*z*: [M + H]^+^ calculated for C_66_H_112_N_15_O_21_S_2_^+^: 1514.75931, found 1514.75500; [M + 2H]^2+^ calculated: 757.883295, found 757.881144; [M + 3H]^3+^ calculated: 505.591289, found 505.593039. A larger-scale sample of D2-Ph-NCS provided by AusPep as a custom synthesis (90% pure by RP-HPLC) was used for selected studies (Fig. 5–10; S12–S15, S26–S30 and Tables S1–S7†), with all remaining work using compounds synthesized in-house (Fig. 2–4, S1–S11 and S16–S25†).

## Data availability

All relevant data are presented in the main text and ESI† (general information, NMR spectra, LC-MS data, preparation of D2-mAb, 1-mAb, 2-mAb, radiolabelling, biology).

## Author contributions

The study was conceptualized by RC, MPW, and JLW. All authors contributed to the design of the study methods and the analysis of results. Experimental data was generated by JLW, ZHH, SG, NLF, JH, DTA, KM and WT. Clinical grade HuJ591 was provided by MPW and AI. The first draft of the manuscript was written by RC and JLW, with all authors contributing to manuscript review, editing, and data presentation, and approving the final submission.

## Conflicts of interest

The authors declare the following competing financial interest(s). Intellectual property related to this research includes Rachel Codd and James L. Wood as listed inventors. Michael P. Wheatcroft and Alesia Ivashkevich are employees of Telix Pharmaceuticals.

## Supplementary Material

SC-015-D4SC02851A-s001

## References

[cit1] Wadas T. J., Wong E. H., Weisman G. R., Anderson C. J. (2010). Chem. Rev..

[cit2] Price E. W., Orvig C. (2014). Chem. Soc. Rev..

[cit3] Kostelnik T. I., Orvig C. (2019). Chem. Rev..

[cit4] Boros E., Packard A. B. (2019). Chem. Rev..

[cit5] Bodei L., Herrmann K., Schöder H., Scott A. M., Lewis J. S. (2022). Nat. Rev. Clin. Oncol..

[cit6] Sgouros G., Bodei L., McDevitt M. R., Nedrow J. R. (2020). Nat. Rev. Drug Discovery.

[cit7] Morgan K. A., Rudd S. E., Noor A., Donnelly P. S. (2023). Chem. Rev..

[cit8] Hicks R. J., Jackson P., Kong G., Ware R. E., Hofman M. S., Pattison D. A., Akhurst T. A., Drummond E., Roselt P., Callahan J., Price R., Jeffery C. M., Hong E., Noonan W., Herschtal A., Hicks L. J., Hedt A., Harris M., Paterson B. M., Donnelly P. S. (2019). J. Nucl. Med..

[cit9] Cullinane C., Jeffery C. M., Roselt P. D., van Dam E. M., Jackson S., Kuan K., Jackson P., Binns D., van Zuylekom J., Harris M. J., Hicks R. J., Donnelly P. S. (2020). J. Nucl. Med..

[cit10] Captain I., Deblonde G. J.-P., Rupert P. B., An D. D., Illy M.-C., Rostan E., Ralston C. Y., Strong R. K., Abergel R. J. (2016). Inorg. Chem..

[cit11] Carter K. P., Deblonde G. J.-P., Lohrey T. D., Bailey T. A., An D. D., Shield K. M., Lukens W. W., Abergel R. J. (2020). Commun. Chem..

[cit12] Xu J., Cai F., Luo Z., Fan W., Dai J., Cui J., Li S., Geng C., Zheng Q., Wang Z., Tang X. (2022). Eur. J. Nucl. Med. Mol. Imaging.

[cit13] Wharton L., Zhang C., Yang H., Zeisler J., Radchenko V., Rodríguez-Rodríguez C., Osooly M., Patrick B. O., Lin K.-S., Bénard F., Schaffer P., Orvig C. (2022). Bioconjugate Chem..

[cit14] Vaughn B. A., Loveless C. S., Cingoranelli S. J., Schlyer D., Lapi S. E., Boros E. (2021). Mol. Pharmaceutics.

[cit15] Baranyai Z., Tircsó G., Rösch F. (2020). Eur. J. Inorg. Chem..

[cit16] Mease R. C., Kang C. M., Kumar V., Banerjee S. R., Minn I., Brummet M., Gabrielson K. L., Feng Y., Park A., Kiess A. P., Sgouros G., Vaidyanathan G., Zalutsky M. R., Pomper M. G. (2022). J. Nucl. Med..

[cit17] Dilworth J. R., Pascu S. I. (2018). Chem. Soc. Rev..

[cit18] Bhatt N. B., Pandya D. N., Wadas T. J. (2018). Molecules.

[cit19] Chomet M., van Dongen G. A. M. S., Vugts D. J. (2021). Bioconjugate Chem..

[cit20] Radchenko V., Busse S., Roesch F. (2014). Nucl. Med. Biol..

[cit21] Smith-Jones P. M., Stolz B., Bruns C., Albert R., Reist H. W., Fridrich R., Macke H. R. (1994). J. Nucl. Med..

[cit22] Mathias C. J., Lewis M. R., Reichert D. E., Laforest R., Sharp T. L., Lewis J. S., Yang Z.-F., Waters D. J., Snyder P. W., Low P. S., Welch M. J., Green M. A. (2003). Nucl. Med. Biol..

[cit23] Holland J. P. , in Handbook of Radiopharmaceuticals: Methodology and Applications, ed. M. R. Kilbourn and P. J. Scott, John Wiley & Sons Ltd, Hoboken, NJ, 2nd edn, 2021, ch. 11, pp. 343–374

[cit24] Pandey U., Gamre N., Kumar Y., Shetty P., Dev Sarma H., Dash A. (2016). J. Radioanal. Nucl. Chem..

[cit25] Marmion C. J., Griffith D., Nolan K. B. (2004). Eur. J. Inorg. Chem..

[cit26] Codd R. (2008). Coord. Chem. Rev..

[cit27] Bíró L., Buglyó P., Farkas E. (2021). Curr. Med. Chem..

[cit28] Patra M., Bauman A., Mari C., Fischer C. A., Blacque O., Haussinger D., Gasser G., Mindt T. L. (2014). Chem. Commun..

[cit29] Rudd S. E., Roselt P., Cullinane C., Hicks R. J., Donnelly P. S. (2016). Chem. Commun..

[cit30] Richardson-Sanchez T., Tieu W., Gotsbacher M. P., Telfer T. J., Codd R. (2017). Org. Biomol. Chem..

[cit31] Tieu W., Lifa T., Katsifis A., Codd R. (2017). Inorg. Chem..

[cit32] Brown C. J. M., Gotsbacher M. P., Codd R. (2020). Aust. J. Chem..

[cit33] Holland J. P. (2020). Inorg. Chem..

[cit34] Salih A. K., Raheem S. J., Dominguez Garcia M., Ahiahonu W. K., Price E. W. (2022). Inorg. Chem..

[cit35] Raavé R., Sandker G., Adumeau P., Jacobsen C. B., Mangin F., Meyer M., Moreau M., Bernhard C., Da Costa L., Dubois A., Goncalves V., Gustafsson M., Rijpkema M., Boerman O., Chambron J.-C., Heskamp S., Denat F. (2019). Eur. J. Nucl. Med. Mol. Imaging.

[cit36] Damerow H., Cheng X., von Kiedrowski V., Schirrmacher R., Wängler B., Fricker G., Wängler C. (2022). Pharmaceutics.

[cit37] Khozeimeh Sarbisheh E., Summers K. L., Salih A. K., Cotelesage J. J. H., Zimmerling A., Pickering I. J., George G. N., Price E. W. (2023). Inorg. Chem..

[cit38] Toporivska Y., Gumienna-Kontecka E. (2019). J. Inorg. Biochem..

[cit39] Borgias B., Hugi A. D., Raymond K. N. (1989). Inorg. Chem..

[cit40] Hernlem B. J., Vane L. M., Sayles G. D. (1996). Inorg. Chim. Acta.

[cit41] Cacheris W. P., Nickle S. K., Sherry A. D. (1987). Inorg. Chem..

[cit42] Kubíček V., Havlíčková J., Kotek J., Tircsó G., Hermann P., Tóth E., Lukeš I. (2010). Inorg. Chem..

[cit43] Pippin C. G., McMurry T. J., Brechbiel M. W., McDonald M., Lambrecht R., Milenic D., Roselli M., Colcher D., Gansow O. A. (1995). Inorg. Chim. Acta.

[cit44] Ma M. T., Meszaros L. K., Paterson B. M., Berry D. J., Cooper M. S., Ma Y. M., Hider R. C., Blower P. J. (2015). Dalton Trans..

[cit45] Pandya D. N., Bhatt N., Yuan H., Day C. S., Ehrmann B. M., Wright M., Bierbach U., Wadas T. J. (2017). Chem. Sci..

[cit46] Pandya D. N., Henry K. E., Day C. S., Graves S. A., Nagle V. L., Dilling T. R., Sinha A., Ehrmann B. M., Bhatt N. B., Menda Y., Lewis J. S., Wadas T. J. (2020). Inorg. Chem..

[cit47] Aime S., Botta M., Ermondi G. (1992). Inorg. Chem..

[cit48] Aime S., Botta M., Fasano M., Marques M. P. M., Geraldes C. F. G. C., Pubanz D., Merbach A. E. (1997). Inorg. Chem..

[cit49] Blahut J., Hermann P., Tošner Z., Platas-Iglesias C. (2017). Phys. Chem. Chem. Phys..

[cit50] Briand M., Aulsebrook M. L., Mindt T. L., Gasser G. (2017). Dalton Trans..

[cit51] Guillou A., Earley D. F., Klingler S., Nisli E., Nüesch L. J., Fay R., Holland J. P. (2021). Bioconjugate Chem..

[cit52] Novak D., Tomašič T., Krošelj M., Javornik U., Plavec J., Anderluh M., Peitl P. K. (2021). ChemMedChem.

[cit53] Giese M. W. , Woodman R. H., Hermanson G. T. and Davis P. D., in Chemical Linkers in Antibody–Drug Conjugates (ADCs), ed. F. L. van Delft and J. M. Lambert, The Royal Society of Chemistry, 2022, vol. 81, ch. 9, pp. 286–376

[cit54] King H. D., Yurgaitis D., Willner D., Firestone R. A., Yang M. B., Lasch S. J., Hellström K. E., Trail P. A. (1999). Bioconjugate Chem..

[cit55] Liao G., Zhou Z., Burgula S., Liao J., Yuan C., Wu Q., Guo Z. (2015). Bioconjugate Chem..

[cit56] Abate-Shen C., Nunes de Almeida F. (2022). Cancer Res..

[cit57] Nanus D. M., Milowsky M. I., Kostakoglu L., Smith-Jones P. M., Vallabahajosula S., Goldsmith S. J., Bander N. H. (2003). J. Urol..

[cit58] Viola-Villegas N. T., Sevak K. K., Carlin S. D., Doran M. G., Evans W. H., Bartlett D. W., Wu A. M., Lewis J. S. (2014). Mol. Pharmaceutics.

[cit59] Vallabhajosula S., Smith-Jones P. M., Navarro V., Goldsmith S. J., Bander N. H. (2004). Prostate.

[cit60] Merkx R. I. J., Lobeek D., Konijnenberg M., Jiménez-Franco L. D., Kluge A., Oosterwijk E., Mulders P. F. A., Rijpkema M. (2019). Eur. J. Nucl. Med. Mol. Imaging.

[cit61] Merkx R. I. J., Rijpkema M., Franssen G. M., Kip A., Smeets B., Morgenstern A., Bruchertseifer F., Yan E., Wheatcroft M. P., Oosterwijk E., Mulders P. F. A., Heskamp S. (2022). Pharmaceuticals.

[cit62] Vallabhajosula S., Nikolopoulou A., Jhanwar Y. S., Kaur G., Tagawa S. T., Nanus D. M., Bander N. H., Goldsmith S. J. (2016). Curr. Radiopharm..

[cit63] White J. M., Escorcia F. E., Viola N. T. (2021). Theranostics.

[cit64] DeNardo G. L., Kennel S. J., Siegel J. A., Denardo S. J. (2004). Clin. Lymphoma.

[cit65] Chomet M., Schreurs M., Bolijn M. J., Verlaan M., Beaino W., Brown K., Poot A. J., Windhorst A. D., Gill H., Marik J., Williams S., Cowell J., Gasser G., Mindt T. L., van Dongen G. A. M. S., Vugts D. J. (2021). Eur. J. Nucl. Med. Mol. Imaging.

[cit66] Eberlein U., Cremonesi M., Lassmann M. (2017). J. Nucl. Med..

