# Supplementary material for: A first-in-class dual-chelator theranostic agent designed for use with imaging-therapy radiometal pairs of different elements
Source: Chem Sci. 2024 Jun 3;15(30):11748–60. doi: 10.1039/d4sc02851a (PMC11290327; doi:10.1039/d4sc02851a)
Supplement: SC-015-D4SC02851A-s001 [file SC-015-D4SC02851A-s001.pdf]

Electronic Supplementary Material (ESI) for Chemical Science.
This journal is © The Royal Society of Chemistry 2024

Electronic Supplementary Information

A first-in-class dual-chelator theranostic agent designed for use with imaging-therapy radiometal pairs of different elements

James L. Wood,[a,b] Saikat Ghosh,[b] Zachary H. Houston,[b] Nicholas L. Fletcher,[b] James Humphries,[b] Karine Mardon,[b] Dewan T. Akhter,[b] William Tieu,[c] Alesia Ivashkevich,[d] Michael P. Wheatcroft,[d] Kristofer J. Thurecht,[b] and Rachel Codd*[a]

[a] The University of Sydney, School of Medical Sciences, New South Wales 2006, Australia

[b] Centre for Advanced Imaging (CAI), Australian Institute for Bioengineering and Nanotechnology (AIBN) and ARC Training Centre for Innovation in Biomedical Imaging Technology, The University of Queensland, Brisbane, Queensland 4072, Australia

[c] Molecular Imaging and Therapy Research Unit (MITRU), South Australian Health and Medical Research Institute (SAHMRI), Adelaide, Australia

[d] Telix Pharmaceuticals Limited, North Melbourne, Victoria 3051, Australia

Corresponding Author: Email: rachel.codd@sydney.edu.au

ITEM		pg
**Table of contents**	....	**S1**
**General information**	....	**S3**
**Instrumentation**	....	**S3**
**Solid-phase extraction**	....	**S3**
**HPLC purification**	....	**S3**
**NMR spectroscopy**	....	**S4**
**Preparation of solutions for {^{1}H}-^{13}C NMR spectroscopy**	....	**S4**
**LC-MS spectrometry**	....	**S4**
**Preparation of solutions for LC-MS spectrometry**	....	**S5**
**Antibody conjugation and radiolabelling studies I**	....	**S5**
**Antibody conjugation and radiolabelling studies II**	....	**S6**
**Cell binding assays**	....	**S7**
**Serum stability**	....	**S8**
**Animal studies**	....	**S8**
**Animals**	....	**S8**
**Tumour initiation and growth**	....	**S8**
**PET/CT Imaging**	....	**S8**
**SPECT/CT Imaging**	....	**S9**
**Image Data Processing and Analysis**	....	**S9**
**Statistical Analysis**	....	**S9**
**Figure S1.** {^{1}H}-^{13}C NMR (600 MHz, D_2O) spectrum of DFOB (**1**).	....	**S10**
**Figure S2.** {^{1}H}-^{13}C NMR (600 MHz, D_2O) spectrum from a Zr(IV):**1** (1.8:1) solution.	....	**S10**
**Figure S3.** {^{1}H}-^{13}C NMR (600 MHz, D_2O) spectrum from a Lu(III):**1** (1.3:1) solution.	....	**S11**
**Figure S4.** ^{1}H-NMR (600 MHz, D_2O) spectrum from a **3a** solution.	....	**S11**
**Figure S5.** COSY-NMR (600 MHz, D_2O) spectrum from a **3a** solution.	....	**S12**
**Figure S6.** HSQC-NMR (600 MHz, D_2O) spectrum from a **3a** solution.	....	**S12**
**Figure S7.** HMBC-NMR (600 MHz, D_2O) spectrum from a **3a** solution.	....	**S13**
**Figure S8.** {^{1}H}-^{13}C NMR (600 MHz, D_2O) spectrum from a **3a** solution.	....	**S13**
**Figure S9.** {^{1}H}-^{13}C NMR (600 MHz, D_2O) spectrum from a Zr(IV):**3a** (0.8:1) solution.	....	**S14**
**Figure S10.** {^{1}H}-^{13}C NMR (600 MHz, D_2O) spectrum from a Lu(III):**3a** (0.8:1) solution.	....	**S14**
**Figure S11.** {^{1}H}-^{13}C NMR (600 MHz, DMSO-d_6) spectrum from a solution of **3** (**D2**).	....	**S15**
**Figure S12.** ^{1}H-NMR (700 MHz, D_2O) spectrum from a **3b** solution.	....	**S15**

**Figure S13.** {^{1}H}-^{13}C NMR (700 MHz, D_2O) spectrum from a **3b** solution.	....	**S16**
**Figure S14.** HSQC NMR (700 MHz, D_2O) spectrum from a **3b** solution.	....	**S16**
**Figure S15.** HMBC NMR (700 MHz, D_2O) spectrum from a **3b** solution.	....	**S17**
**Figure S16.** {^{1}H}-^{13}C NMR (600 MHz, D_2O) spectrum from a **8a** solution.	....	**S17**
**Figure S17.** Stacked {^{1}H}-^{13}C NMR (600MHz, D_2O) spectra of solutions of (**a**) **8a**, or (**b**) Zr(IV):**8a**, or (**c**) Lu(III):**8a**.	....	**S18**
**Figure S18.** Stacked {^{1}H}-^{13}C NMR (600 MHz, D_2O) spectra from solutions of (**a**) **8a**, or (**b**) **3a**.	....	**S18**
**Figure S19.** LC-MS traces of **3a** (M = $C_{47}H_{86}N_{12}O_{16}$) shown as (**a**) total ion current (TIC), or as extracted ion chromatograms (EIC) set to report (**b**) $[M+H]^+$ (1075.1-1076.1), (**c**) $[M+2H]^{2+}$ (538.3-539.3), or (**d**) $[M+3H]^{3+}$ (358.7-359.7).	....	**S18**
**Figure S20.** (**a**) High resolution average mass spectrum (R_t = 7.37-7.98 min (refer panel (**a**) of **Figure S19**)) of **3a** (M = $C_{47}H_{86}N_{12}O_{16}$), and experimental (black) and calculated (gray) isotope patterns for marked signals (**b**–**g**) consistent with adducts: (**b**) $[M+3H]^{3+}$, (**c**) $[M+2H]^{2+}$, (**d**) $[M-H+Fe]^{2+}$ (0.35) and $[M+2H+3H_2O]^{2+}$ (0.65), (**e**) $[M+H]^+$, (**f**) $[M+Na]^+$, or (**g**) $[M-2H+Fe]^+$ (0.35) and $[M+H+3H_2O]^+$ (0.65). The signals marked with an asterisk are consistent with MS2 fragments of **3a** formed during spectral acquisition (as per scheme below).	....	**S19**
**Figure S21.** LC-MS traces of **3** (M = $C_{58}H_{107}N_{13}O_{21}$) shown as (**a**) total ion current (TIC), or as extracted ion chromatograms (EIC) set to report (**b**) $[M+2H]^{2+}$ (661.6-662.6), (**c**) $[M+3H]^{3+}$ (441.3-442.4), or (**d**) $[M+4H]^{4+}$ (331.1-332.1).	....	**S19**
**Figure S22.** (**a**) Low resolution average mass spectrum (R_t = 6.68-9.24 min (refer panel (**a**) of **Figure S21**)) of **3** (M = $C_{58}H_{107}N_{13}O_{21}$), and experimental (black) and calculated (gray) isotope patterns for marked signals (**b**–**d**) consistent with adducts: (**b**) $[M+4H]^{4+}$, (**c**) $[M+3H]^{3+}$, (**d**) $[M+2H]^{2+}$.	....	**S20**
**Figure S23.** LC-MS traces of **3b** (M = $C_{66}H_{111}N_{15}O_{21}S_2$) shown as (a) total ion current (TIC), or as extracted ion chromatograms (EIC) set to report (b) $[M+H]^+$ (1514.3-1515.3), (c) $[M+2H]^{2+}$ (757.3-758.3), or (d) $[M+3H]^{3+}$ (505-506).	....	**S20**
**Figure S24.** (a) High resolution average mass spectrum (R_t = 9.86-11.93 min (refer panel (a) of **Figure S23**)) of **3** (M = $C_{66}H_{111}N_{15}O_{21}S_2$), and experimental (black) and calculated (gray) isotope patterns for marked signals (**b**–**g**) consistent with adducts: (b) $[M+3H]^{3+}$, (c) $[M+2H]^{2+}$, (d) $[M-H+Fe]^{2+}$, (e) $[M+H]^+$, (f) $[M+Na]^+$, or (g) $[M-2H+Fe]^+$. The signals marked with an asterisk are consistent with MS2 fragments of **3** formed during spectral acquisition (as per scheme below).	....	**S21**
**Figure S25.** Radio-iTLC (a–e) or radio-SEC-HPLC (f) traces from radiolabelling (a) **D2**-mAb with ^{89}Zr, (b) **D2**-mAb with ^{177}Lu, (c) **1**-mAb with ^{89}Zr, (d) **2**-mAb with ^{177}Lu, (e) **2**-mAb with ^{89}Zr; or (f) **2**-mAb (red) or **1**-mAb (black) with ^{177}Lu. In these experiments, mAb = girentuximab.	....	**S21**
**Figure S26.** Representative MALDI TOF-MS of **1**-mAb (a), **2**-mAb (b) or **D2**-mAb (c) after reaction at 37 °C for 3 h.	....	**S22**
**Figure S27.** Stability by radio-iTLC of [^{89}Zr]Zr-**D2** (a–d) or [^{89}Zr]Zr-**1** (e–h) in human serum at day 0, 2, 4, 7.	....	**S22**
**Figure S28.** Stability by radio-SEC-HPLC of [^{89}Zr]Zr-**D2**-mAb (a–d) or [^{89}Zr]Zr-**1**-mAb (e–h) in human serum at day 0, 2, 4, 7.	....	**S22**
**Figure S29.** Stability by radio-iTLC of [^{89}Zr]Zr-**D2** (a–d) or [^{89}Zr]Zr-**1** (e–h) in phosphate buffered saline at day 0, 2, 4, 7.	....	**S23**
**Figure S30.** Stability by radio-SEC-HPLC of [^{89}Zr]Zr-**D2**-mAb (a–d) or [^{89}Zr]Zr-**1**-mAb (e–h) in phosphate buffered saline at day 0, 2, 4, 7.	....	**S23**
**Table S1.** C.A.R values for **D2**-mAb, **1**-mAb and 2-mAb conjugates.	....	**S23**
**Table S2.** Raw values for % cell associated activity of all radiolabelled conjugates (n = 3).	....	**S23**
**Table S3.** Stability of [^{89}Zr]Zr-**D2**-mAb or [^{89}Zr]Zr-**1**-mAb in human serum or PBS at day 0, 2, 4, 7.	....	**S24**
**Table S4.** Mouse identifiers and injected dose for *ex-vivo* biodistribution studies. Approximate mass dose calculated using activity measured from initial labelling reaction.	....	**S24**
**Table S5.** Mouse identifiers and injected dose for imaging studies. Approximate mass dose calculated using activity measured from initial labelling reaction.	....	**S24**
**Table S6.** *In vivo* biodistribution (*n* = 3) of [^{89}Zr]Zr-**1**-mAb and [^{89}Zr]Zr-**D2**-mAb at 4 h, 24 h, 48 h, or 120 h post-injection; or [^{89}Zr]Zr-**D2**-mAb and [^{177}Lu]Lu-**D2**-mAb at 48 h, or 120 h post-injection.	....	**S25**
**Table S7.** *Ex vivo* biodistribution (*n* = 3) of [^{89}Zr]Zr-**1**-mAb and [^{89}Zr]Zr-**D2**-mAb at (**a**) 48 h p.i. or (**b**) 120 h p.i., or (**c**) [^{89}Zr]Zr-**D2**-mAb and [^{177}Lu]Lu-**D2**-mAb (**5**) at 120 h p.i.	....	**S25**
**Table S8.** Mouse identifiers and dosing for efficacy study using [^{177}Lu]Lu-**D2**-mAb (n=4), [^{177}Lu]Lu-**2**-mAb (n=3) and a vehicle control (n=3). A target of 9 MBq was used for ^{177}Lu groups. Approximate mass dose calculated using activity measured from initial labelling reaction.	....	**S26**
**Table S9.** Statistical data from efficacy study using [^{177}Lu]Lu-**D2**-mAb, [^{177}Lu]Lu-**2**-mAb, and PBS vehicle control	....	**S26**
**Table S10.** Radiolabelling parameters used in study components.	....	**S28**

General information

All reactions were carried out under ambient conditions and were monitored by ESI or H-ESI II MS spectrometry (Agilent 1260, Agilent 1290 or Thermo Fisher Vanquish systems).

Instrumentation

Solid-phase extraction

Solid-phase extraction procedures were performed on a manual vacuum manifold, or semi-automatically on a Grace Reverleris X2 autoflash chromatography platform, or a Biotage Selekt autoflash chromatography platform. Manual extractions used Waters Sep-Pak Vac 20cc C18 cartridges (variable weight) and the following method: 100% acetonitrile (ACN) for 2 column volumes (CV), 100% H_2O for 2 CV, sample load, 100% H_2O for 2 CV, 20-80% ACN:H_2O in 5% steps of 2 CV each step, 95% ACN:H_2O for 2 CV. Grace Reverleris X2 procedures used Buchi FlashPure Select C18 cartridges (30 µm particle size, 4 g) at a flow rate of 10 mL min^{-1} and the following method: 100% ACN for 4 min, 100% H_2O for 2.4 min, sample load, 100% H_2O for 2 min, 20-80% ACN:H_2O in 5% steps at 3 min each step, 95% ACN:H_2O for 3 min. Biotage Selekt procedures used Sfär C18 D-Duo cartridges (30 µm particle size, 6 g, 100 Å) and the following method: equilibration with 100% ACN, 100% ACN for 2 CV, equilibration with H_2O, sample load, 100% H_2O for 2 CV, 20-80% ACN:H_2O in 5% steps of 2 CV each step, 95% ACN:H_2O for 2 CV. Fractions were identified *via* UV absorbance (Grace Reverleris, Biotage Selekt) and the products were characterised using LC-MS (all methods).

HPLC purification

Preparative high-performance liquid chromatography (HPLC) was conducted on a Shimadzu LC-20 series LC system with two LC-20AP pumps, an SIL-10AP autosampler, an SPD-20A UV/Vis detector, and a FRC-10A fraction collector. The organic phase (B) consisted of ACN:formic acid (FA) or trifluoroacetic acid (TFA) (percentage varied per method, as below). The aqueous phase (A) consisted of H_2O:FA or TFA (percentage varied per method, as below). Data was acquired and processed using Shimadzu LabSolutions Software (version 5.98). Purification methods were optimised for each compound, as below.

Compound **3a**: Shimadzu Shimpack GIS column (150 × 20 mm i.d., 20 mL min^{-1}, particle size 5 µm) with gradient of 2-15% solvent B over 10 min, 15-23% solvent B over 15 min, 2% solvent B for 5 min. TFA (0.05% was used in each phase).

Compound **3** (**D2**): Shimadzu Shimpack GIS column (150 × 20 mm i.d., 20 mL min^{-1}, particle size 5 µm) with gradient of 5-35% solvent B over 25 min, 95% solvent B over 5 min, 5% solvent B for 5 min. FA (0.05% was used in each phase).

Compound **3b** (**D2**-Ph-NCS): Shimadzu Shimpack GIS column (150 × 10 mm i.d., 5 mL min^{-1}, particle size 5 µm) with gradient of 20-25% solvent B over 5 min, 25-45% solvent B over 35 min, 20% solvent B for 5 min. FA (0.05% was used in each phase).

Compound **8a**. Shimadzu Shimpack GIS column (150 × 20 mm i.d., 20 mL min^{-1}, particle size 5 µm) with a gradient of 2-15% solvent B over 10 min, 15-23% solvent B over 15 min, 2% solvent B for 5 min. FA (0.1% was used in each phase).

NMR spectroscopy

NMR spectra were obtained on two instrument platforms. All spectra obtained in a 600 MHz field strength were acquired at 298 K using a Bruker AVIII 600MHz, narrow-bore, 3-channel NMR spectrometer equipped with a high-resolution cryogenic triple nucleus probehead (H/C/N) and a high-throughput autosampler with individual rack temperature control. All samples were prepared in D_2O or DMSO-d_6. Spectral data were acquired using Bruker TopSpin (v.3.6.5) and processed using Bruker TopSpin (v.4.1.3) and Mestrelab MestReNova (v. 14.3.0-30573 or v. 14.1.0-24037). Denoising, automatic baselining and automatic phasing were applied to all spectra.
All spectra obtained in a 700 MHz field strength were acquired at 298K using a Bruker Avance 700 MHz, narrow-bore, 3-channel NMR spectrometer equipped with a high-resolution cryogenic triple nucleus probehead (H/C/N). All samples were prepared in D_2O. Spectral data were acquired using Bruker TopSpin (v. 3.6.5) and processed using MestReNova (v. 14.1.0-24037). Water suppression was applied to the ^1H spectrum. Automatic baselining and automatic phasing were applied to all spectra.

Preparation of solutions for {^1H}-^{13}C NMR spectroscopy

Zr(IV)-DFOB. A solution of DFOB in D_2O (36.24 mM) was added in equal portion to a solution of $ZrCl_4$ (66.08 mM) in D_2O. The solution was mixed and sonicated for 2.5 h prior to NMR analysis.

Lu(III)-DFOB. A solution of DFOB in D_2O (43.09 mM) was added in equal portion to a solution of $LuCl_3$ (55.45 mM) in D_2O. The solution was mixed and sonicated for 2.5 h prior to NMR analysis.

Zr(IV)-3a or Lu(III)-3a. A solution of **3a** in D_2O (14.18 mM) was added in equal portion to a solution of D_2O, $ZrCl_4$ (14.19 mM) or $LuCl_3$ (14.18 mM). The resulting mixtures were briefly centrifuged, and the solutions were stirred at 400 rpm for 1 h at 37 °C prior to NMR analysis.

LC-MS spectrometry

Mass spectra were obtained using one of three instruments (Instrument A, B or C) with an Agilent C18 column reverse-phased prepacked column (2.1 × 150 mm i.d., 0.3 mL min^{-1}, particle size 3.5 µm) used for all experiments. **Instrument A:** reverse-phase liquid chromatography-mass spectrometry instrument consisting of an autoinjector (100 µL loop), an Agilent 1260 Infinity degasser, a quaternary pump, a temperature-controlled column compartment and an Agilent 6120 series quadrupole electrospray ionization (ESI)-mass spectrometer. Conditions and methods: 5 µL injection volume, column oven = 28 °C, full MS scan 100-2000 *m*/*z*, capillary voltage = 3 kV, capillary temperature = 350 °C. The organic phase (B) consisted of ACN:FA 99.9:0.1, and the aqueous phase (A) consisted of H_2O:FA 99.9:0.1. The method used a gradient of 5–95% solvent B over 20 min,

95% solvent B for 5.1 min and 95% solvent A for 4.9 min with a flow rate of 0.3 mL min^{-1}. Spectral data were acquired and processed using Agilent OpenLAB Chromatography Data System ChemStation Edition (C.01.05 SP1 [61]). **Instrument B:** reverse-phase liquid chromatography-mass spectrometry instrument consisting of a 1290 series quaternary pump with inbuilt degasser, a 1200 series autosampler, a temperature-controlled column compartment, a diode array detector, and a 6460 series triple quadrupole electrospray ionisation (ESI)-mass spectrometer. Conditions and methods: The settings, composition of the organic and aqueous phase, and flow rate were as above. The method used a gradient of 5–95% solvent B over 20 min, 95% solvent B for 5 min and 95% solvent A for 5 min. Spectral data were acquired and processed using Agilent MassHunter Workstation Qualitative Analysis (10.0.10305.10). **Instrument C:** Thermo Fisher Vanquish Horizon UHPLC with a Thermo Fisher Q Exactive HF-X Hybrid Quadruple-Orbitrap Spectrometer. Conditions and methods: Column oven = 30 °C; full MS scan 133.4-2000 *m*/*z* with a resolution of 60000, MS-MS collision energies stepped from 20, 25 to 30 V, spray voltage = 4 kV, capillary temperature = 300 °C. The organic phase (B) consisted of ACN:H_2O:FA 80:19.9:0.1, and the aqueous phase (A) consisted of H_2O:FA 99.9:0.1. The method used a gradient of 5–95% solvent B over 20 min, 95% solvent B for 5.1 min and 100% solvent A for 4.9 min with a flow rate of 0.2 mL min^{-1}. Spectral data were acquired and processed using Thermo Fisher Xcalibur (4.2.47).

Preparation of solutions for LC-MS spectrometry

Samples for the **3a** metal-binding experiments used solutions of Lu(III) or Ga(III) and **3a** prepared in 0.2 M $NH_4CH_3CO_2$ buffer (pH 5.5), and solutions of Zr(IV) and **3a** prepared in 0.1 M HEPES buffer (pH 7.4). An aliquot of a solution of **3a** (0.93 mM in HEPES buffer (0.1 M, pH 7.4) or in $NH_4CH_3CO_2$ buffer (0.2 M, pH 5.5)) was added to an equal aliquot of a solution of $NH_4CH_3CO_2$ buffer (0.2 M, pH 5.5), HEPES buffer (0.1 M, pH 7.4), Zr(IV) (0.74 mM in HEPES buffer (0.1 M, pH 7.4)), Lu(III) (0.74 mM in $NH_4CH_3CO_2$ buffer (0.2 M, pH 5.5)) or Ga(III) (0.74 mM in $NH_4CH_3CO_2$ buffer (0.2 M, pH 5.5)). All samples were incubated at 400 rpm, 37 °C for 1 h prior to subsampling for LCMC analysis.

Antibody conjugation and radiolabelling studies I

D2-mAb, and DFOB-mAb. A modified literature procedure (M. J. W. D. Vosjan, L. R. Perk, G. W. M. Visser, M. Budde, P. Jurek, G. E. Kiefer and G. A. M. S. van Dongen, *Nat. Protoc.*, 2010, **5**, 739–743) was used as follows. In this preliminary study, the mAb used was girentuximab. 6% v/v 1 M Na_2CO_3 was added to girentuximab (mAb) (4.8 mg mL^{-1}) in the supplied buffer. Compound **3b** (**D2**-Ph-NCS) or DFOB-Ph-NCS (**1**-Ph-NCS) in DMSO (20 µL, 5 mM) was added and the reaction mixture was incubated at 37 °C at 550 rpm for 45 min. The reaction mixture was purified *via* Amicon spin filtration (10 kDa cutoff) and washed with Dulbecco's phosphate buffered saline (DPBS) buffer (pH 7.4). **DOTA-mAb (2-mAb).** A modified literature procedure (T. Basaco, S. Pektor, J. M. Bermudez, N. Meneses, M. Heller, J. A. Galván, K. F. Boligán, S. Schürch, S. von Gunten, A. Türler and M. Miederer, *Pharmaceuticals (Basel)*, 2018, **11**, 132) was used as follows. Girentuximab (mAb) (4.98 mg mL^{-1}, 500 µL) was buffer exchanged with 4.0 v/v% of 0.1 M Na_2CO_3 in DPBS buffer (pH 8.5). DOTA-Bn-NCS (**2**-Bn-NCS) (1.25 mg) and 70 µL of 0.1 M Na_2CO_3 were added to

the mAb to give a reaction solution of pH 8.5. The reaction solution was shaken on a thermomixer at 500 rpm for 50 min and purified *via* Amicon spin filtration (10 kDa MWCO) and washed with DPBS buffer (pH 7.4). **Radiolabelling with ^{89}Zr.** Zr-89 (20 µL/9.5 MBq) was neutralised with 1 M Na_2CO_3 (18 µL) and buffered with 0.5 M HEPES (200 µL). **D2**-mAb, **1**-mAb or **2**-mAb was added and the solution was placed on a thermomixer at 550 rpm at ambient temperature. Radiochemical labelling efficiency was determined by iTLC. Detailed radiolabelling parameters can be found in Table S10.

Radiolabelling with ^{177}Lu. A fixed quantity of [^{177}Lu]$LuCl_3$ (5 µL, 15 MBq) was added to an aliquot of 0.5 M NH_4OAc (100 µL) and the solution was gently shaken. A corresponding quantity of **D2**-mAb, **1**-mAb or **2**-mAb was added and the solution was placed on a thermomixer at 550 rpm at ambient temperature. The aliquot was incubated with 1 mM EDTA (pH 6.0) for 5 min and the reaction was monitored by iTLC with 25 mM EDTA (pH 6.0) or SEC-HPLC. **Radio-SEC-HPLC and iTLC analysis.** Radio-SEC-HPLC analysis was performed using a LabLogic Flow-RAM attachment with 1" NaI detector. For iTLC analysis, all samples were treated prior with EDTA (1 mM for 5 min). An aliquot (1 µL) of sample was spotted on an iTLC-SG plate and developed with 0.1 M citrate buffer (pH 5.5) eluent. iTLC plates were analysed using LabLogic Scan-RAM with a Plastic Scintillator PMT detector. Detailed radiolabelling parameters can be found in Table S10.

Antibody conjugation and radiolabelling studies II

3b (D2-Ph-NCS), or **1**-Ph-NCS, or **2**-Bn-NCS (~15-fold molar excess) dissolved in Milli-Q water (**3b, 2**) or DMSO (**1**) (~1 mg mL^{-1}) was added to HuJ591 in 0.1 M $NaHCO_3$, pH 9.25 (~5 mg mL^{-1}). The reaction mixture was allowed to react for 1 h at 37 °C while being gently agitated at 500 rpm. The resulting mixture was buffered exchanged into Milli-Q water using Amicon Spin Membranes (30 kDa MWCO, 0.5 mL). The resultant solution was buffer exchanged into 0.1 M NH_4OAc, pH 5.5 for ^{177}Lu coordination, or 0.1 M HEPES, pH 7.4 for ^{89}Zr coordination using a desalting column (Cytiva PD-10 G-25 Sephadex resin). The purity of the resulting **D2**-mAb, **1**-mAb or **2**-mAb complexes was analysed using SEC-HPLC (UV detection at 280 nm) and the chelator-to-antibody ratio (C.A.R) was determined for each antibody by matrix-assisted laser desorption ionisation-time of flight mass spectrometry (MALDI-TOF/TOF, JOEL Spiral TOF). The average C.A.R was determined by comparing the difference in *m/z* values of the $[M+2H]^{2+}$ peak of the free mAb and ligand-mAb conjugate. The difference was divided by the molar mass of the ligand to give the average C.A.R. **D2**-mAb was found to produce conjugates with higher C.A.R values than **1**-mAb or **2**-mAb, however the distribution of C.A.R appeared broader. **Radiolabelling with ^{177}Lu.** $^{177}LuCl_3$ (ANSTO) was dispensed *via* the addition of HCl (0.04 M, 200 µL) to the provided vial. The resultant solution was buffered with the addition of an equal volume of NH_4OAc (0.5 M, pH 5.5). For reaction, the desired conjugate was introduced to ^{177}Lu in a molar excess (see Table S10) and agitated at 500 rpm, 37°C for 1 h. Upon completion, the radiochemical purity was assessed by radio-iTLC and injected into mice if the radiochemical purity was ≥95%. The reaction solution was analysed via radio-SEC-HPLC to further assess radiochemical purity and possible antibody aggregation. For injection, 10× PBS was added (~10% total reaction volume) and then 1× PBS was added to make the final injection solution suitable for ~200 µL injections of the desired activity required. If conjugate clean-up was required, the

conjugate was buffer exchanged into 1× PBS using a desalting column (Cytiva PD-10 G-25 Sephadex resin) and used as was. **Radiolabelling with ^{89}Zr.** ^{89}Zr-oxalate (0.05 M oxalic acid, Austin Health) was adjusted to pH 12-13 with Na_2CO_3 (0.1 M, pH 11.5). The solution was further buffered with the addition of HEPES (0.5 M, pH 7) to bring the pH to 7-8. For reaction, the desired conjugate was introduced to ^{89}Zr in a molar excess (see Table S10) and agitated at 500 rpm, 37°C for 1 h. Upon completion, the radiochemical purity was assessed by radio-iTLC and injected into mice if the radiochemical purity was ≥95%. 5 µl of reaction solution was mixed with 5 µl of 5 mM DTPA and analysed *via* radio-SEC-HPLC to further assess radiochemical purity and possible antibody aggregation. For injection, 10× PBS was added (~10% total reaction volume) and then 1× PBS was added to make the final injection solution suitable for ~200 µL injections of the desired activity required. **Radio-iTLC.** Samples radiolabelled with ^{89}Zr or ^{177}Lu were taken and mixed 1:1 with EDTA or DTPA (5 mM). Each TLC solution (with and without EDTA or DTPA) was spotted on TLC paper (Agilent iTLC-SG Glass microfiber chromatography paper impregnated with silica gel) and run in 1:1 ethanol:water. Plates were then imaged on an Eckert & Ziegler Mini-Scan and Flow-Count iTLC Reader.

Cell binding assays

Seeding and incubation. LNCaP cells were seeded at a density of 7.5×10^4 cells/well in a 24-well plate with a final number of approximately 2.5×10^5 cells per well. Radiolabelled mAbs ([^{89}Zr]Zr-**D2**-mAb (**4**), [^{177}Lu]Lu-**D2**-mAb (**5**), [^{89}Zr]Zr-**1**-mAb, or [^{177}Lu]Lu-**2**-mAb) were diluted in serum-free cell growth medium (0.02 µg, 0.56 MBq) and 100 µL of each solution was added to each well. The cells were incubated in triplicate for 1 h at 37 °C in a 5% CO_2 atmosphere in a humidified incubator. **Determining the membrane-bound fraction.** At each time point, internalisation was halted by removing the growth medium and washing the cells twice with ice-cold phosphate-buffered saline (PBS) (1×, pH 7.4, 200 µL). Receptor-bound radiolabelled mAb was then removed using ice-cold glycine buffer containing 4 M urea (0.2 M, pH 2.0, 200 µL) for 5 min. The buffer was collected from each well and the radioactivity was measured in a gamma counter to determine the membrane-bound fraction. The cells were then washed once with the same glycine buffer. **Determining the internalised fraction.** Cells were treated with sodium hydroxide (1 N, 200 µL) for 30 min to lyse the cells, internalised fractions collected, and the radioactivity measured of the subsequent fractions in a gamma-counter. **Non-specific binding.** Cells were counted after the assay from 4 non-experimental wells and the cell numbers were averaged to obtain an estimate of the number of cells per well. Non-specific binding and internalisation were determined by co-incubating cells with non-radiolabelled (2 µg, 50 µL) and radio-labelled (0.02 µg, 50 µL) compound for each well, and repeating the above procedures for membrane-bound and internalised fractions.

Serum stability

Samples of [^{89}Zr]Zr-**D2**-mAb (**4**), [^{177}Lu]Lu-**D2**-mAb (**5**), [^{89}Zr]Zr-**1**-mAb, or [^{177}Lu]Lu-**2**-mAb were mixed with an equal volume of phosphate buffered saline (1× PBS), or human serum and incubated at 37 °C for 7 d. At day 0, 2, 4 and 7, the samples were analysed *via* radio-iTLC and radio-SEC-HPLC for mAb degradation. Radio-iTLC traces were challenged with equal volume of 5 mM EDTA to capture any free radioisotope.

Animal studies

Animals. Healthy male Balb/c nude mice (~20 g) from 8 weeks old were obtained from Ozgene. Mice were imported into the Centre for Advanced Imaging animal holding facility and monitored for 1 week to acclimatise to the environment prior to the injection of cells. All animals were provided with free access to food and water before and during the imaging experiments which were approved by the University of Queensland Animal Ethics Committee (AEC Approval # 2022/AE000135).

Tumour initiation and growth. 8-Week-old male Balb/c nude mice were injected (27G needle) subcutaneously with LNCaP (10×10^6) cells in 50 µL of 50:50 matrigel and PBS into the right flank of each mouse. There was no evidence of ulceration at the time of dosing. The animals were closely monitored and remained in good condition apart from the growth of tumours. The tumour growth was observed to be in-line with expected timelines and good tumours were ultimately observed in 100% of inoculated animals. [^{89}Zr]Zr-**D2**-mAb (**4**) or [^{89}Zr]Zr-**1**-mAb were injected *via* the tail vein (29G needle; ~1-4 MBq) and then mice were imaged using the Bruker Si78 PET/CT instrument at the various timepoints for ^{89}Zr and were sacrificed at 120 h p.i and their harvested organs counted *via* gamma counting. [^{177}Lu]Lu-**D2**-mAb (5) was injected *via* the tail vein (29G needle; ~1-4 MBq) and then mice were imaged using the Molecubes SPECT/CT instrument at the various timepoints for ^{177}Lu and were sacrificed at 120 h and their harvested organs counted *via* gamma counting. For 2-day *ex-vivo* biodistribution studies, [^{89}Zr]Zr-**D2**-mAb (**4**) or [^{89}Zr]Zr-**1**-mAb were injected *via* the tail vein (29G needle; ~1-4 MBq) and then the mice were sacrificed at 48 h p.i and their harvested organs counted *via* gamma counting.

Efficacy study. LNCaP tumour bearing male Balb/c nude mice (n=4 per ^{177}Lu-labelled cohort, n=5 per vehicle only cohort) were injected with [^{177}Lu]Lu-**D2**-mAb, [^{177}Lu]Lu-**2**-mAb or PBS. Mice were given a target of 9 MBq ^{177}Lu (29 G, tail vein injection in ~150 uL PBS) as per dosing information in Table S8. Activity in the syringe was measured using a dose calibrator (Capintec CRC-25). Mice were euthanised due to tumour size at day 40 (n=1), day 42 (n=1), day 52 (n=1) day 75 (n=1) and day 77 (n=1) in accordance with animal welfare guidelines described within the ethics approval (AEC Approval # 2022/AE000135). Mice with initial tumour weights deemed to be outliers were removed from data processing (n=1 for [^{177}Lu]Lu-**2**-mAb cohort, n=2 for vehicle only cohort). Percentage change in tumour volume and mouse weight were analysed over a period of day 1 to day 65 post-injection to reflect the time period prior to euthanasia of the vehicle only cohort.

PET/CT Imaging. A total of 6 (n=3 per group) LNCaP tumour bearing Balb/C nude mice were anaesthetised using an anaesthetic chamber (3% isoflurane in oxygen at a flow of 2L/min, IsoFlo, Abbott Laboratories), and injected intravenously with [^{89}Zr]Zr-D2-NCS-HuJ591/[^{89}Zr]Zr-DFO-NCS-HuJ591 (3.42-3.36 GBq/µmol) *via* the lateral tail vein. At defined timepoints post-administration of the compound, animals were placed in the Si78 PET/CT scanner (Bruker, Germany) for PET-CT evaluation of the tracer biodistribution. Mice were maintained under 1 to 2% isoflurane in air-oxygen mixture at a flow rate of 2 L/min for the duration of the imaging session and monitored *via* a respirator monitor (SA Instrument, USA). At each timepoint post-injection of the radiotracer, CT acquisitions were acquired and used to construct attenuation corrected images. Mice were scanned at 4-, 24-, 48- and 120-hours post-injection. The CT images were acquired through an X-ray source

with the voltage set to 60 kV and the current set to 600 µA with an isotropic resolution of 200 µm. The total CT scanning process took approximately 5 minutes. The CT images were reconstructed using a Feldkamp conebeam back-projection algorithm using Paravision 360 (version 3.4) (Bruker, Germany). For the PET data acquisition, the emission data were normalised and corrected for decay. The resulting sinograms were reconstructed with Paravision 360 (v3.4) using a 0.5mm 3D-maximum likelihood expectation-maximisation (MLEM) iterative image reconstruction algorithm. PET/CT image analysis was performed using PMOD version 4.4 (PMOD Technologies, Zurich, Switzerland).

SPECT/CT Imaging. *In vivo* micro SPECT/CT imaging was performed using an X-CUBE (CT) and ɣ-CUBE (SPECT) from Molecubes (Gent, Belgium). Mice were anaesthetised using 2% isoflurane in a constant flow of oxygen at 1 L/min, after which the isoflurane concentration was reduced to 1% during imaging. Mice were injected intravenously with [^{177}Lu]Lu-D2-NCS-HuJ591 (1.85 GBq/µmol) *via* the lateral tail vein. Vital signs (body temperature and respiration) of the mice were continuously monitored during both SPECT and CT scanning procedures. After completing a CT scan (200 µm isotropic spatial resolution), the animal bed was transferred to the γ-CUBE for static SPECT imaging (30-45 min), with energy peak for ^{177}Lu set at 208 keV and a window of ±10%. *In vivo* SPECT/CT imaging was carried out at 48- and 120-hours post-injection. SPECT images were acquired using a high sensitivity collimator, data was reconstructed through a maximum-likelihood expectation-maximisation (MLEM) algorithm using 500 µm resolution and 50 iterations using Molecubes software. A q-factor was determined from a volume of interest (VOI) that was drawn around the calibration tube containing a known activity of ^{177}Lu and represents the ratio of counts/cc to activity/cc. This q-factor was saved and applied during image reconstruction prior to generating SUV decay-corrected images. Attenuation maps were created from CT images at 500 µm isotropic spatial resolution and applied to SPECT images during reconstruction. SPECT/CT image analysis was performed using PMOD version 4.4 (PMOD Technologies, Zurich, Switzerland).

Image data processing and analysis. Fusion of CT and PET images and definition of region of interest (ROIs) were performed using PMOD version 4.4 (Bruker, Germany). For each PET images, 3D region of interest (VOI) were drawn over organs of interest guided by the CT. Activity per voxel was converted to nCi/cc using a conversion factor obtained by scanning a cylindrical phantom filled with a known activity of ^{89}Zr to account for PET scanner efficiency. Activity concentrations were then expressed as percent of the decay-corrected injected activity per cm^3 of tissue that can be approximated as percentage injected dose per gram of tissue (%ID/g). All data were decay corrected to the time of injection of the radiotracer. The mean value in each VOI was used to generate regional time activity curves (TACs). Individual TACs were normalised by the injected dose and results were expressed as %ID/g**.**

Statistics analysis. Statistical analysis was conducted within GraphPad Prism v.10.1.4 (324). Each analysis was conducted as an unpaired t test with Welch correction and assuming a Gaussian distribution. An Alpha value of 0.05 was set as the threshold for the P value with correction for multiple comparisons done using the Holm-Šídák method. *p ≤ 0.05, **p ≤ 0.01, ***p ≤ 0.001.

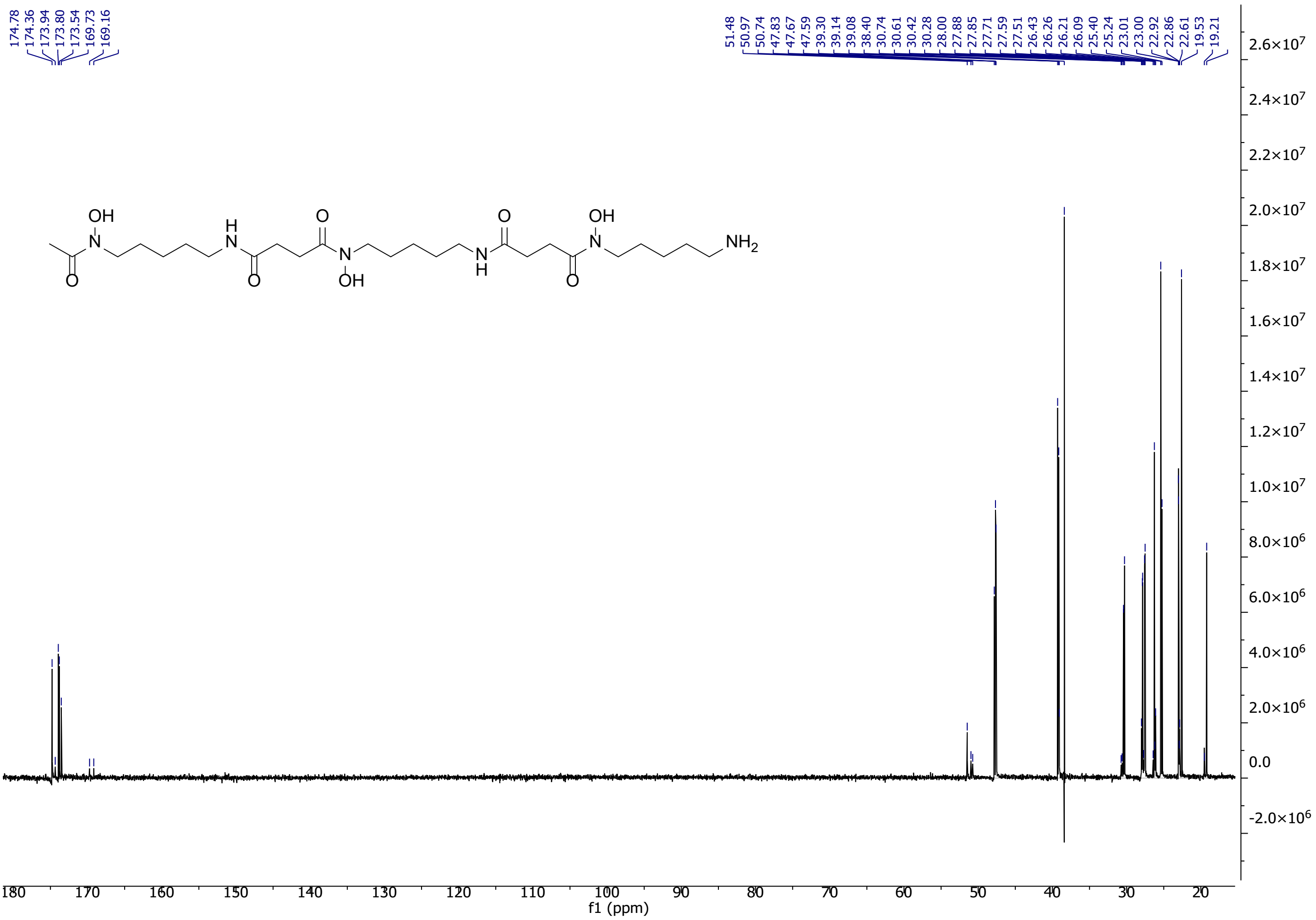

Figure S1. {^{1}H}-^{13}C NMR (600 MHz, D_2O) spectrum of DFOB (**1**). The signal at 38.4 ppm is due to a decoupler zero frequency spike.

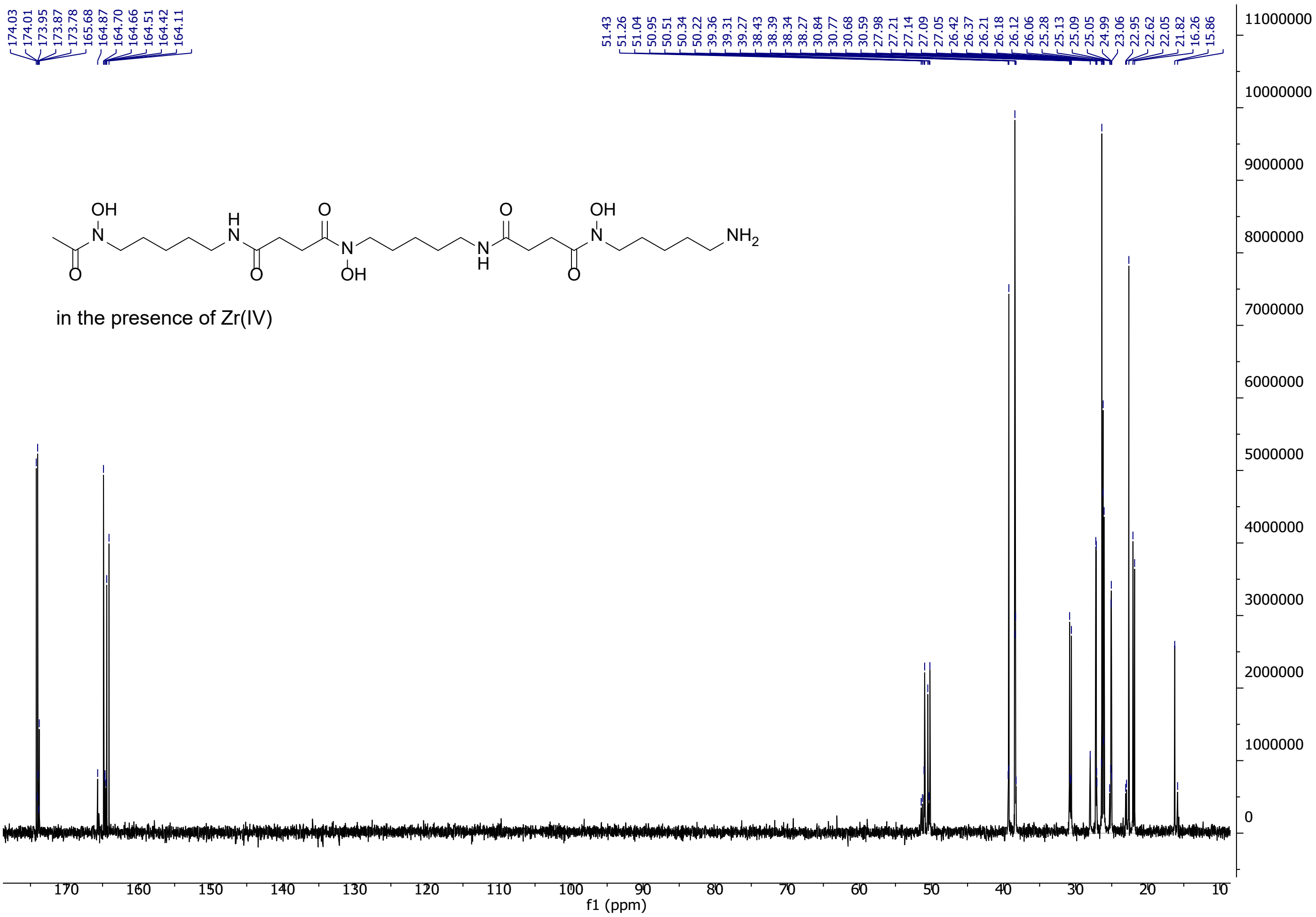

Figure S2. {^{1}H}-^{13}C NMR (600 MHz, D_2O) spectrum from a Zr(IV):**1** (1.8:1) solution.

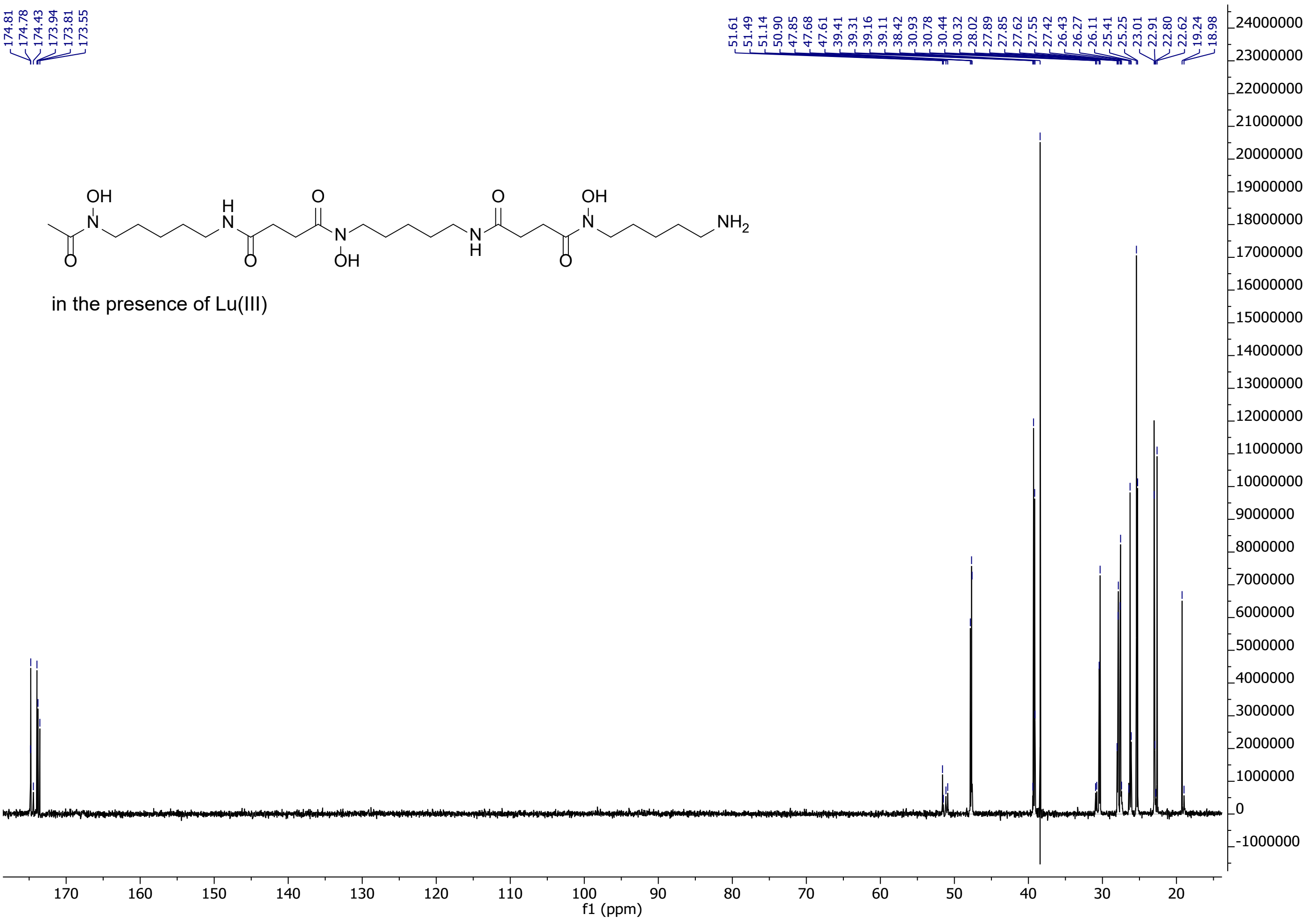

Figure S3. {^{1}H}-^{13}C NMR (600 MHz, D_2O) spectrum from a Lu(III):**1** (1.3:1) solution. The signal at 38.4 ppm is due to a decoupler zero frequency spike.

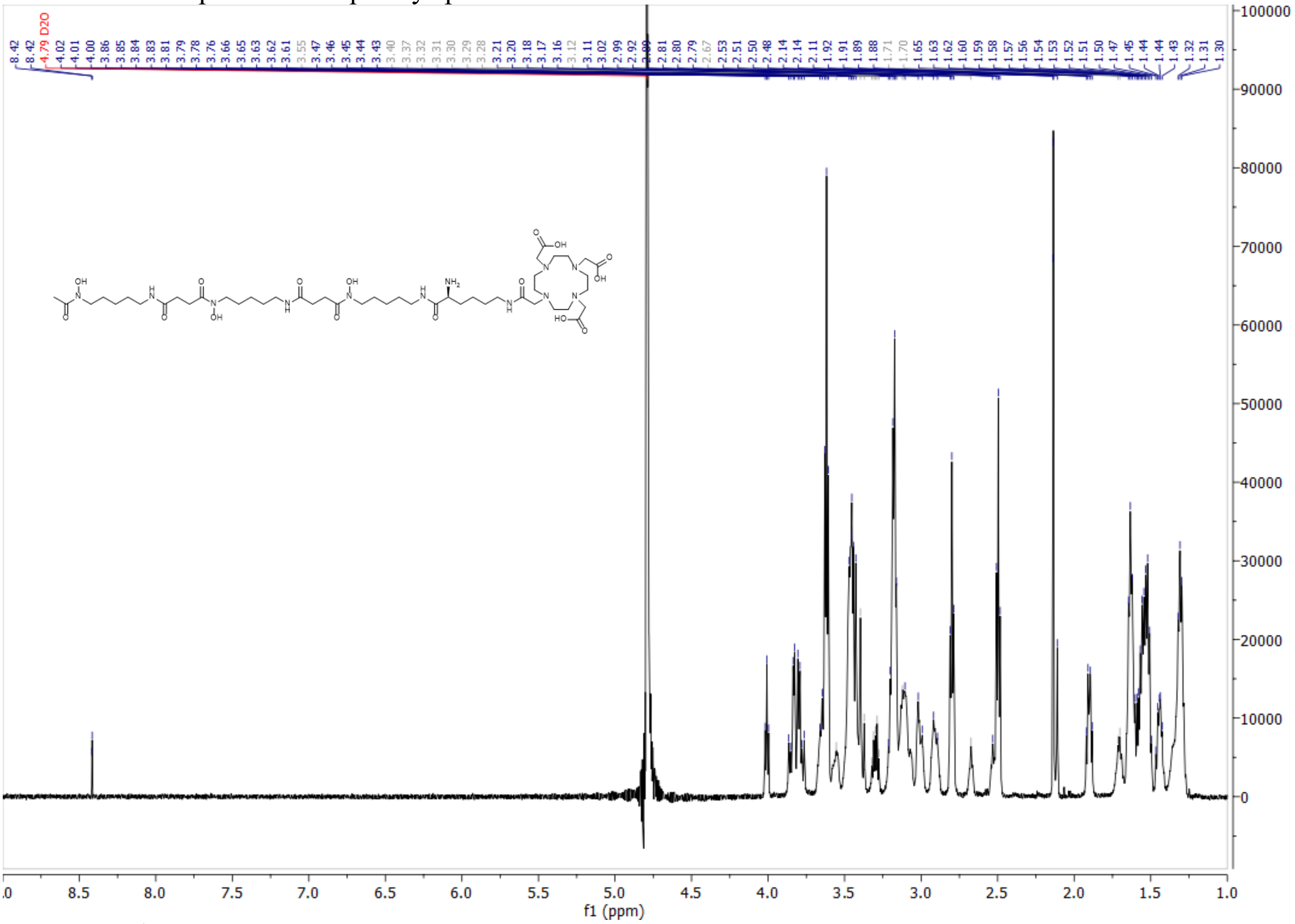

Figure S4. ^{1}H-NMR (600 MHz, D_2O) spectrum from a **3a** solution.

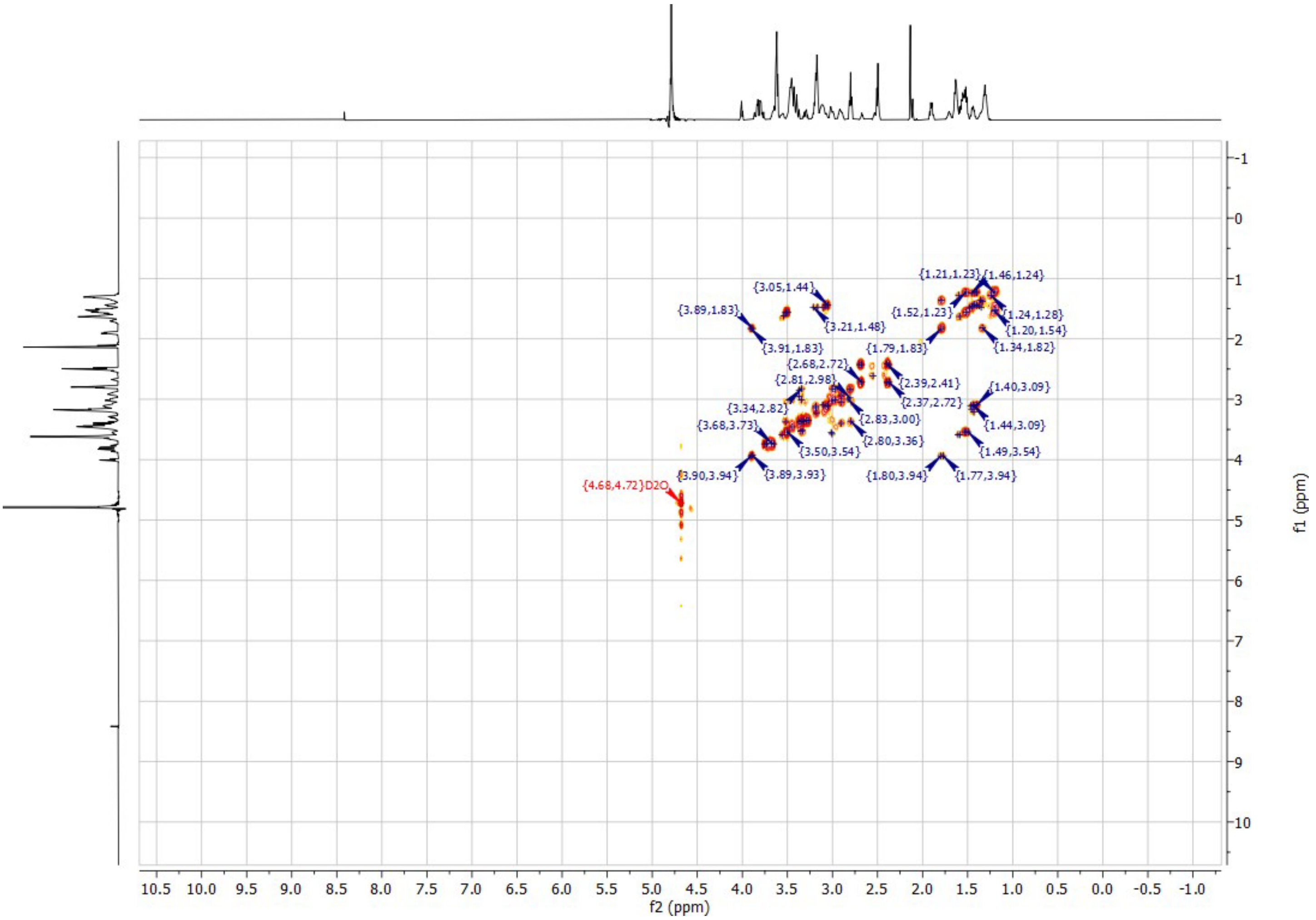

Figure S5. COSY-NMR (600 MHz, D_2O) spectrum from a **3a** solution.

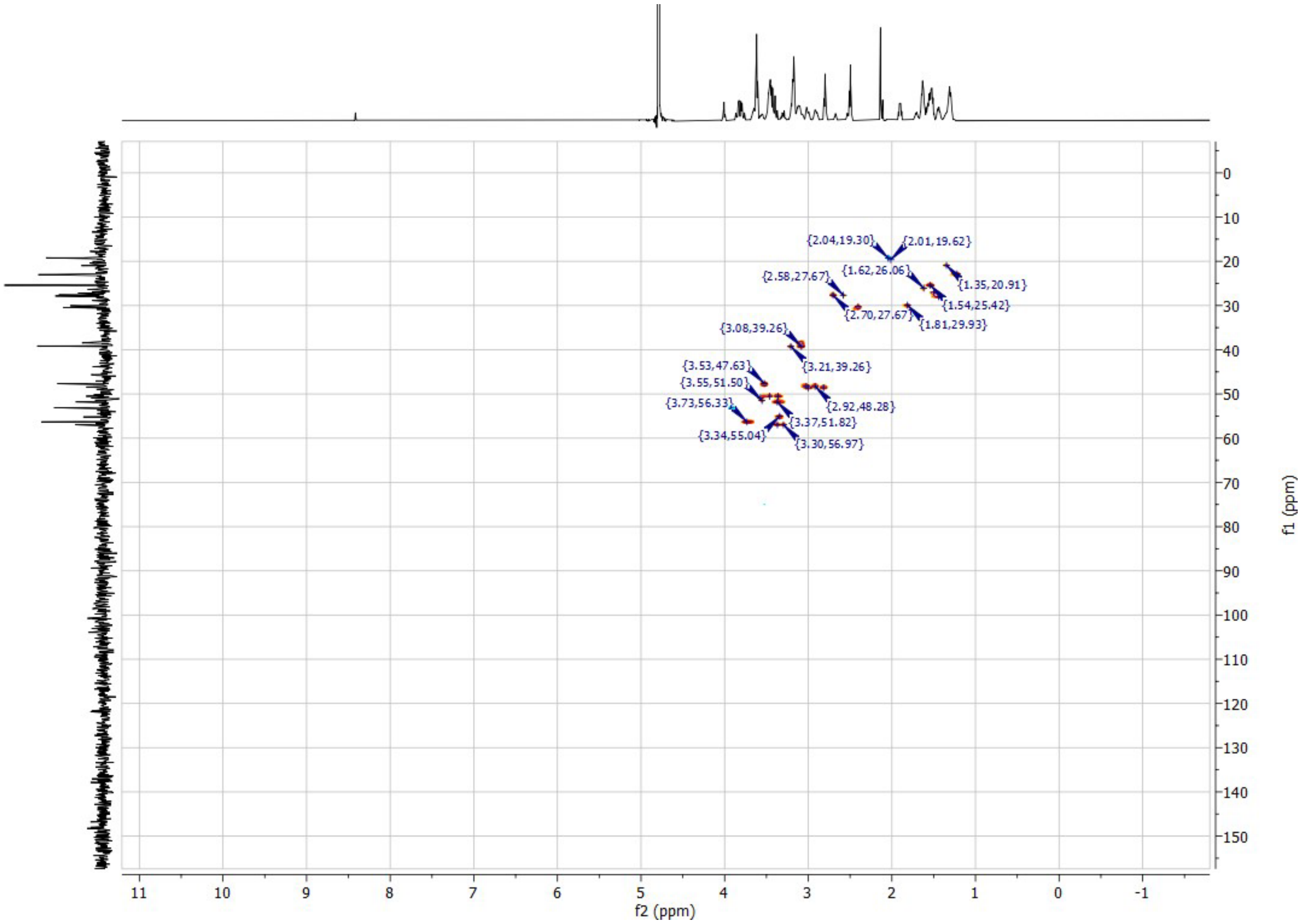

Figure S6. HSQC-NMR (600 MHz, D_2O) spectrum from a **3a** solution.

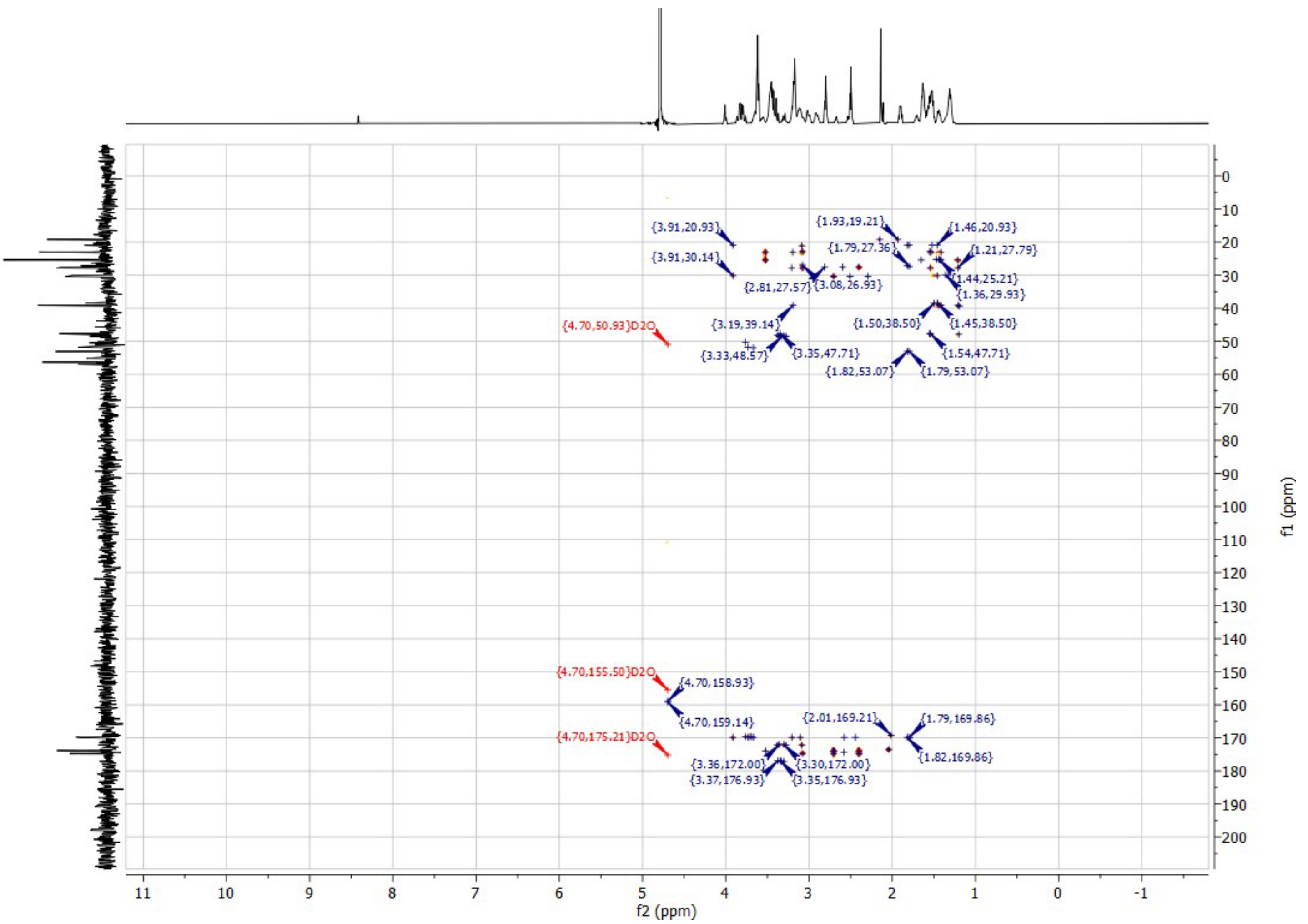

Figure S7. HMBC-NMR (600 MHz, D_2O) spectrum from a **3a** solution.

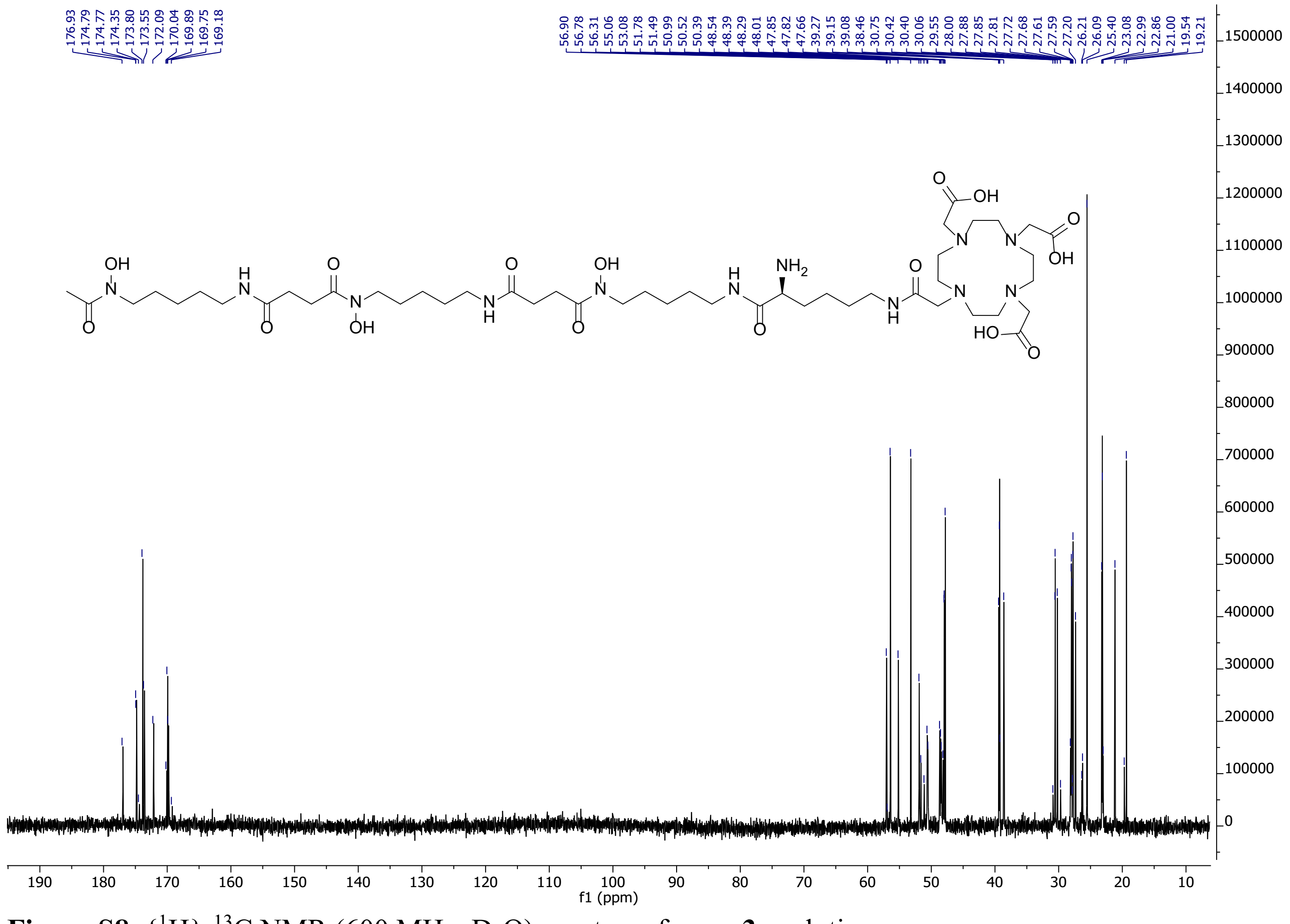

Figure S8. {^{1}H}-^{13}C NMR (600 MHz, D_2O) spectrum from a **3a** solution.

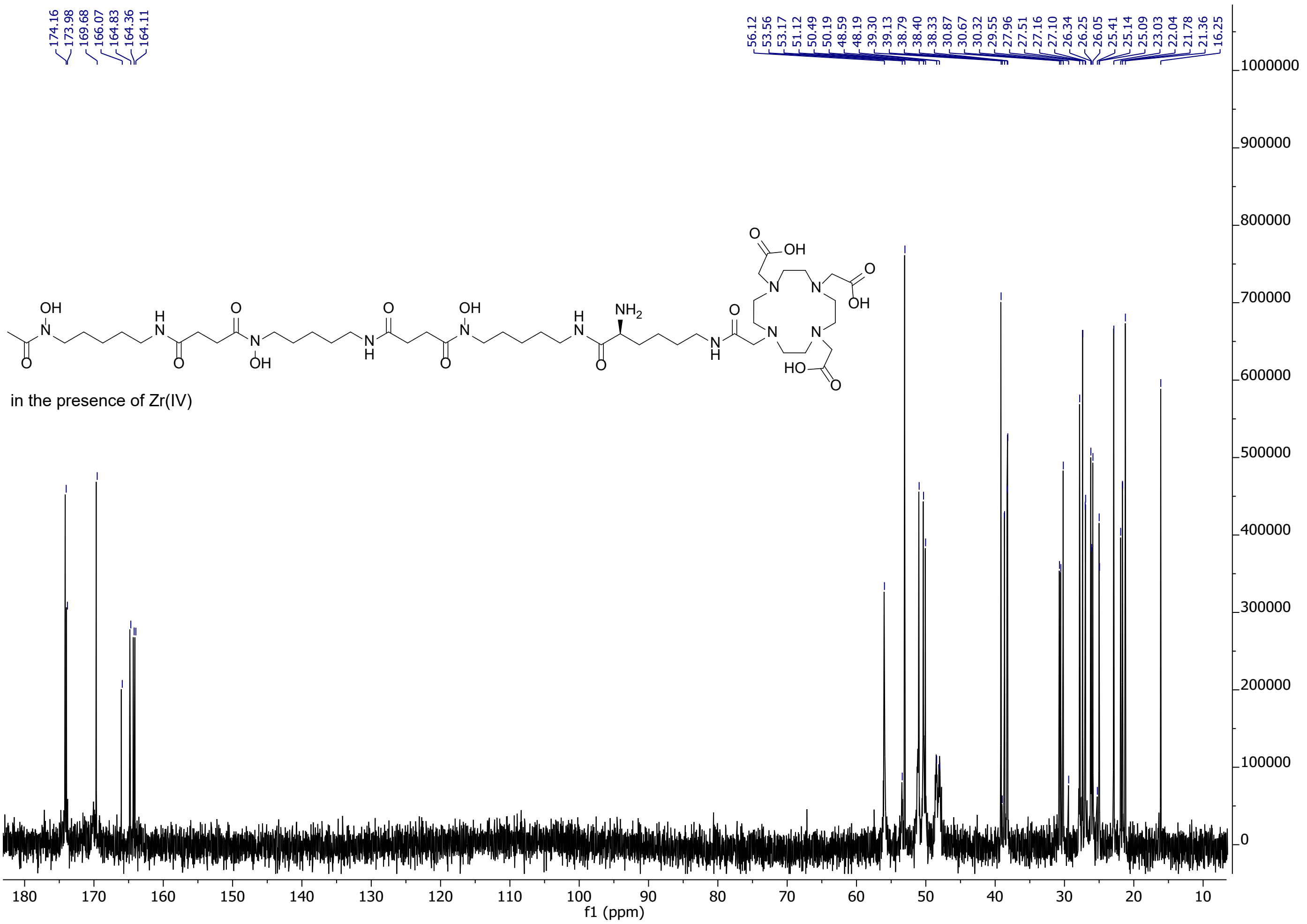

Figure S9. {^{1}H}-^{13}C NMR (600 MHz, D_2O) spectrum from a Zr(IV):**3a** (0.8:1) solution.

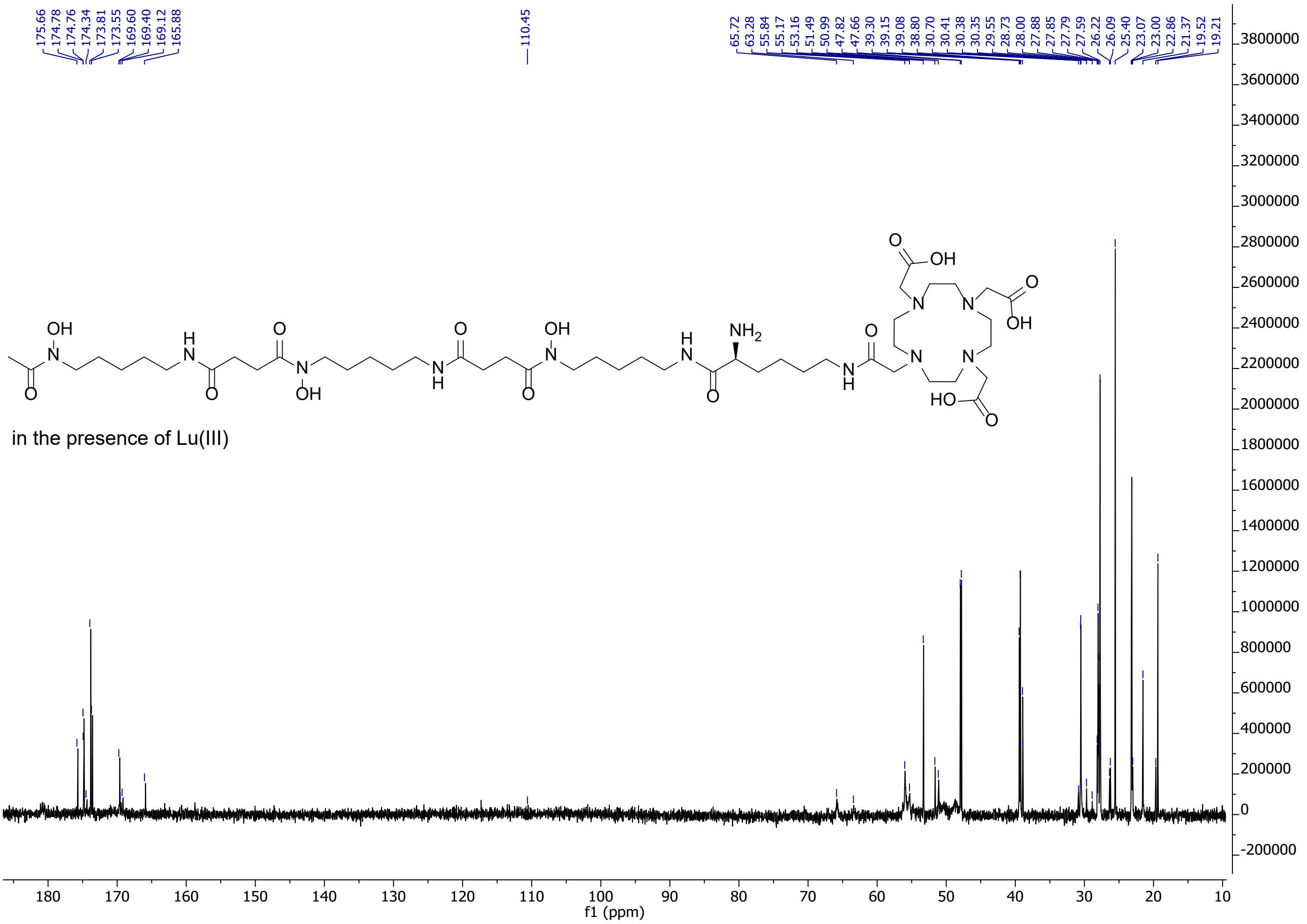

Figure S10. {^{1}H}-^{13}C NMR (600 MHz, D_2O) spectrum from a Lu(III):**3a** (0.8:1) solution.

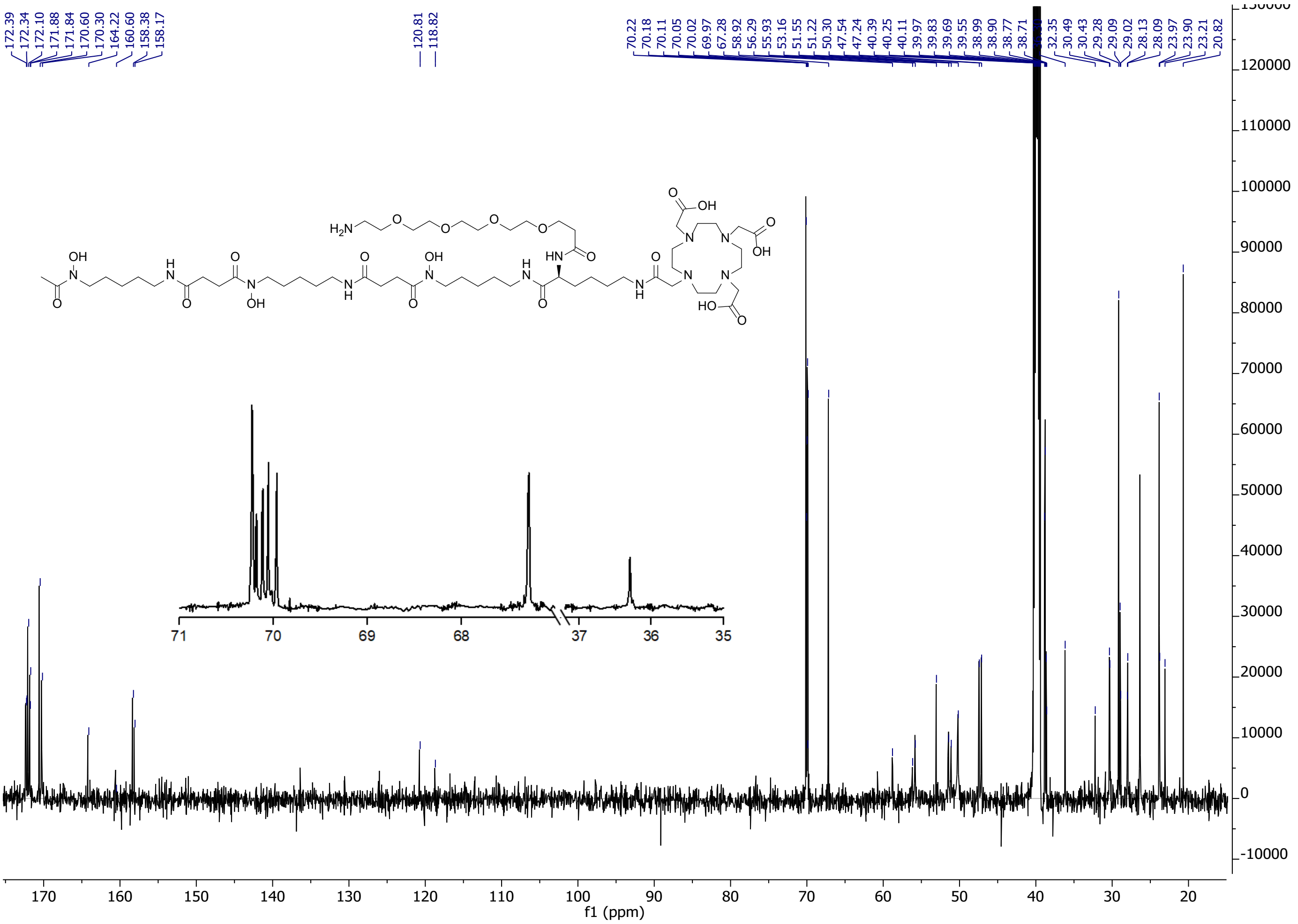

Figure S11. {^{1}H}-^{13}C NMR (600 MHz, DMSO-d_6) spectrum from a solution of **3** (**D2**), with (inset) signals ascribed to the PEG4 region.

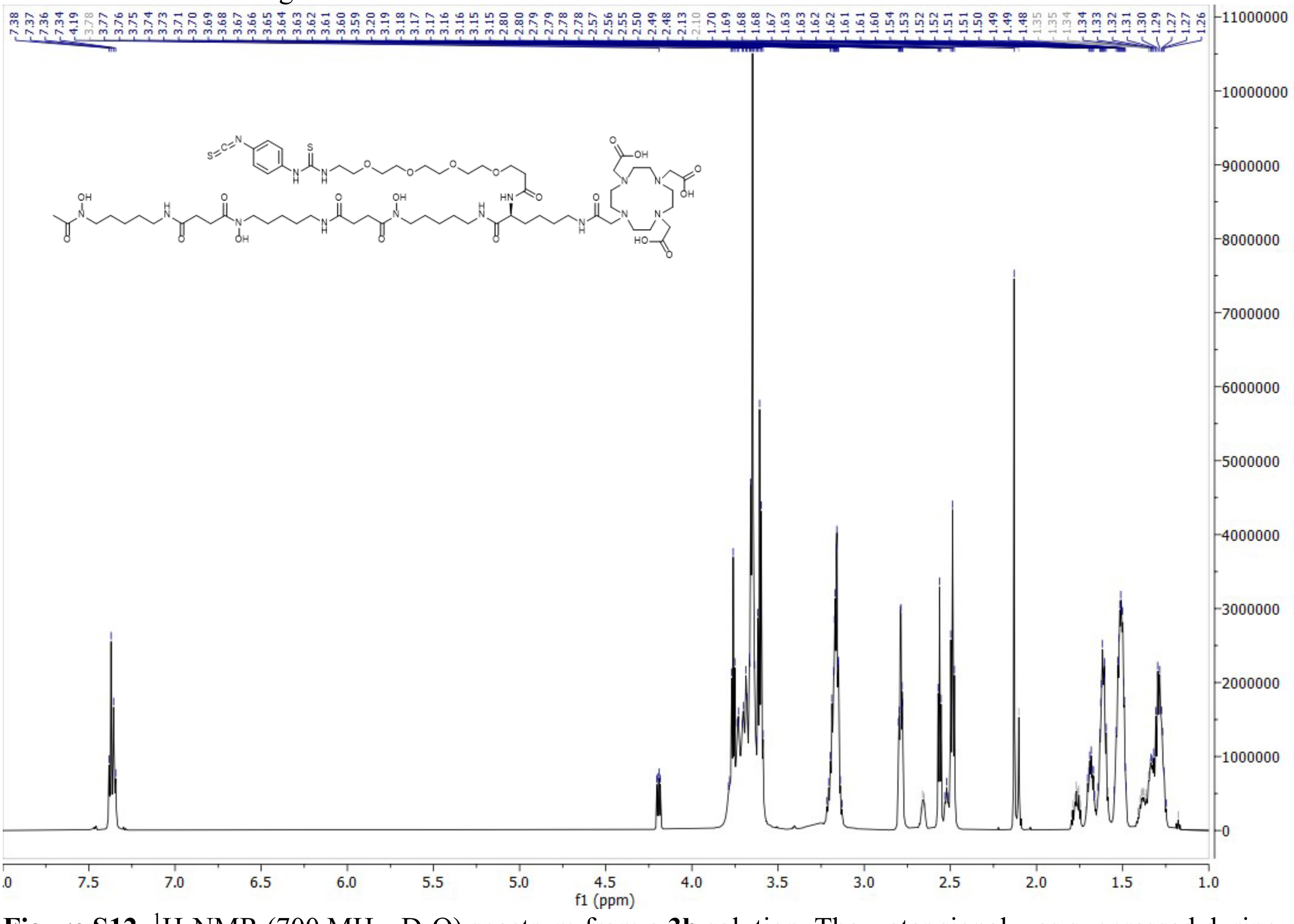

Figure S12. ^{1}H-NMR (700 MHz, D_2O) spectrum from a **3b** solution. The water signal was suppressed during acquisition.

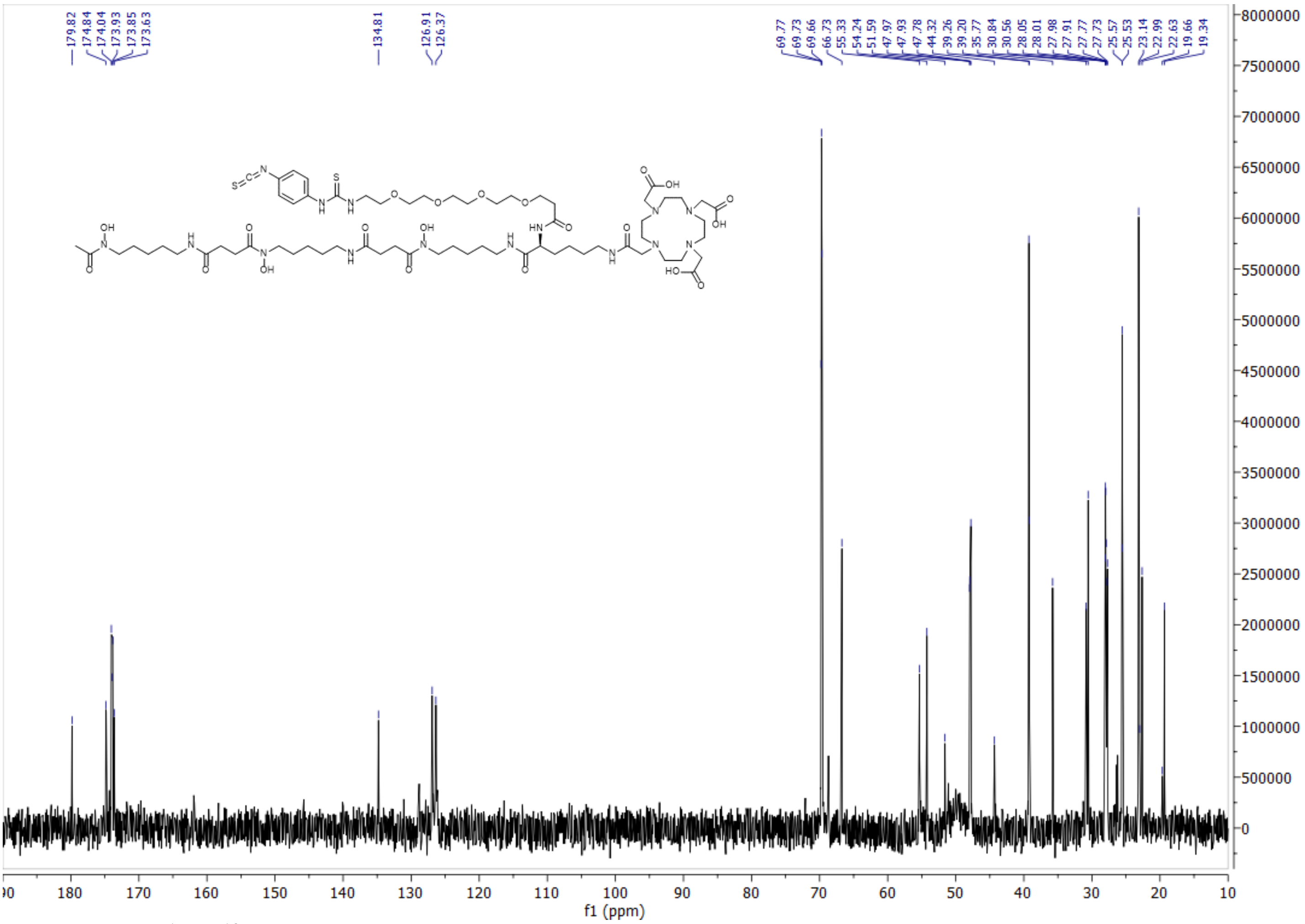

Figure S13. {^{1}H}-^{13}C NMR (700 MHz, D_2O) spectrum from a **3b** solution.

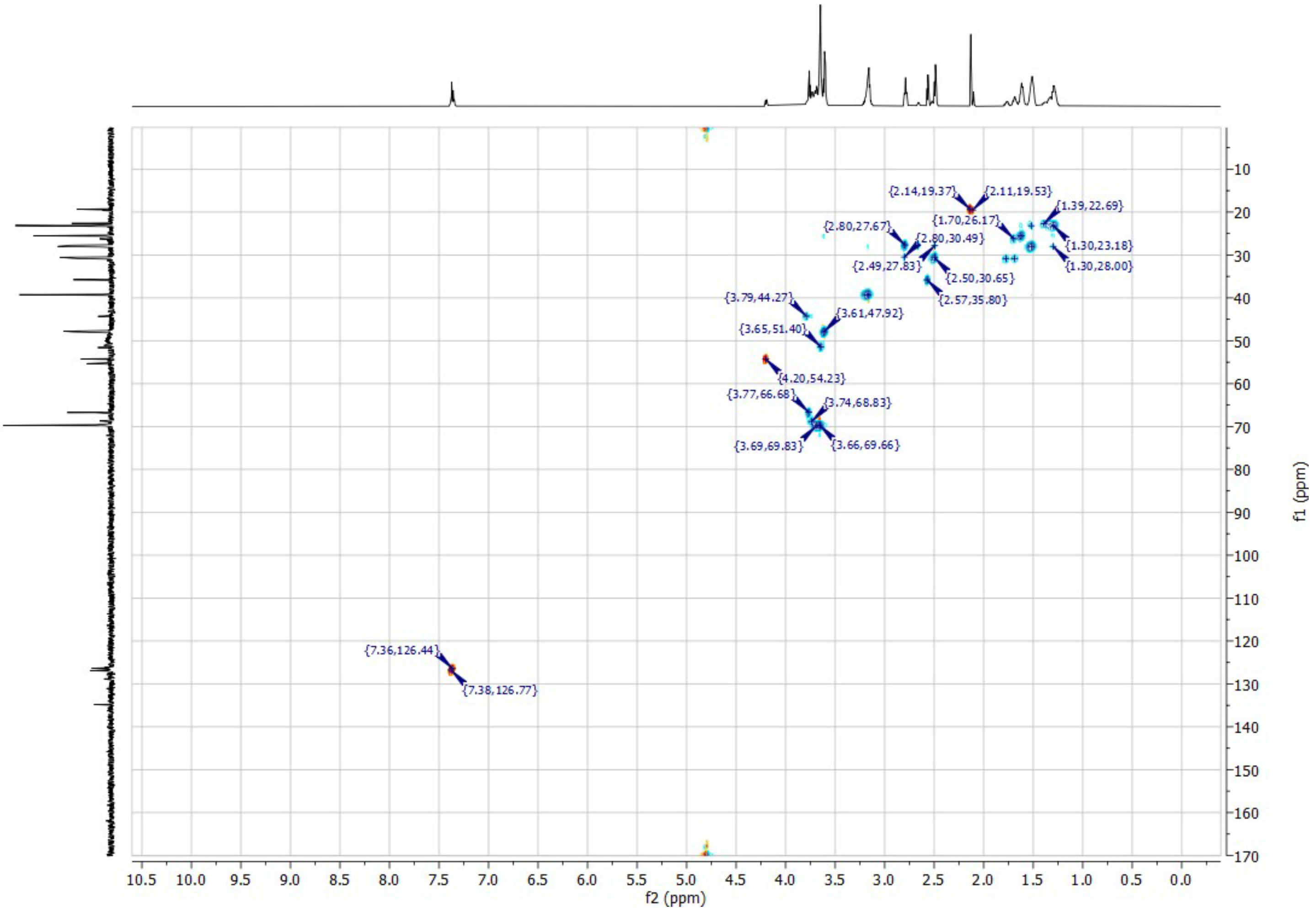

Figure S14. HSQC NMR (700 MHz, D_2O) spectrum from a **3b** solution.

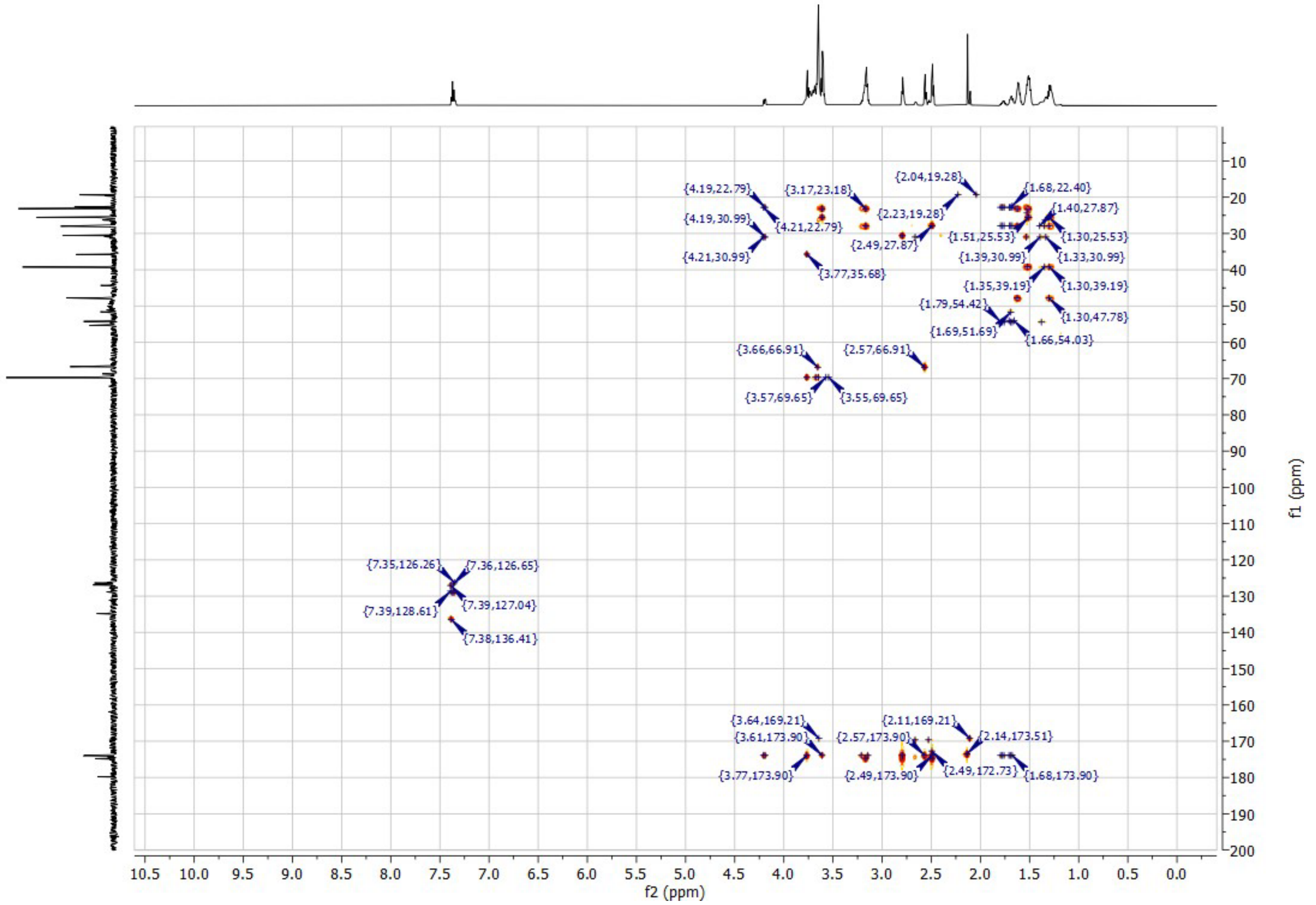

Figure S15. HMBC NMR (700 MHz, D_2O) spectrum from a 3b solution.

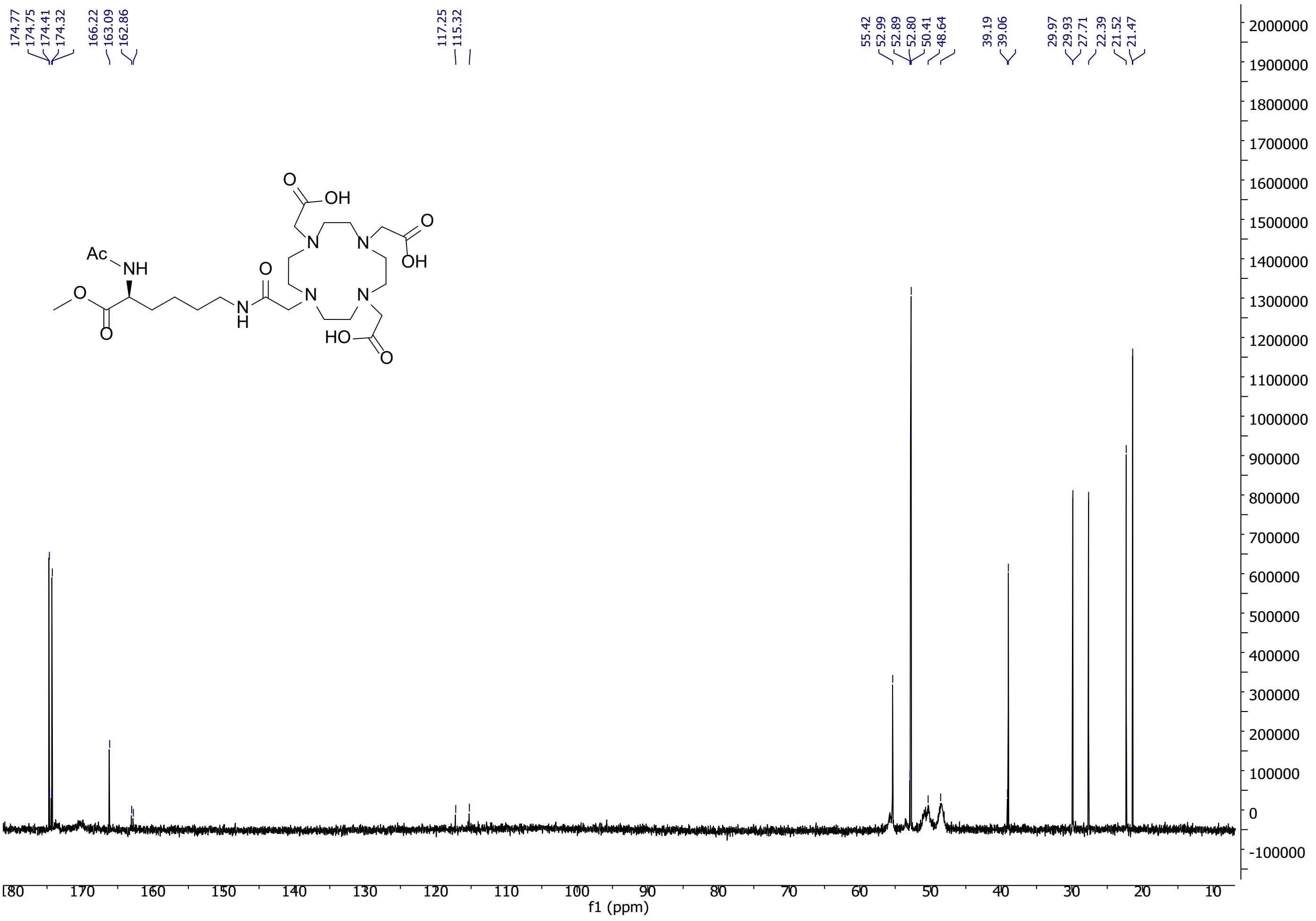

Figure S16. {^{1}H}-^{13}C NMR (600 MHz, D_2O) spectrum from a **8a** solution.

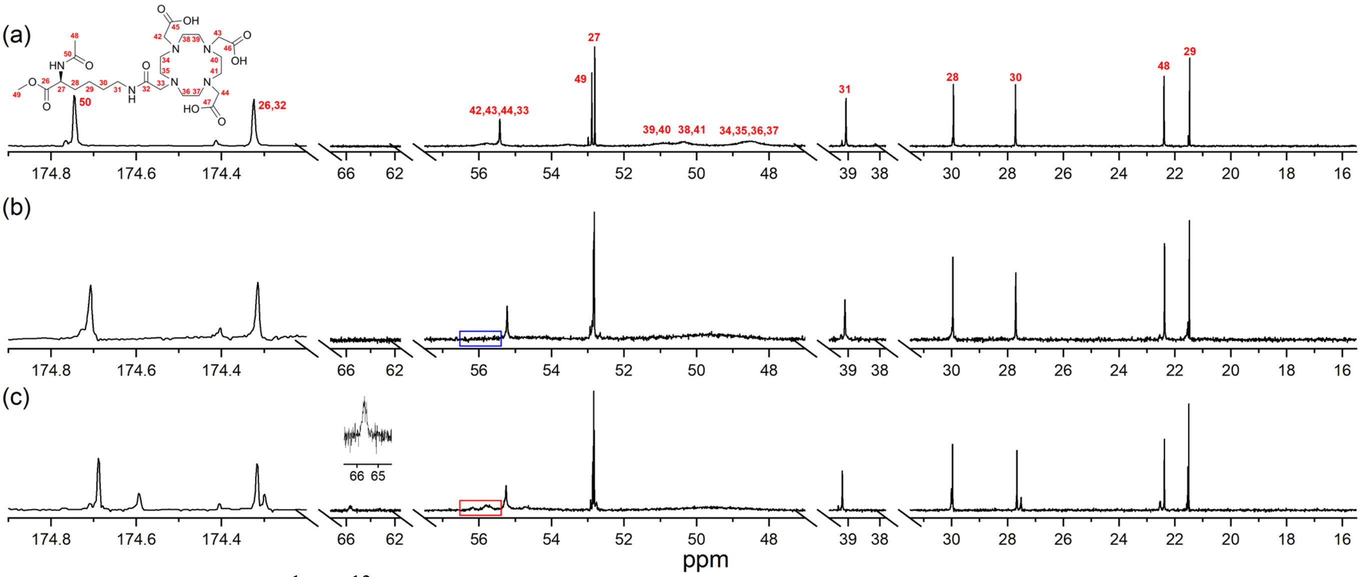

Figure S17. Stacked {^{1}H}-^{13}C NMR (600MHz, D_2O) spectra of solutions of (**a**) **8a**, or (**b**) Zr(IV):**8a**, or (**c**) Lu(III):**8a**. The inset and the region marked with a red box (**c**) show weak signals ascribable to the *endo-/exo-* methylene groups of the DOTA region, which are not evident in (**b**) (region marked with a blue box).

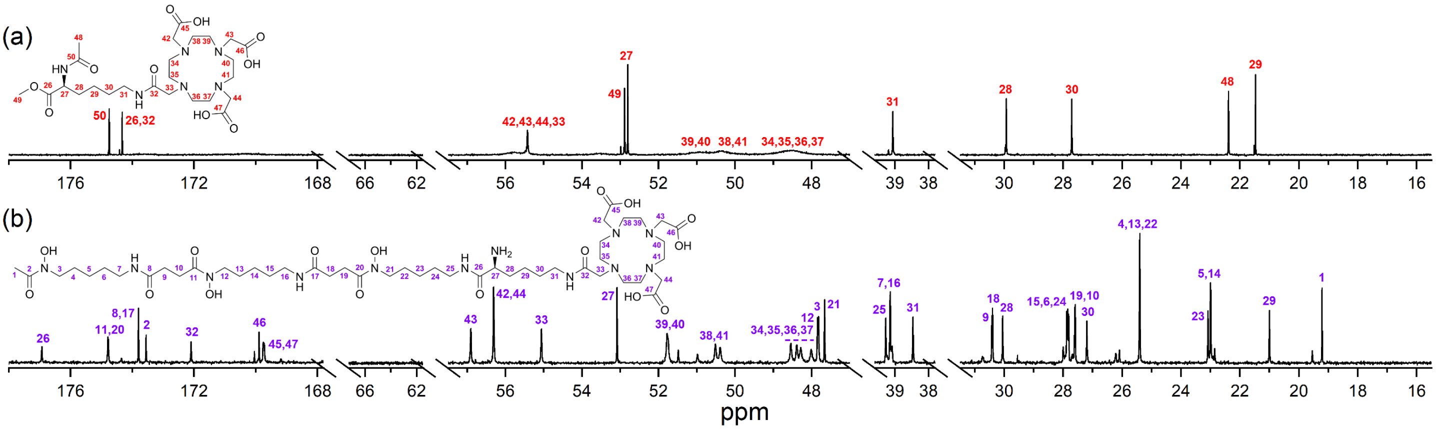

Figure S18. Stacked {^{1}H}-^{13}C NMR (600 MHz, D_2O) spectra from solutions of (**a**) **8a**, or (**b**) **3a**.

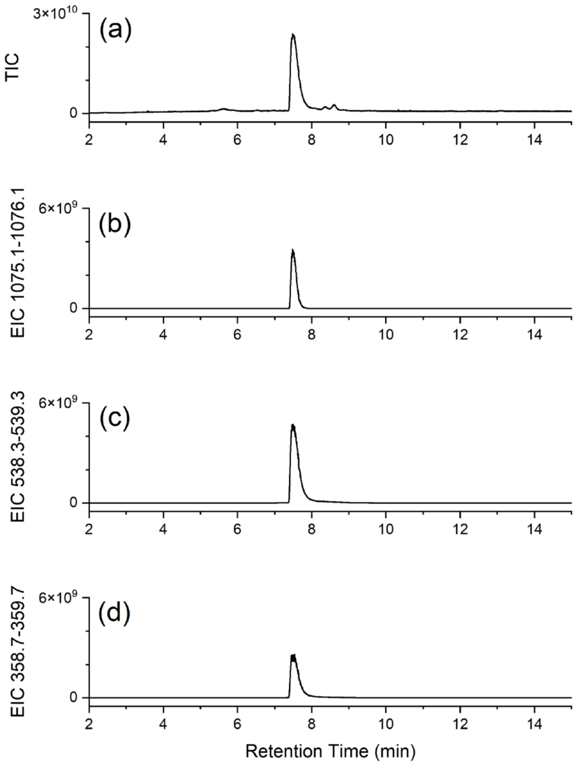

Figure S19. LC-MS traces of **3a** (M = $C_{47}H_{86}N_{12}O_{16}$) shown as (**a**) total ion current (TIC), or as extracted ion chromatograms (EIC) set to report (**b**) $[M+H]^{+}$ (1075.1-1076.1), (**c**) $[M+2H]^{2+}$ (538.3-539.3), or (**d**) $[M+3H]^{3+}$ (358.7-359.7).

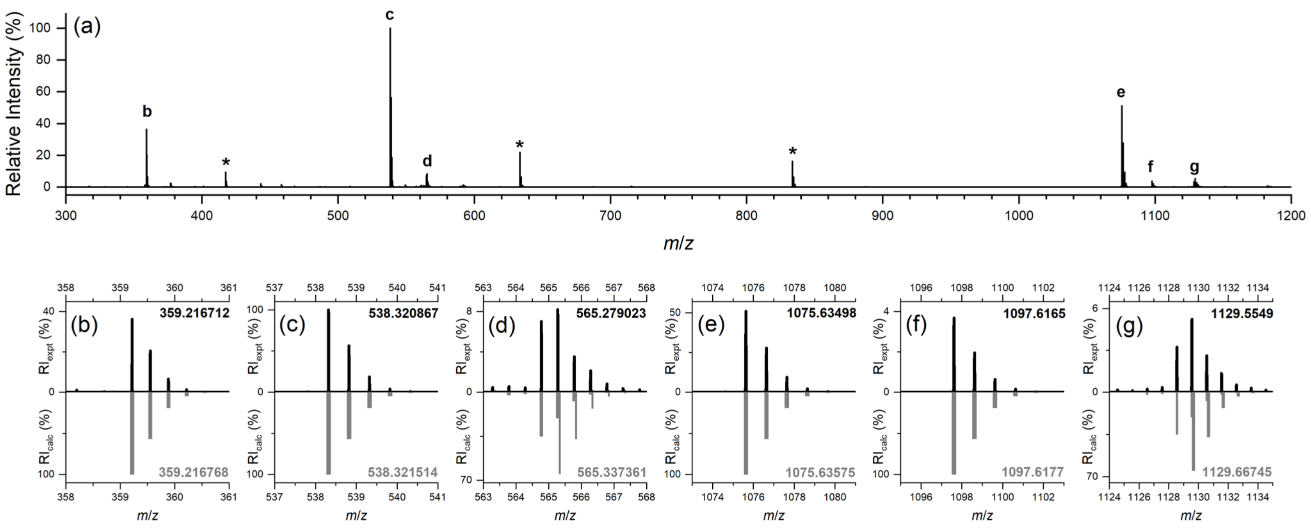

Figure S20. (**a**) High resolution average mass spectrum (R_t = 7.37-7.98 min (refer panel (**a**) of **Figure S19**)) of **3a** (M = $C_{47}H_{86}N_{12}O_{16}$), and experimental (black) and calculated (gray) isotope patterns for marked signals (**b**–**g**) consistent with adducts: (**b**) $[M+3H]^{3+}$, (**c**) $[M+2H]^{2+}$, (**d**) $[M-H+Fe]^{2+}$ (0.35) and $[M+2H+3H_2O]^{2+}$ (0.65), (**e**) $[M+H]^{+}$, (**f**) $[M+Na]^{+}$, or (**g**) $[M-2H+Fe]^{+}$ (0.35) and $[M+H+3H_2O]^{+}$ (0.65). The signals marked with an asterisk are consistent with MS2 fragments of **3a** formed during spectral acquisition (as per scheme below)

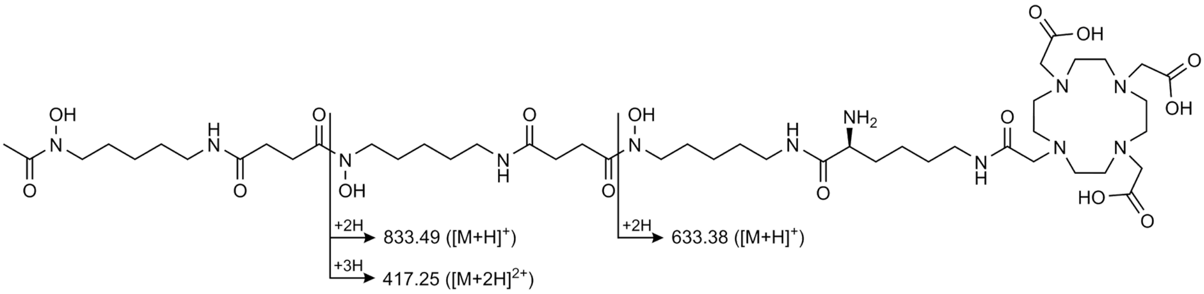

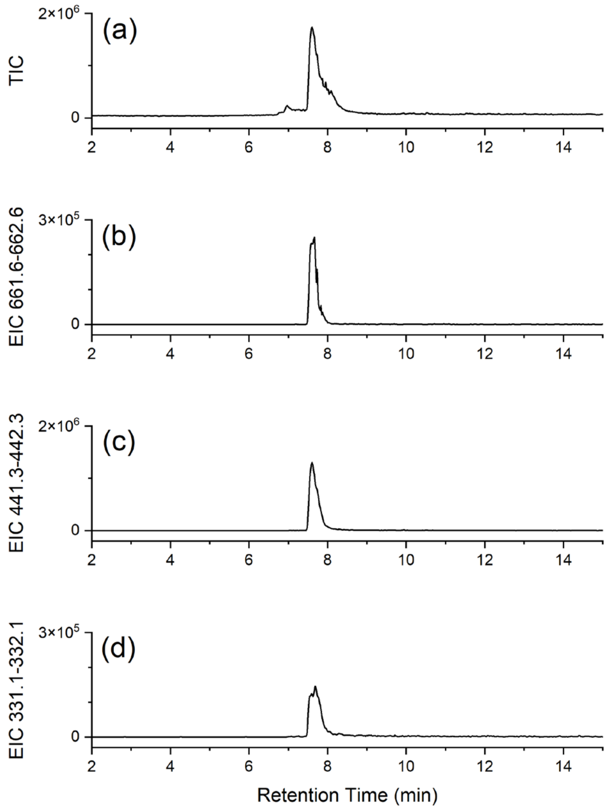

Figure S21. LC-MS traces of **3** (M = $C_{58}H_{107}N_{13}O_{21}$) shown as (**a**) total ion current (TIC), or as extracted ion chromatograms (EIC) set to report (**b**) $[M+2H]^{2+}$ (661.6-662.6), (**c**) $[M+3H]^{3+}$ (441.3-442.4), or (**d**) $[M+4H]^{4+}$ (331.1-332.1).

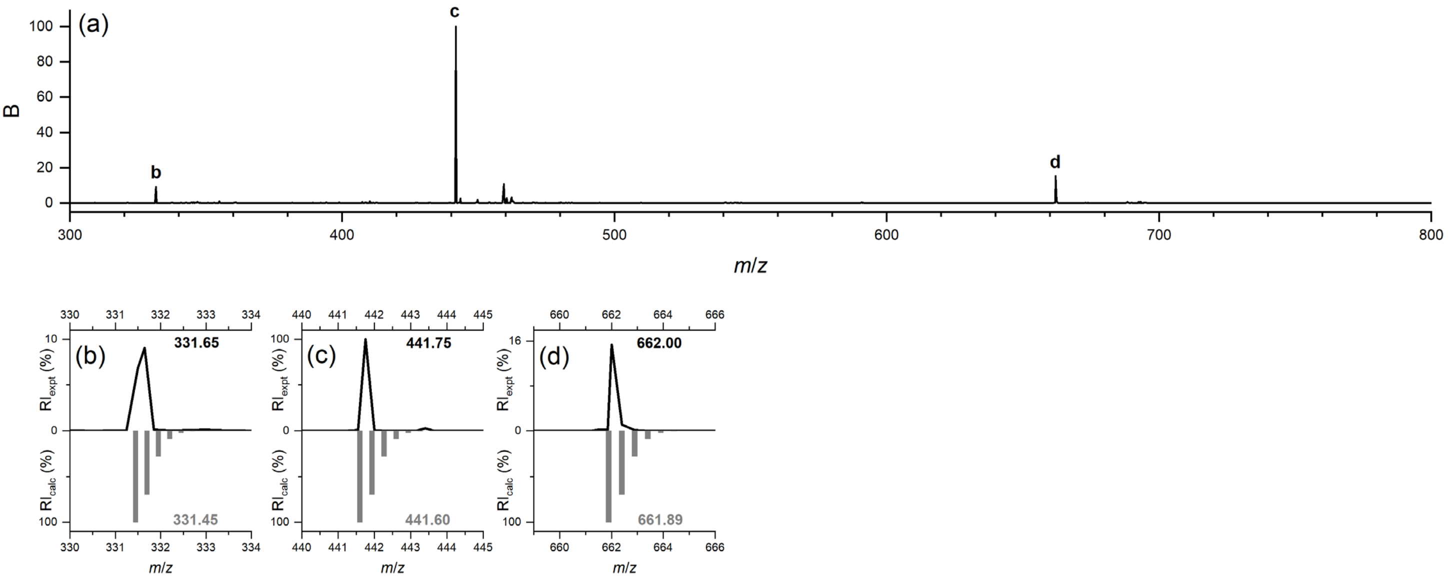

Figure S22. (**a**) Low resolution average mass spectrum (R_t = 6.68-9.24 min (refer panel (**a**) of **Figure S21**)) of **3** (M = $C_{58}H_{107}N_{13}O_{21}$), and experimental (black) and calculated (gray) isotope patterns for marked signals (**b**–**d**) consistent with adducts: (**b**) $[M+4H]^{4+}$, (**c**) $[M+3H]^{3+}$, (**d**) $[M+2H]^{2+}$.

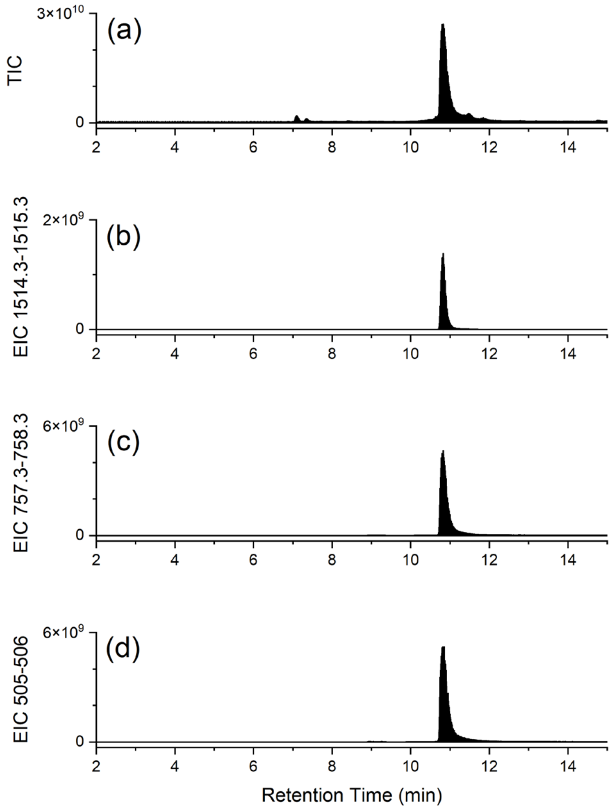

Figure S23. LC-MS traces of **3b** (M = $C_{66}H_{111}N_{15}O_{21}S_2$) shown as (a) total ion current (TIC), or as extracted ion chromatograms (EIC) set to report (b) $[M+H]^+$ (1514.3-1515.3), (c) $[M+2H]^{2+}$ (757.3-758.3), or (d) $[M+3H]^{3+}$ (505-506).

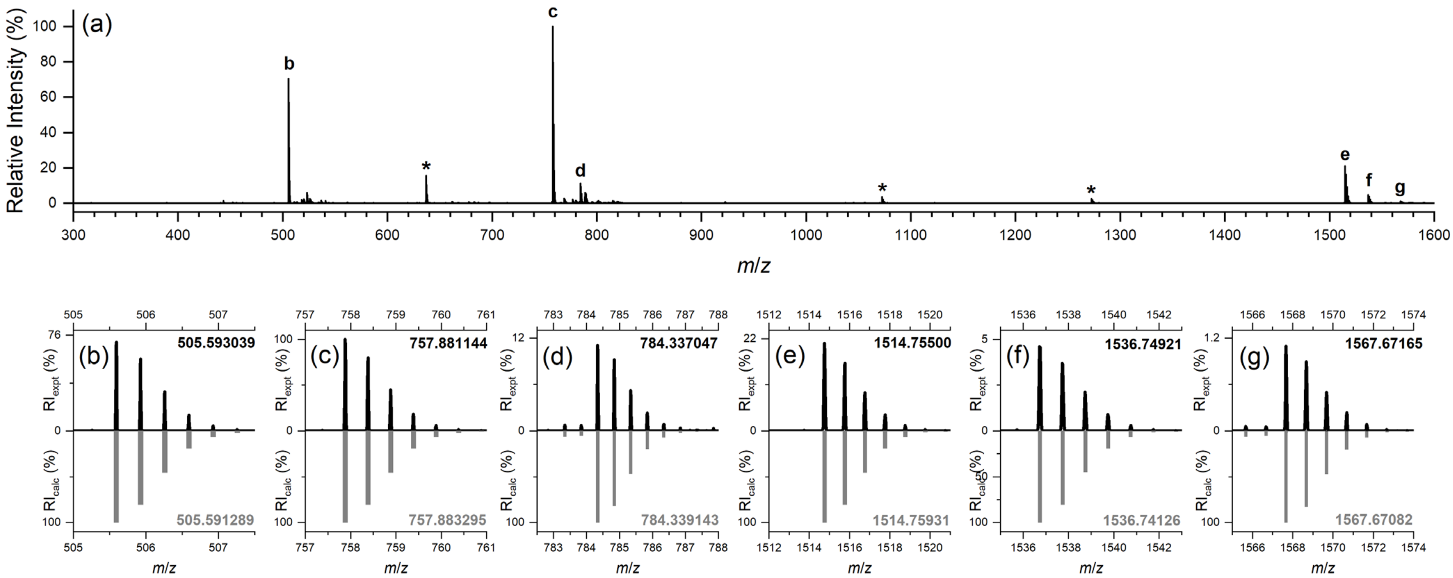

Figure S24. (a) High resolution average mass spectrum (R_t = 9.86-11.93 min (refer panel (a) of **Figure S23**)) of **3** (M = $C_{66}H_{111}N_{15}O_{21}S_2$), and experimental (black) and calculated (gray) isotope patterns for marked signals (**b**–**g**) consistent with adducts: (b) $[M+3H]^{3+}$, (c) $[M+2H]^{2+}$, (d) $[M-H+Fe]^{2+}$, (e) $[M+H]^{+}$, (f) $[M+Na]^{+}$, or (g) $[M-2H+Fe]^{+}$. The signals marked with an asterisk are consistent with MS2 fragments of **3** formed during spectral acquisition (as per scheme below).

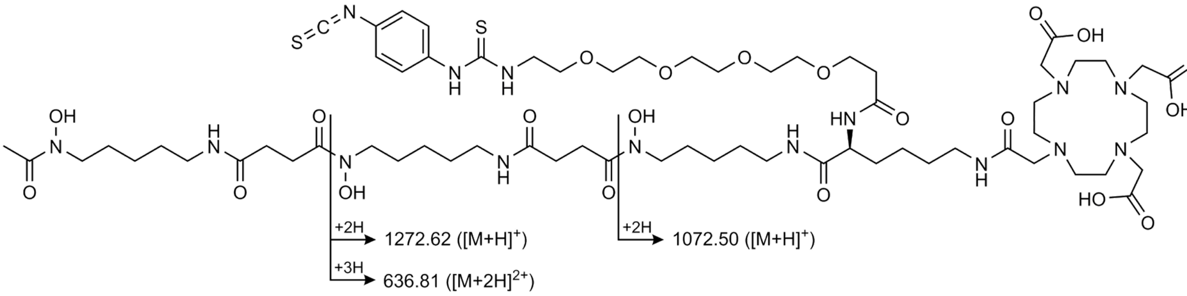

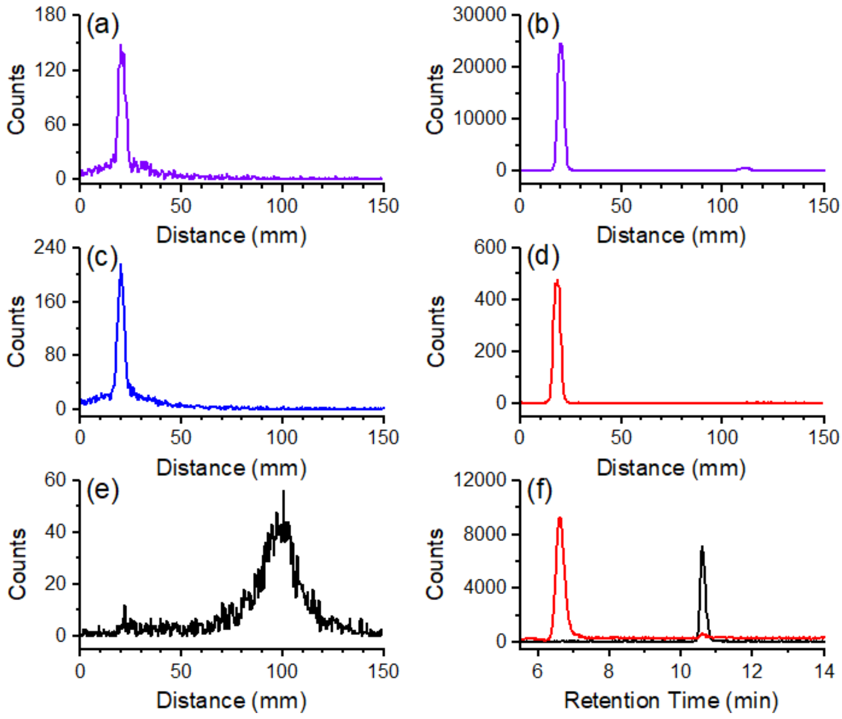

Figure S25. Radio-iTLC (a–e) or radio-SEC-HPLC (f) traces from radiolabelling (a) **D2**-mAb with ^{89}Zr, (b) **D2**-mAb with ^{177}Lu, (c) **1**-mAb with ^{89}Zr, (d) **2**-mAb with ^{177}Lu, (e) **2**-mAb with ^{89}Zr; or (f) **2**-mAb (red) or **1**-mAb (black) with ^{177}Lu. In these experiments, mAb = girentuximab.

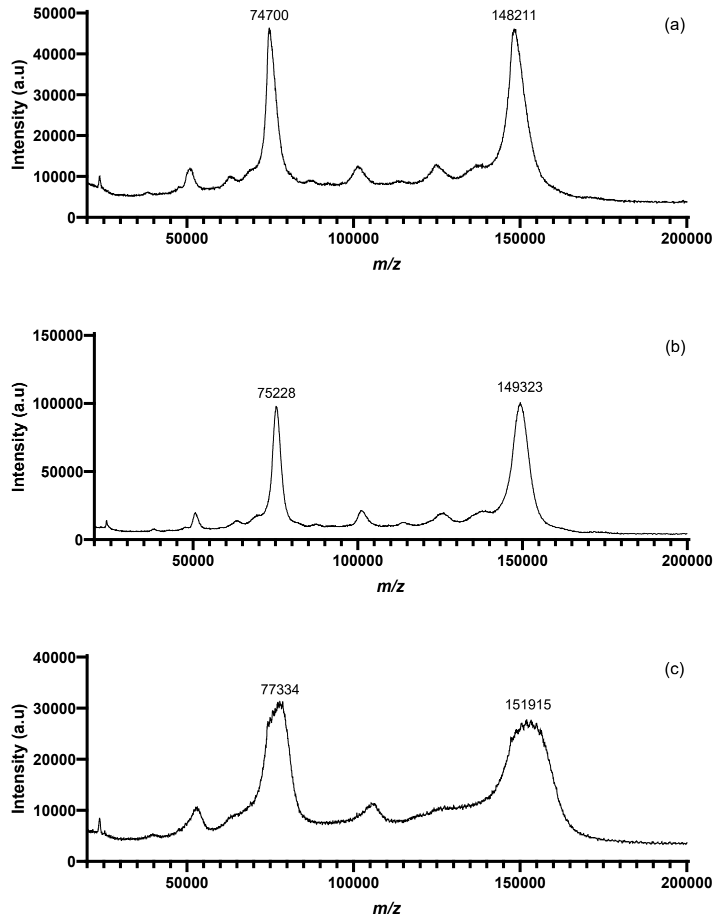

Figure S26. Representative MALDI TOF-MS of **1**-mAb (a), **2**-mAb (b) or **D2**-mAb (c) after reaction at 37 °C for 3 h.

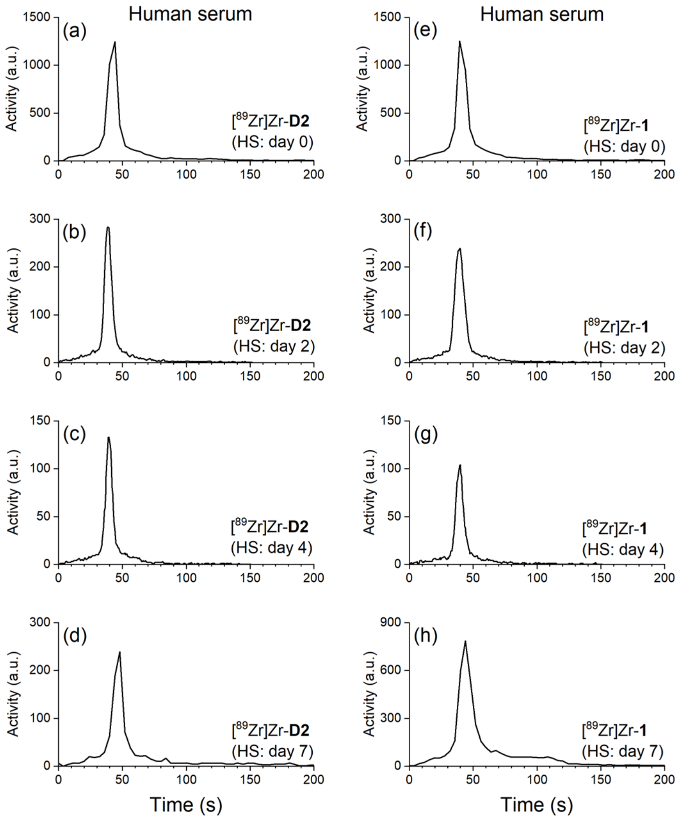

Figure S27. Stability by radio-iTLC of [^{89}Zr]Zr-**D2** (a–d) or [^{89}Zr]Zr-**1** (e–h) in human serum at day 0, 2, 4, 7.

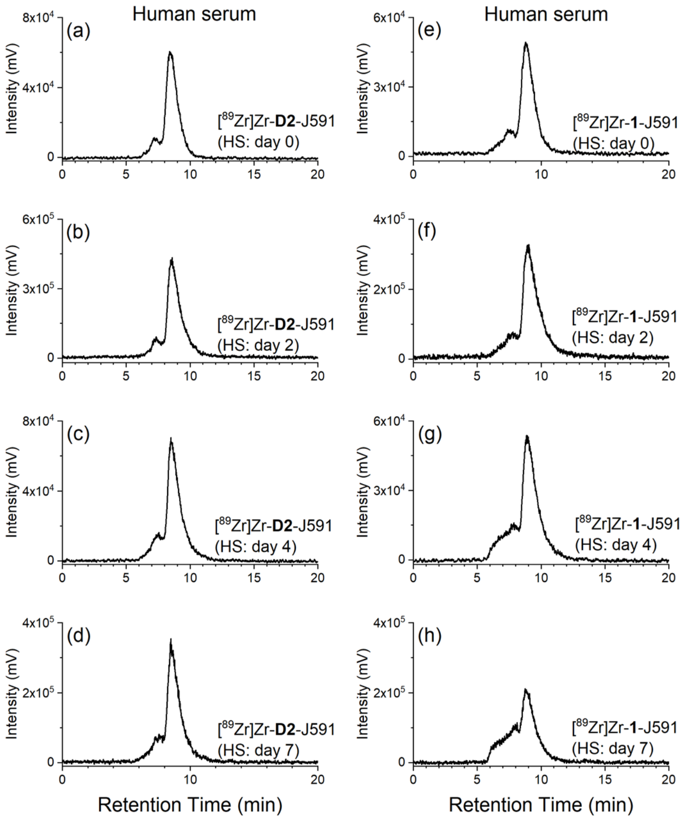

Figure S28. Stability by radio-SEC-HPLC of [^{89}Zr]Zr-**D2**-mAb (a–d) or [^{89}Zr]Zr-**1**-mAb (e–h) in human serum at day 0, 2, 4, 7.

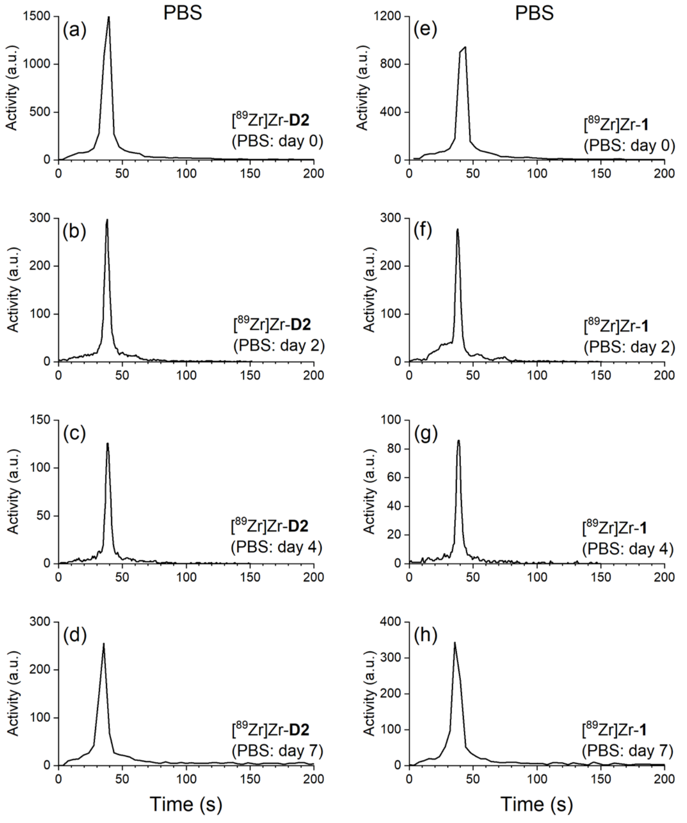

Figure S29. Stability by radio-iTLC of [^{89}Zr]Zr-**D2** (a–d) or [^{89}Zr]Zr-**1** (e–h) in phosphate buffered saline at day 0, 2, 4, 7.

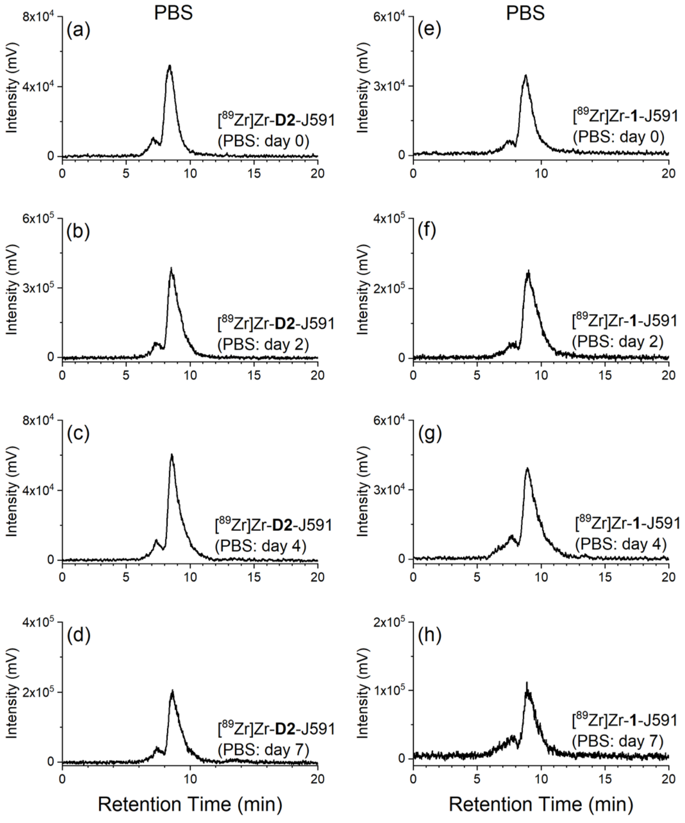

Figure S30. Stability by radio-SEC-HPLC of [^{89}Zr]Zr-**D2**-mAb (a–d) or [^{89}Zr]Zr-**1**-mAb (e–h) in phosphate buffered saline at day 0, 2, 4, 7.

Table S1. C.A.R values for **D2**-mAb, **1**-mAb and 2-mAb conjugates.

Conjugate	Study	C.A.R
**D2**-mAb	^{89}Zr *in vitro* cell assay	~4
	^{177}Lu *in vitro* cell assay	~4
	^{89}Zr *ex vivo* biodistribution (48 h p.i)	~4
	^{89}Zr *ex vivo* biodistribution (120 h p.i)/imaging	~4
	^{177}Lu efficacy study	~4
	^{177}Lu *ex vivo* biodistribution (120 h p.i)/imaging	~4
	^{89}Zr stability study	~4
**1**-mAb	^{89}Zr *in vitro* cell assay	~1
	^{89}Zr *ex vivo* biodistribution (48 h p.i)	~2
	^{89}Zr *ex vivo* biodistribution (120 h p.i)/imaging	~2
	^{89}Zr stability study	~0.5
**2**-mAb	^{177}Lu *in vitro* cell assay	~2
	^{177}Lu efficacy study	~3

Table S2. Raw values for % cell associated activity of all radiolabelled conjugates (n = 3).

Conjugate	% Cell Associated Activity (n=3)
[^{89}Zr]Zr-**D2**-mAb	67.0
	87.5
	59.4
[^{89}Zr]Zr-**1**-mAb	71.5
	74.7
	68.2
[^{177}Lu]Lu-**D2**-mAb	50.5
	47.6
	55.6
[^{177}Lu]Lu-**2**-mAb	60.6
	54.7
	55.6

Table S3. Radiochemical purity of [^{89}Zr]Zr-**D2**-mAb or [^{89}Zr]Zr-**1**-mAb in human serum or PBS at 0, 2, 4, 7 d.

	**[^{89}Zr]Zr-D2-mAb**		**[^{89}Zr]Zr-1-mAb**				**[^{89}Zr]Zr-D2-mAb**		**[^{89}Zr]Zr-1-mAb**	
**Radio-iTLC**	**Human Serum**	**PBS**	**Human Serum**	**PBS**		**Radio-SEC-HPLC**	**Human Serum**	**PBS**	**Human Serum**	**PBS**
Day 0	>99%	>99%	>99%	>99%		Day 0	86.7%	85.5%	82.5%	87.1%
Day 2	>99%	>99%	>99%	>99%		Day 2	86.4%	86.7%	83.2%	86.4%
Day 4	>99%	>99%	>99%	>99%		Day 4	83.6%	86.8%	73.0%	80.4%
Day 7	83.0%	93.6%	76.3%	~95.3%		Day 7	82.5%	80.6%*	61.3%	76.3%

*Sample displayed free ^{89}Zr signal and was incorporated into radiochemical purity calculation.

Table S4. Mouse identifiers and injected dose for *ex-vivo* biodistribution studies. Approximate mass dose calculated using activity measured from initial labelling reaction.

**Compound**	**Mouse ID**	**Injected Actvity (MBq)**	**Molar Activity (GBq/µmol)**	**Approximate Mass Dose (µg)**
[^{177}Lu]Lu-**D2**-mAb (5-day)	D2-Lu-1	4.25	1.85	346
	D2-Lu-2	4.35		354
	D2-Lu-3	3.45		280
[^{89}Zr]Zr-**D2**-mAb (2-day)	D2-Zr-M4	3.59	3.19	169
	D2-Zr-M5	3.50		165
	D2-Zr-M6	3.62		170
[^{89}Zr]Zr-**D2**-mAb (5-day)	D2-Zr-M1	3.12	3.42	137
	D2-Zr-M2	3.37		148
	D2-Zr-M3	3.29		145
[^{89}Zr]Zr-**1**-mAb (2-day)	DFOB-Zr-M4	3.60	3.20	170
	DFOB-Zr-M5	3.46		164
	DFOB-Zr-M6	3.34		158
[^{89}Zr]Zr-**1**-mAb (5-day)	DFOB-Zr-1	3.31	3.36	148
	DFOB-Zr-2	3.33		149
	DFOB-Zr-3	3.33		149

Table S5. Mouse identifiers and injected dose for imaging studies. Approximate mass dose calculated using activity measured from initial labelling reaction.

**Compound**	**Mouse ID**	**Injected Actvity (MBq)**	**Molar Activity (GBq/µmol)**	**Approximate Mass Dose (µg)**
[^{177}Lu]Lu-**D2**-mAb	D2-Lu-1	4.25	1.85	346
	D2-Lu-2	4.35		354
	D2-Lu-3	3.45		280
[^{89}Zr]Zr-**D2**-mAb	D2-Zr-M1	3.12	3.42	137
	D2-Zr-M2	3.37		148
	D2-Zr-M3	3.29		145
[^{89}Zr]Zr-**1**-mAb	DFOB-Zr-1	3.31	3.36	148
	DFOB-Zr-2	3.33		149
	DFOB-Zr-3	3.33		149

Table S6. *In vivo* biodistribution (*n* = 3) of [^{89}Zr]Zr-**1**-mAb and [^{89}Zr]Zr-**D2**-mAb at 4 h, 24 h, 48 h, or 120 h post-injection; or [^{89}Zr]Zr-**D2**-mAb and [^{177}Lu]Lu-**D2**-mAb at 48 h, or 120 h post-injection.

Compound	[89Zr]Zr-DFO-J591	[89Zr]Zr-DFO-J591	[89Zr]Zr-D2-J591	[89Zr]Zr-D2-J591	[89Zr]Zr-DFO-J591	[89Zr]Zr-DFO-J591	[89Zr]Zr-D2-J591	[89Zr]Zr-D2-J591
Time	4 h	4 h	4 h	4 h	24 h	24 h	24 h	24 h
	Mean (n = 3)	SD (n = 3)	Mean (n = 3)	SD (n = 3)	Mean (n = 3)	SD (n = 3)	Mean (n = 3)	SD (n = 3)
Tissue	% ID/g	% ID/g	% ID/g	% ID/g	% ID/g	% ID/g	% ID/g	% ID/g
heart	9.9431	2.501	13.09803	1.76051	5.19537	1.15372	8.073	0.75545
lungs	6.28763	1.19827	9.04027	1.18647	3.96202	0.49427	5.43462	0.60796
liver	16.77167	3.21533	11.3284	1.09724	14.1874	3.15328	9.68223	0.94339
spleen	6.1779	1.02395	6.91277	1.91319	3.72833	1.0571	6.67273	1.76614
right kidney	6.04417	1.2085	8.02203	2.60669	5.35163	0.47621	9.5351	1.28661
left femur	1.13057	0.46645	1.26573	0.36468	1.27847	0.04586	2.07017	0.24163
bladder	1.59897	0.33031	1.9953	0.18727	1.64273	0.0827	2.15677	0.1926
tumour	3.11233	0.65511	5.69457	0.35899	6.25603	0.83919	15.01127	1.21726
Compound	[89Zr]Zr-DFO-J591	[89Zr]Zr-DFO-J591	[89Zr]Zr-D2-J591	[89Zr]Zr-D2-J591	[89Zr]Zr-DFO-J591	[89Zr]Zr-DFO-J591	[89Zr]Zr-D2-J591	[89Zr]Zr-D2-J591
Time	48 h	48 h	48 h	48 h	120 h	120 h	120 h	120 h
	Mean (n = 3)	SD (n = 3)	Mean (n = 3)	SD (n = 3)	Mean (n = 3)	SD (n = 3)	Mean (n = 3)	SD (n = 3)
Tissue	% ID/g	% ID/g	% ID/g	% ID/g	% ID/g	% ID/g	% ID/g	% ID/g
heart	3.87943	1.15093	6.33507	1.10542	2.1903	0.81818	3.34987	0.39916
lungs	3.4262	0.6332	4.6758	0.20565	2.77203	0.62486	3.05422	0.33496
liver	13.81253	2.7144	9.28043	1.16693	14.55427	1.79731	9.55233	1.10727
spleen	5.39517	0.2598	4.74243	0.45733	3.91637	1.28553	4.25887	0.62301
right kidney	5.25977	1.10439	9.0316	1.37839	3.63003	0.66685	6.6245	0.51125
left femur	1.41543	0.4738	2.46427	0.76371	2.2354	0.45701	2.68573	0.27987
bladder	1.46303	0.07394	2.1567	0.10999	0.88883	0.22362	1.3686	0.09398
tumour	14.3233	3.09152	13.39693	1.04049	13.7782	2.45707	15.8161	2.78247
Compound	[89Zr]Zr-D2-J591	[89Zr]Zr-D2-J591	[177Lu]Lu-D2-J591	[177Lu]Lu-D2-J591	[89Zr]Zr-D2-J591	[89Zr]Zr-D2-J591	[177Lu]Lu-D2-J591	[177Lu]Lu-D2-J591
Time	48 h	48 h	48 h	48 h	120 h	120 h	120 h	120 h
	Mean (n = 3)	SD (n = 3)	Mean (n = 3)	SD (n = 3)	Mean (n = 3)	SD (n = 3)	Mean (n = 3)	SD (n = 3)
Tissue	% ID/g	% ID/g	% ID/g	% ID/g	% ID/g	% ID/g	% ID/g	% ID/g
heart	6.33507	1.10542	7.15925	1.73307	3.34987	0.39916	4.35405	1.33874
lungs	4.6758	0.20565	4.77195	2.56519	3.05422	0.33496	2.98533	1.77625
liver	9.28043	1.16693	9.15356	1.26546	9.55233	1.10727	7.30492	0.81882
spleen	4.74243	0.45733	4.45933	0.56266	4.25887	0.62301	2.72759	0.77625
right kidney	9.0316	1.37839	6.78787	0.56134	6.6245	0.51125	5.56563	0.7345
left femur	2.46427	0.76371	4.2689	1.14306	2.68573	0.27987	3.24678	1.24232
bladder	2.1567	0.10999	3.42631	1.05568	1.3686	0.09398	2.75662	0.46175
tumour	13.39693	1.04049	6.85454	1.08654	15.8161	2.78247	11.39659	4.77625

Table S7. *Ex vivo* biodistribution (*n* = 3) of [^{89}Zr]Zr-**1**-mAb and [^{89}Zr]Zr-**D2**-mAb at (**a**) 48 h p.i. or (**b**) 120 h p.i., or (**c**) [^{89}Zr]Zr-**D2**-mAb and [^{177}Lu]Lu-**D2**-mAb (**5**) at 120 h p.i.

Compound	[89Zr]Zr-DFO-J591	[89Zr]Zr-DFO-J591	[89Zr]Zr-D2-J591	[89Zr]Zr-D2-J591	[89Zr]Zr-DFO-J591	[89Zr]Zr-DFO-J591	[89Zr]Zr-D2-J591	[89Zr]Zr-D2-J591
Time	2 d	2 d	2 d	2 d	5 d	5 d	5 d	5 d
	Mean (n = 3)	SD (n = 3)	Mean (n = 3)	SD (n = 3)	Mean (n = 3)	SD (n = 3)	Mean (n = 3)	SD (n = 3)
Tissue	% ID/g	% ID/g	% ID/g	% ID/g	% ID/g	% ID/g	% ID/g	% ID/g
liver	15.14	3.01	5.52	0.53	11.34	1.42	7.38	0.63
spleen	3.49	0.37	2.59	0.52	7.87	5.69	7.34	1.84
kidneys	5.46	0.31	7.91	0.51	4.1	0.8	7.63	1.83
heart	1.45	0.26	1.51	0.38	1.15	0.29	1.35	0.17
lungs	1.64	0.19	1.65	0.4	1.48	0.24	2.18	0.23
blood	2.7	0.41	4.88	0.98	1.54	0.78	2.99	0.55
GI tract	0.71	0.1	0.63	0.12	0.51	0.13	0.57	0.06
tail	1.07	0.07	2.98	0.88	3.16	3.54	2.56	2.74
shoulder	0.51	0.15	0.87	0.25	0.56	0.19	1.01	0.4
knee	1.17	0.53	1.08	0.18	1.15	0.24	2.82	1.04
tumour	9.09	6.66	12.49	3.6	28.13	13.66	31.21	5.23
Compound	[89Zr]Zr-D2-J591	[89Zr]Zr-D2-J591	[177Lu]Lu-D2-J591	[177Lu]Lu-D2-J591				
Time	5 d	5 d	5 d	5 d				
	Mean (n = 3)	SD (n = 3)	Mean (n = 3)	SD (n = 3)				
Tissue	% ID/g	% ID/g	% ID/g	% ID/g				
liver	7.38	0.63	9.4	2.33				
spleen	7.34	1.84	5.96	1.49				
kidneys	7.63	1.83	7.6	1.46				
heart	1.35	0.17	2.26	0.55				
lungs	2.18	0.23	4.85	2.92				
blood	2.99	0.55	4.86	2.37				
GI tract	0.57	0.06	0.92	0.06				
tail	2.56	2.74	3.27	1.23				
shoulder	1.01	0.4	0.98	0.38				
knee	2.82	1.04	2	0.75				
tumour	31.21	5.23	46.87	14.63				

Table S8. Mouse identifiers and dosing for efficacy study using [^{177}Lu]Lu-**D2**-mAb (n=4), [^{177}Lu]Lu-**2**-mAb (n=3) and a vehicle control (n=3). A target of 9 MBq was used for ^{177}Lu groups. Approximate mass dose calculated using activity measured from initial labelling reaction.

Compound	Mouse ID	Injected Actvity (MBq)	Molar Activity (GBq/µmol)	Approximate Mass Dose (µg)
[^{177}Lu]Lu-**D2**-mAb	D2-M1	9.11	1.63	840
	D2-M2	9.38	1.63	865
	D2-M3	9.33	1.63	860
	D2-M4	8.66	1.51	863
[^{177}Lu]Lu-**2**-mAb	DOTA-M1	9.94	1.60	929
	DOTA-M3	8.71	1.61	814
	DOTA-M4	8.75	1.37	959
PBS	PBS-M2	-		-
	PBS-M3	-		-
	PBS-M4	-		-

Table S9 Statistical data from efficacy study using [^{177}Lu]Lu-**D2**-mAb, [^{177}Lu]Lu-**2**-mAb, and PBS vehicle control

[177Lu]Lu-D2-mAb Treatment Group

Therapeutic Efficacy [177Lu]Lu-D2-mAb (Tumour Vol., mm³)				Therapeutic Efficacy [177Lu]Lu-D2-mAb (Mouse Weight, g)			
Days after injection	[177Lu]Lu-D2-mAb (n)	[177Lu]Lu-D2-mAb (AV)	[177Lu]Lu-D2-mAb (SD)	Days after injection	[177Lu]Lu-D2-mAb (n)	[177Lu]Lu-D2-mAb (AV)	[177Lu]Lu-D2-mAb (SD)
2	4	222.25	63.61	2	4	25.85	2.04
6	4	146.75	40.93	6	4	25.58	2.04
9	4	93.50	38.32	9	4	25.60	2.26
13	4	81.00	25.68	13	4	25.90	2.53
16	4	64.50	26.01	16	4	26.25	2.91
20	4	67.00	24.51	20	4	26.63	2.60
23	2	89.50	30.41	23	4	27.68	2.30
27	3	80.33	30.02	26	1	24.30	
30	3	52.33	27.79	27	3	28.87	1.81
33	1	44.00		29	1	24.20	
34	2	41.00	31.11	30	3	27.50	2.43
35	1	35.00		33	1	24.50	
37	2	29.50	33.23	34	3	27.63	2.57
40	3	50.67	35.92	35	1	24.10	
42	4	35.50	22.99	37	4	27.30	2.08
44	4	34.00	26.39	40	4	27.30	2.23
47	4	40.00	35.88	42	4	28.13	2.47
49	4	43.00	33.32	44	4	27.98	2.35
51	3	35.67	24.42	47	4	27.58	1.59
54	2	34.00	31.11	49	4	27.93	2.20
56	3	35.00	20.81	51	4	28.03	1.96
58	3	40.67	27.47	54	3	29.53	1.31
61	2	27.00	26.87	56	4	28.00	2.57
63	3	21.33	22.50	58	4	28.58	2.17
65	3	26.33	26.73	61	3	28.53	1.86
				63	4	27.55	1.55
				65	4	27.63	2.28

[177Lu]Lu-DOTA-mAb Treatment Group

Therapeutic Efficacy [177Lu]Lu-DOTA-mAb (Tumour Vol., mm³)				Therapeutic Efficacy [177Lu]Lu-DOTA-mAb (Mouse Weight, g)			
Days after injection	[177Lu]Lu-DOTA-mAb (n)	[177Lu]Lu-DOTA-mAb (AV)	[177Lu]Lu-DOTA-mAb (SD)	Days after injection	[177Lu]Lu-DOTA-mAb (n)	[177Lu]Lu-DOTA-mAb (AV)	[177Lu]Lu-DOTA-mAb (SD)
1	2	143.00	79.20	1	2	25.05	3.61
2	1	119.00		2	1	24.10	
5	3	154.00	58.40	5	3	23.47	2.29
8	3	94.00	37.99	8	3	23.70	1.65
12	3	68.00	21.66	12	3	23.70	2.46
15	3	60.33	30.01	15	3	23.73	2.34
18	3	61.67	29.26	18	3	23.80	2.15
20	3	49.33	18.50	20	3	24.37	2.32
22	3	50.00	22.34	22	3	24.37	2.40
25	3	56.33	22.74	25	3	24.43	1.85
26	1	67.00		26	1	23.70	
27	2	45.50	38.89	27	2	25.15	3.18
29	3	40.33	17.67	29	3	24.77	2.20
32	2	29.00	11.31	32	2	25.85	2.76
33	1	80.00		33	1	24.30	
34	2	43.50	19.09	34	2	25.95	2.19
35	1	70.00		35	1	25.20	
36	2	36.50	7.78	36	2	26.25	2.19
37	1	53.00		37	1	25.00	
39	2	25.50	13.44	39	2	26.00	2.26
40	1	66.00		40	1	24.60	
41	2	34.50	16.26	41	2	26.65	2.62
42	1	59.00		42	1	24.20	
43	2	41.00	32.53	43	2	25.75	1.91
44	1	49.00		44	1	24.50	
46	1	64.00		46	2	24.15	4.60
47	1	48.00		47	1	24.80	
49	2	53.50	14.85	49	3	24.67	3.10
51	1	29.00		51	1	24.70	
53	1	64.00		53	2	26.50	1.41
55	1	32.00		55	2	26.55	1.77
56	1	14.00		56	1	25.60	
62	1	37.00		57	2	26.75	1.63
63	1	24.00		58	1	25.30	
64	1	40.00		60	2	26.65	1.63
65	1	22.00		62	2	26.20	1.41
				63	1	25.30	
				64	2	26.45	1.34
				65	1	25.60	

PBS Vehicle

Therapeutic Efficacy (PBS) (Tumour Vol., mm³)				Therapeutic Efficacy (PBS) (Mouse Weight, g)			
Days after injection	PBS (n)	PBS (AV)	PBS (SD)	Days after injection	PBS (n)	PBS (AV)	PBS (SD)
12	3	399.67	42.36	12	3	21.50	1.37
15	3	436.67	51.73	15	3	21.30	1.64
17	3	427.00	67.02	17	3	21.00	1.83
19	3	449.67	101.66	19	3	20.97	2.02
23	3	492.33	64.84	23	3	22.07	1.69
30	3	680.67	106.98	30	3	21.77	2.00
34	3	835.00	131.46	34	3	21.67	2.42
37	3	859.00	42.57	37	3	21.63	1.77
40	3	957.00	108.53	40	3	22.43	1.91
42	2	1047.00	267.29	42	2	22.95	0.07
43	1	1004.00		43	1	22.50	
50	1	1144.00		46	1	21.60	
52	1	1159.00		50	1	21.20	
				52	1	20.70	

Table S10. Radiolabelling parameters used in study components.

Conjugate	Study	Concentration$_{conj}$ (mg/mL)	Moles$_{conj}$	Moles$_{isotope}$	Specific Activity$_{10\mu L}$ (MBq)	Total Activity (MBq)	Total Reaction Volume (µL)
**D2**-mAb	^{89}Zr *in vitro* cell assay	6.31	~1.67×10^{-9}	~5.07×10^{-12}	1.24	~7.5	99.8
	^{177}Lu *in vitro* cell assay	3.12	4.72×10^{-9}	9.38×10^{-12}	44.25	7.80	228.6
	^{89}Zr *ex vivo* biodistribution (48 h p.i)	6.31	6.09×10^{-9}	1.22×10^{-11}	1.94	19.4	237.5
	^{89}Zr *ex vivo* biodistribution (120 h p.i)/imaging	10.91	6.76×10^{-9}	2.71×10^{-11}	5.34	23.1	130.5
	^{177}Lu efficacy study (session 1)	9.21	2.69×10^{-8}	5.37×10^{-11}	19.3	43.6	457.8
	^{177}Lu efficacy study (session 2)	8.57	1.01×10^{-8}	2.01×10^{-11}	10.6	15.2	190.1
	^{177}Lu *ex vivo* biodistribution (120 h p.i)/imaging	8.57	1.07×10^{-8}	2.68×10^{-11}	7.7	19.8	214.0
	^{89}Zr stability study	6.31	6.09×10^{-9}	1.22×10^{-11}	1.94	19.4	237.5
**1**-mAb	^{89}Zr *in vitro* cell assay	9.61	~1.69×10^{-9}	~5.07×10^{-12}	1.24	~7.5	86.4
	^{89}Zr *ex vivo* biodistribution (48 h p.i)	3.93	6.76×10^{-9}	1.35×10^{-11}	1.48	~20	393.1
	^{89}Zr *ex vivo* biodistribution (120 h p.i)/imaging	9.80	6.76×10^{-9}	2.71×10^{-11}	5.34	22.7	141.0
	^{89}Zr stability study	3.93	~6.76×10^{-9}	~1.35×10^{-11}	1.48	~20	393.1
**2**-mAb	^{177}Lu *in vitro* cell assay	7.89	5.36e^{-9}	1.07e^{-11}	44.25	7.13	103.6
	^{177}Lu efficacy study (session 1)	7.43	2.68×10^{-8}	5.36×10^{-11}	25.3	43.0	556.8
	^{177}Lu efficacy study (session 2)	7.89	1.01×10^{-8}	2.01×10^{-11}	10.6	14.5	205.2
